# Association between carotid-femoral pulse wave velocity and cardiovascular disease in individuals with moderate blood pressure: a systematic review and individual participant meta-analysis

**DOI:** 10.1136/bmjopen-2025-101368

**Published:** 2025-12-15

**Authors:** Holly Pavey, Angela Wood, Carmel M Mceniery, Majd AlGhatrif, Banafsheh Arshi, Eric Brunner, Chen-Huan Chen, Hao-Min Cheng, Tine W Hansen, M Kamram Ikram, Maryam Kavousi, Diana Kuh, Allison L Kuipers, Edward G Lakatta, Allan Linneberg, Francesco Mattace Raso, Gary F Mitchell, João Maldonado, Anne B Newman, Telmo Pereira, Kaisa Maki-Petaja, Martin Shipley, Ramachandran S Vasan, Andrew Wong, Yoav Ben-Shlomo, Ian B Wilkinson

**Affiliations:** 1 Division of Experimental Medicine and Immunotherapeutics, Department of Medicine, University of Cambridge, Cambridge, UK; 2 Institute of Health Economics, Medical University of Innsbruck, Innsbruck, Austria; 3 British Heart Foundation Cardiovascular Epidemiology Unit, Department of Public Health and Primary Care, University of Cambridge, Cambridge, UK; 4 Victor Phillip Dahdaleh Heart and Lung Research Institute, University of Cambridge, Cambridge, UK; 5 British Heart Foundation Centre of Research Excellence, University of Cambridge, Cambridge, UK; 6 National Institute for Health and Care Research Blood and Transplant Research Unit in Donor Health and Behaviour, University of Cambridge, Cambridge, UK; 7 Health Data Research UK Cambridge, Wellcome Genome Campus and University of Cambridge, Cambridge, UK; 8 Cambridge Centre of Artifical Intelligence in Medicine, University of Cambridge, Cambridge, UK; 9 Laboratory of Cardiovascular Science, National Institute on Aging, National Institutes of Health, Bethesda, Maryland, USA; 10 Department of Medicine, Johns Hopkins School of Medicine, Baltimore, Maryland, USA; 11 Department of Epidemiology, Erasmus MC University Medical Center, Rotterdam, The Netherlands; 12 Institute of Epidemiology and Health Care, University College London, London, UK; 13 School of Medicine, National Yang-Ming University, Taipei, Taiwan; 14 PhD Program of Interdisciplinary Medicine (PIM), National Yang Ming Chiao Tung University College of Medicine, Tapei, Taiwan; 15 Division of Faculty Development, Taipei Veterans General Hospital, Taipei, Taiwan; 16 Center for Evidence-based Medicine, Taipei Veterans General Hospital, Taipai, Taiwan; 17 Center for Clinical Research and Prevention, Bispebjerg and Frederiksberg Hospital, Copenhagen, Denmark; 18 Steno Diabetes Center Copenhagen, Herlev, Capital Region of Denmark, Denmark; 19 Department of Clinical Medicine, University of Copenhagen, Copenhagen, Denmark; 20 Department of Epidemiology and Neurology, Erasmus MC, Rotterdam, The Netherlands; 21 MRC Unit for Lifelong Health and Ageing at UCL, London, UK; 22 Department of Epidemiology, University of Pittsburgh, Pittsburgh, Pennsylvania, USA; 23 Cardiovascular Engineering Inc, Norwood, Massachusetts, USA; 24 Clinica de Aveleira, Instituto de Investigação e Formação Cardiovascular, Coimbra, Portugal; 25 Department of Tecnologia, Polytechnic University of Coimbra, Coimbra, Portugal; 26 Department of Medicine, Boston University, Boston, Massachusetts, USA; 27 University of Texas Health Science Center, San Antonio, Texas, USA; 28 NHLBI FHS, Framingham, Massachusetts, USA; 29 Population Health Sciences, University of Bristol, Bristol, UK; 30 Cambridge Clinical Trials Unit, Cambridge, UK

**Keywords:** Meta-Analysis, Hypertension, EPIDEMIOLOGY

## Abstract

**Abstract:**

**Objectives:**

The predictive value of carotid-femoral pulse wave velocity (cfPWV) for cardiovascular (CV) events in individuals with blood pressure (BP) 120–159/80–99 mm Hg, where more accurate risk stratification has the greatest clinical effect, is unknown. This study aims to determine whether cfPWV improves the prediction of CV events beyond traditional risk factors in individuals with moderate BP.

**Design:**

A systematic review and meta-analysis.

**Data sources:**

PubMed and EMBASE were searched through April 2023.

**Eligibility criteria:**

We included prospective, population-based cohort studies with ≥1 year follow-up that directly measured cfPWV as an index of arterial stiffness and reported incident CV disease (CVD), atherosclerotic CVD (ASCVD), coronary heart disease, stroke or all-cause mortality outcomes.

**Data extraction and synthesis:**

Individual participant data from 11 cohorts (n=15 987) were harmonised and analysed using two-stage random-effects meta-analysis. Incremental predictive and clinical utility analyses compared 10-year risk models with and without cfPWV.

**Results:**

There were 1279 first atherosclerotic CV events over a median follow-up of 9.9 years. A 1-SD increase in log_e_(cfPWV) was associated with a 1.21-fold (95% CI 1.08 to 1.36) increase in risk of ASCVD. Adding cfPWV to traditional risk factors improved ASCVD prediction: change in discrimination (C-index): 0.0048 (95% CI 0.0002 to 0.0094), p=0.041. In hypothetical populations of 100 000 individuals with moderate BP, cfPWV-guided treatment could reduce event rates by 2.7% and 3.1% under European and US guidelines, respectively.

**Conclusions:**

Adding cfPWV to traditional CV risk factors may improve the prediction and classification of first CV events in individuals with moderate BP. Additional screening with cfPWV could enhance risk stratification for antihypertensive treatment initiations.

**STRENGTHS AND LIMITATIONS OF THIS STUDY**

- This study provides an up-to-date meta-analysis examining the association between carotid-femoral pulse wave velocity (cfPWV) and cardiovascular disease in the general population.
- The outcome focused on is the clinically relevant endpoint used in the current US and European hypertension guidelines.
- The study focuses on the clinically relevant group of individuals with the highest potential for benefit regarding risk stratification, those with moderate BP, who typically span the threshold for antihypertensive treatment initiation.
- Though statistically significant, C-index improvements were marginal, raising questions about clinical relevance.
- Novel risk models, including cfPWV, were derived and internally validated.

## Introduction

 Hypertension is the most important risk factor for premature death and morbidity globally. Sustained hypertension, defined as a blood pressure (BP) of ≥140/90 mm Hg, affects around one-third of the adult population, and the benefits of treatment are firmly established. However, even an aggressive control of BP to below 120/80 mm Hg does not normalise cardiovascular (CV) risk in middle-aged and older adults.^1^ Likewise, compared with contemporaneous BP, measurements of midlife BP seem more important in predicting left ventricular structure and function in later life, even within non-hypertensive populations.^2 3^ These observations suggest that irreversible end-organ damage accumulates over time and that earlier intervention in prehypertensive or borderline hypertensive individuals may prevent damage and improve vascular health in later life—‘healthy ageing’. Given the large number of individuals who fall into these categories, and the number needed to treat to prevent one event is very high in this population, pharmacological treatment would need to be targeted at those who have increased susceptibility or higher CV risk. Several potential risk markers have been proposed, including aortic stiffness. Aortic stiffness not only assesses accumulated end-organ (aortic) damage but also drives the rise in systolic BP (SBP) with ageing.^4 5^

Many novel biomarkers have been assessed for improving CV risk prediction. However, as established models already have very good risk discrimination, finding a biomarker that adds value and is worth measuring remains challenging. Carotid-femoral pulse wave velocity (cfPWV) is the gold standard non-invasive measure of arterial stiffness, which can be assessed cheaply and easily with little training.^6^ A few studies have shown that cfPWV predicted incident hypertension, even in normotensive individuals, suggesting that cfPWV may also act as a potential early mechanism driving BP and CV risk.^7 8^

Together with its predictive value, these aspects make cfPWV a promising biomarker for inclusion in CV risk prediction algorithms, which was recognised in the European Society of Cardiology (ESC)/European Society of Hypertension guidelines from 2013 to 2018,^9^ when the recommendation was downgraded due to lack of evidence.^10^ In the 2024 guidelines, the ESC discusses cfPWV as an optional secondary measure.^11^ The American College of Cardiology/American Heart Association (ACC/AHA) in 2010 stated that further research into arterial stiffness is needed before including this measurement in their guidelines.^12^ Therefore, we aim to provide more evidence to support the value of cfPWV in CV risk prediction.

A previous meta-analysis showed that cfPWV is independently associated with an increased risk of CV events.^13^ The utility of cfPWV appeared greater in individuals aged under 60 years, suggesting that it may be a useful biomarker in younger individuals at low risk or moderate risk of CV events. However, the predictive value of cfPWV in low-risk or moderate-risk individuals, those with BP 120–160/80–100 mm Hg (referred to as ‘individuals with moderate BP’ throughout this manuscript), could not be quantified. Other previous meta-analyses have also shown cfPWV as an independent and accurate predictor of CV disease (CVD).^14 15^

The aim of the present analysis was to determine the utility of cfPWV in improving risk assessment and treatment classification in a group of individuals with moderate BP without previous CV events, beyond that afforded by established CV risk factors.

## Methods

### Study design

A systematic search was conducted following the Preferred Reporting Items for Systematic Reviews and Meta-Analyses 2009 guidelines^16^, and PubMed and EMBASE were searched through April 2023 (see online supplemental tables S1–S4 and figures S1 and S2). Studies were included if they: (1) had a population-based cohort design with ≥1 year follow-up, (2) had arterial stiffness assessed by direct measurement of cfPWV, (3) provided relevant outcome data on CVD, atherosclerotic CVD (ASCVD), coronary heart disease (CHD), stroke and all-cause mortality (online supplemental table S5), with fatal and non-fatal differentiation preferred but not required and (4) included ≥450 participants to ensure sufficient events for risk estimation and generalisability.

Anonymised individual-level data, including CV risk factors and outcomes, were requested from each study. Individuals with moderate BP (defined throughout as SBP 120–159 mm Hg/DBP80–99 mm Hg) were included in the analysis. There were no exclusions based on antihypertensive medication use, but medication use was adjusted for in all analyses. Individuals with a history of CVD, SBP<120 mm Hg or DBP<80 mm Hg or SBP≥160 mm Hg or DBP≥100 mm Hg were excluded. Further details are provided in online supplemental method 1.

### Patients and public involvement

None.

### Statistical analyses

Baseline characteristics were reported as mean±SD (or median (IQR) if skewed) for continuous variables and frequency (%) for categorical variables. Due to positive skewness and to account for heterogeneity in cfPWV study protocols across cohorts, cfPWV values were log_e_-transformed and standardised within each study (z-scores). This within-study standardisation allowed harmonisation of cfPWV values across cohorts by placing them on a comparable relative scale, thereby enabling pooled analyses. Effect estimates, therefore, represent the change in risk of the outcome per 1-SD higher log_e_-transformed cfPWV within each study.

The primary outcome measure was ASCVD (including all stroke events, fatal CHD (ischaemic heart disease) and non-fatal myocardial infarction). Other exploratory outcome measures considered were CVD (fatal and non-fatal CHD, fatal and non-fatal stroke and heart failure as defined in the study), CHD, stroke and all-cause mortality, as well as fatal and non-fatal subcategories of these endpoints (online supplemental table S5). Fatal events were defined by each study according to its own criteria, typically based on death certificates, hospital records or adjudication by study investigators. We used these study-designated classifications to harmonise fatal and non-fatal outcomes across cohorts. No multiplicity was accounted for when analysing the exploratory endpoints; therefore, results regarding CVD and other exploratory outcomes should be interpreted with this in mind. The main analyses were performed on the complete-case population, defined as individuals with complete risk factors required in the full model. In one study, not all risk factors were measured, and so this study only contributed to the sensitivity analyses using multiple imputation (online supplemental method 1).

Cox proportional hazards models were fitted within each study, with study-specific HRs pooled using random-effects meta-analysis. For each outcome, four Cox proportional hazards models were fitted, allowing for different combinations of risk factors (table 1). Individuals taking antihypertensive medications were included in this analysis, this was accounted for as a covariate in the fully adjusted models. Subgroup analyses were conducted in the following prespecified subgroups: age categories (<60 or ≥60 years), smoking status (current or other), sex, BP categories (120–139 mm Hg/80–89 mm Hg or 140–159 mm Hg/90–99 mm Hg), diabetes status at baseline, prescribed antihypertensive medication at baseline and ethnicity (white or other) for both ASCVD and CVD. Funnel plots and Egger’s tests were used to assess potential small-study effects or selection bias. Linearity of the association between standardised log(cfPWV) and outcomes in Cox proportional hazards models was assessed via martingale residual plots, and the proportional hazards assumption was evaluated using Schoenfeld residuals plotted against time. Sensitivity analyses were based on (1) using only studies contributing to all endpoints, (2) using untransformed cfPWV, (3) using different definitions of moderate risk (as defined by established 10 year risk scores, including pooled cohort equations (PCEs), Systemic COronary Risk Evaluation 2 (SCORE2) and the Framingham CVD risk equation) and (4) using multiple imputation for missing predictor values (see online supplemental method 1). Missing individual-level data were imputed using the jomo package with a random-effects model, including event outcomes and the Nelson-Aalen estimator of survival time.^17^ Participants with missing outcomes or follow-up were censored at their last available examination date. Further details can be found in online supplemental method 1.

**Table 1:** HRs for a 1-SD higher log_e_(cfPWV) (n=15 987)

	HR (95% CI) for log_e_(cfPWV)			
	Model 1	Model 2	Model 3	Model 4
	Crude*	Model 1 with sex, MAP and heart rate	Model 2 with age	Model 1 with established CV risk factors†
Primary outcome				
ASCVD	1.72 (1.45 to 2.04)	1.70 (1.42 to 2.04)	1.25 (1.12 to 1.40)	1.21 (1.08 to 1.36)
Exploratory outcomes				
All CV events‡§	1.74 (1.46 to 2.09)	1.73 (1.43 to 2.09)	1.23 (1.14 to 1.33)	1.20 (1.10 to 1.31)
Fatal CV events‡	2.02 (1.53 to 2.68)	2.03 (1.53 to 2.69)	1.25 (1.07 to 1.47)	1.22 (1.09 to 1.38)
Non-fatal CV events§	1.60 (1.31 to 1.96)	1.57 (1.27 to 1.94)	1.24 (1.12 to 1.37)	1.19 (1.07 to 1.33)
All CHD‡§	1.66 (1.40 to 1.97)	1.64 (1.36 to 1.98)	1.25 (1.11 to 1.41)	1.19 (1.07 to 1.33)
Fatal CHD‡	2.20 (1.68 to 2.88)	2.16 (1.60 to 2.93)	1.34 (1.13 to 1.59)	1.29 (1.09 to 1.53)
Non-fatal CHD‡	1.50 (1.27 to 1.78)	1.48 (1.23 to 1.77)	1.23 (1.08 to 1.40)	1.16 (1.03 to 1.31)
All stroke‡§	1.66 (1.39 to 1.98)	1.64 (1.36 to 1.98)	1.23 (1.09 to 1.39)	1.20 (1.07 to 1.34)
Fatal stroke‡	2.05 (1.52 to 2.76)	2.02 (1.51 to 2.70)	1.34 (1.12 to 1.60)	1.31 (1.10 to 1.57)
Non-fatal stroke§	1.61 (1.32 to 1.96)	1.58 (1.28 to 1.95)	1.20 (1.04 to 1.39)	1.18 (1.02 to 1.36)
All cause-mortality‡	1.76 (1.45 to 2.13)	1.74 (1.42 to 2.13)	1.12 (1.07 to 1.17)	1.13 (1.08 to 1.18)

* Crude HR is unadjusted.

† Age, sex, SBP, total cholesterol, smoking status, HDLC, diabetes status and antihypertensive medications.

‡ The EDIVA study does not contribute to fatal events due to short follow-up.

§ ACCT, BLSA and Kinmen studies do not have data of non-fatal events.

ACCT, The Anglo Cardiff Collaboration Trial; ASCVD, atherosclerotic cardiovascular disease; BLSA, Baltimore Longitudinal Study of Ageing; cfPWV, carotid-femoral pulse wave velocity; CHD, coronary heart disease; CV, cardiovascular; HDLC, high-density lipoprotein cholesterol; MAP, mean arterial pressure; SBP, systolic blood pressure.

Models with and without the addition of log_e_(cfPWV) were compared using discrimination (C-index) and reclassification measures (online supplemental method 1).^18^

We examined ASCVD and CVD risk in individuals with moderate BP by categorising BP and cfPWV, dichotomised at 9 m/s (approximate median). HRs were calculated for subgroups relative to the reference (BP 120–139 mm Hg/80–89 mm Hg, cfPWV≤9 m/s): BP<140/90 mm Hg, cfPWV>9 m/s; BP 140–159 mm Hg/90–99 mm Hg, cfPWV≤9 m/s; and BP 140–159 mm Hg/90–99 mm Hg, cfPWV>9 m/s. 95% CIs were estimated for each group (including the reference group) and corresponded to the amount of information underlying each group.^19 20^

### Derivation, validation and public health modelling of novel cfPWV risk scores

Three sex-specific ASCVD risk scores were developed for direct comparison with recalibrated PCEs^21^ and the SCORE2 (Older-Persons (SCORE2-OP)) equations.^22 23^ Each score includes the same predictor variables as its respective model and applies to individuals aged 40–79 years without a history of CVD.

The novel risk models were compared with recalibrated established risk equations as described in the supplement (online supplemental method 2). The PCEs were recalibrated using calibration-in-the-large by adjusting the baseline survival with a uniform correction factor to reflect event rate differences.^24^ SCORE2 and SCORE2-OP were recalibrated to the ESC-defined low-risk region assigned to most Western European countries, assuming similar risk profiles in the USA. This involved updating the baseline survival with age-specific and sex-specific ASCVD rates and mean risk factors, while keeping the original HRs.^22^ Internal validation with bootstrapping^25 26^ was performed to account for any optimism^27^ in estimating the C-indices, Brier scores, calibration^28^ and reclassification measures (online supplemental method 3).

Population health modelling assessed the clinical impact of our novel cfPWV risk models for ASCVD-based US and European guidelines (online supplemental method 4). An initial policy of antihypertensive medication for individuals at ≥10% predicted 10-year risk, as recommended by the ACC/AHA, was assumed for comparison with the US model.^29^ Age-specific and sex-specific thresholds of predicted risk as defined by the ESC were assumed for comparison with the European models.^10^,^30^ The model assumed age-specific and sex-specific ASCVD event rates based on observed cohort data and standard population distributions (US or European). Additionally, it was assumed that the treatment with antihypertensive medications was allocated per current guidelines and reduced the risk of ASCVD by 20%.^31 32^

Further details on model derivation, validation and population health modelling are provided in online supplemental methods 2–4 respectively. Analyses were performed using Stata, V.15.1 and R, V. 4.0.3.

## Results

12 studies met the inclusion criteria (online supplemental figures S1 and S2),^33^,^46^ with sample sizes ranging from 490 to 2790. Seven were based in Europe, four in North America and one in Taiwan. One small study (n=333 (complete case population without CVD history)) lacked two covariates and was excluded from the main analyses. All studies provided fatal CVD, CHD and stroke data; eight of the studies additionally had data on non-fatal CVD, CHD and stroke events.

The primary analyses included information from 15 987 individuals with moderate BP from 11 studies. The mean (±SD) age at baseline was 64±2 years; 4439 (35%) individuals were under 55 years, 55% of individuals were male, 16% were smokers, 27% were taking antihypertensive medications and the mean (±SD) BP was 135±3 mm Hg/79±3 mm Hg. There were 1279 first ASCVD events and 1796 first CV events (1093 CHD and 692 stroke events); the median follow-up was 9.9 years (median 1.6–19.9 years). The median cfPWV ranged from 6.7 to 11.2 m/s across studies. All other continuous variables remained relatively consistent across all studies. Online supplemental tables S6–S8 detail cfPWV measurement methods, baseline characteristics and event counts across studies.

### cfPWV as a novel predictor

**Figure 1:**
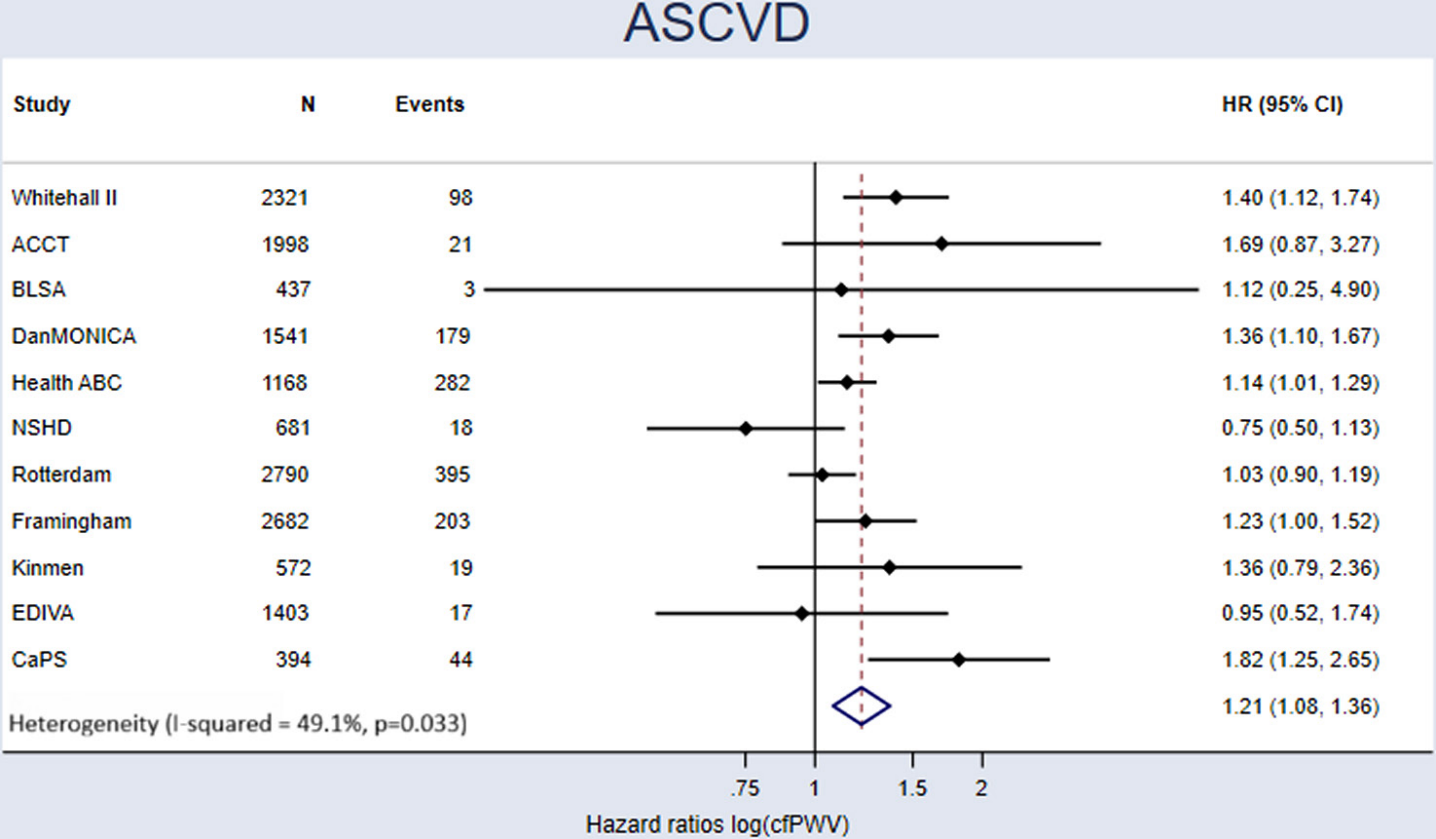
Forest plot of the HRs for the effect of a 1-SD higher log_e_(cfPWV) on the risk of ASCVD, the primary outcome. HRs adjusted for age, sex, SBP, HDLC, total cholesterol, smoking status, diabetes and antihypertensive medications. Pooled HR is estimated from a random effects meta-analysis, weighted by the number of events. N=15 987. ACCT, anglo-cardiff collaboration trial; ASCVD, atherosclerotic cardiovascular disease; BLSA, Baltimore Longitudinal Study of Ageing; CaPS, Caerphilly Prospective Study; cfPWV, carotid-femoral pulse wave velocity; DanMONICA, Danish-Multinational MONitoring of trends and determinants in Cardiovascular disease; EDIVA, The Estudo de DIstensibilidade VAscular project; Health ABC, The Health, Aging and Body Composition Study; HDLC, high-density lipoprotein cholesterol; NSHD, National Survey of Health and Development; SBP, systolic blood pressure.

Table 1 shows the HRs for each model, for the primary (ASCVD) and exploratory outcomes. After accounting for traditional CV risk factors (age, sex, SBP, high-density lipoprotein cholesterol (HDLC), total cholesterol, diabetes status at baseline, smoking status at baseline and antihypertensive medications at baseline), a 1-SD higher log_e_(cfPWV) was associated with a 21% (95% CI 8% to 36%) higher risk of ASCVD events (figure 1) and a 20% (95% CI 10% to 31%) greater risk of CV events (online supplemental figure S3). The HRs were similar across different types of CV events, but marginally lower for all-cause mortality (HR 1.13 (95% CI 1.08 to 1.18)). Funnel plots and Egger’s tests showed no evidence of small-study effects or selection bias. Martingale residual plots suggested that the association between standardised log_e_(cfPWV) and outcomes was approximately linear and Schoenfeld residuals supported the proportional hazards assumption (online supplemental figures S4–S7).

For the primary analyses, there was evidence of effect modification by smoking status (p<0.001). In separate analyses, a 1-SD higher cfPWV was associated with a higher risk of ASCVD in smokers compared with non-smokers. There was no evidence of effect modification by other variables (online supplemental figure S8). Subgroup analyses for all CV events were similar; however, there was also evidence of effect modification by BP category (p=0.008): a 1-SD higher cfPWV was associated with a higher risk of CVD in individuals with BP 140–159 mm Hg/90–99 mm Hg compared with individuals with BP 120–139 mm Hg/80–89 mm Hg, respectively (online supplemental figure S8). A study-level subgroup analysis showed that there was no significant difference between studies that had <10 years of follow-up compared with those with ≥10 years of follow-up (online supplemental figure S10). Similar results were generally obtained from sensitivity analyses (online supplemental tables S9–S11). A 1 m/s higher cfPWV (weighted mean and pooled SD were 9.70 m/s and 2.58, respectively) was associated with a 7.4% higher risk of ASCVD events and a 7.0% higher risk of CV events for a non-smoking, 64-year-old male without diabetes, not on BP medication, with an SBP of 135 mm Hg, a total cholesterol of 5.3 mmol/L and a HDLC of 1.4 mmol/L.

As there was some evidence of effect modification by stage of hypertension for all CV events, we performed a categorical analysis for ASCVD and all CV events based on clinical BP thresholds and the approximate median of cfPWV (~9 m/s). Online supplemental table S12 shows the summary statistics by category of hypertension and cfPWV. Among individuals with BP 120–139 mm Hg/80–89 mm Hg, the risk of ASCVD was significantly greater in those with a cfPWV >9 m/s (HR 1.44 (95% CI 1.27 to 1.64) (p<0.001)) compared with ≤9 m/s (HR 1.00 (95% CI 0.88 to 1.17)). Similarly, in individuals with BP 140–159 mm Hg/90–99 mm Hg, the risk of ASCVD was greater in those with a cfPWV >9 m/s, compared with those ≤9 m/s (HR 1.59 (95% CI 1.39 to 1.80) and HR 1.12 (95% CI 0.89 to 1.41), respectively). Conversely, the risk was relatively similar across categories of BP for individuals with either low (≤9 m/s) or high (>9 m/s) cfPWV (table 2). Results were comparable for all CV events (online supplemental table S13).

**Table 2:** Adjusted* HRs† for stage of hypertension and dichotomous cfPWV for ASCVD (n=12 516‡)

	BP: 120–139 mm Hg/80–89 mm Hg	BP: 140–159 mm Hg/90–99 mm Hg
Low cfPWV (≤9 m/s)	Ref. 1.00 (0.885 to 1.17)	1.1 (0.89 to 1.41), p=0.321
High cfPWV (>9 m/s)	1.44 (1.27 to 1.64), p<0.001	1.59 (1.39, 1.80), p<0.001

* Not adjusting for continuous cfPWV or SBP, HRs for categorical cfPWV and hypertensive status after adjusting for age, sex, smoking status, total cholesterol, HDL, antihypertensive medications and diabetes status.

† Floating HRs with prehypertension and low cfPWV as the reference group.

‡ NSHD and Rotterdam Study were not included as no IPD were available.

.ASCVD, atherosclerotic cardiovascular disease; BP, blood pressure; cfPWV, carotid-femoral pulse wave velocity; HDL, high-density lipoprotein; NSHD, National Survey of Health and Development; SBP, systolic blood pressure.

The addition of cfPWV to a risk model with traditional CV risk factors improved model discrimination (C-index change 0.0048 (95% CI 0.0002 to 0.0094), p=0.019 and C-index change 0.0034 (95% CI 0.0006 to 0.0062), p=0.018) for ASCVD and CVD, respectively) (table 3). There was also a significant improvement in discrimination of the models when predicting non-fatal CV events and all CHD events; however, the addition of cfPWV measurements to established CV risk factors did not improve all-cause mortality prediction.

**Table 3:** Measures of the model's ability to discriminate between events and non-events after the addition of continuous log_e_(cfPWV)

Endpoint	N	Events	C-index (95% CI) (CV risk factors*)	C-index (95% CI) (CV risk factors*+log_e_(cfPWV))	Difference (95% CI)	P value
Primary outcome						
ASCVD	15 987	1182	0.7118 (0.6973 to 0.7263)	0.7166 (0.7022 to 0.7311)	0.0048 (0.0002 to 0.0094)	0.041
Exploratory outcomes						
All CV events	15 987	1796	0.7057 (0.6938 to 0.7176)	0.7091 (0.6972 to 0.7210)	0.0034 (0.0006 to 0.0062)	0.018
Fatal CV events†	13 903	403	0.7618 (0.7391 to 0.7845)	0.7652 (0.7426 to 0.7877)	0.0033 (-0.0033 to 0.0100)	0.326
Non-fatal CV events‡	12 980	1485	0.7029 (0.6898 to 0.7159)	0.7061 (0.6931 to 0.7191)	0.0033 (0.0003 to 0.0062)	0.028
All CHD	15 550	1091	0.6972 (0.6817 to 0.7128)	0.7011 (0.6856 to 0.7166)	0.0039 (0.0003 to 0.0074)	0.033
All stroke events	15 987	692	0.7135 (0.6944 to 0.7326)	0.7193 (0.7003 to 0.7383)	0.0058 (-0.0013 to 0.0129)	0.109
All-cause mortality†	14 584	2867	0.7351 (0.7255 to 0.7447)	0.7366 (0.7270 to 0.7461)	0.0015 (-0.0009 to 0.0038)	0.230

* Age, sex, SBP, total cholesterol, smoking status, HDLC, diabetes status and antihypertensive medications.

† EDIVA does not contribute to fatal events due to short follow-up.

‡ ACCT, BLSA and Kinmen do not have data of non-fatal events, and EDIVA does not contribute to fatal events due to short follow-up.

ACCT, Anglo-Cardiff Collaboration Trial; ASCVD, atherosclerotic cardiovascular disease; BLSA, Baltimore Longitudinal Study of Ageing ; cfPWV, carotid-femoral pulse wave velocity; CHD, coronary heart disease; CV, cardiovascular; EDIVA, The Estudo de DIstensibilidade VAscular project; HDLC, high-density lipoprotein cholesterol; SBP, systolic blood pressure.

The corresponding integrated discrimination improvement (IDI) showed an improvement with the addition of cfPWV to the models for both ASCVD and CVD (IDI 0.0047 (95% CI 0.0016 to 0.0078), p=0.003 and IDI 0.0047 (95% CI 0.0018 to 0.0077), p=0.001, respectively). However, the reclassification indices did not show evidence of an improvement despite showing net positive changes (online supplemental table S14).

### Novel cfPWV risk scores

Summary statistics for the studies included in the derivation of risk scores are shown in online supplemental table S15. The sex-specific novel risk equations showed good discrimination and accuracy when cfPWV was added to PCE and SCORE2/SCORE2-OP risk equations (online supplemental tables S16 and S17). After the exclusion of one study, which had a very small age range, the C-indices and Brier scores were modestly improved. Online supplemental tables S18 and S19 show the validated prospective net reclassification index (NRI) statistics comparing the novel risk models to the established PCEs and SCORE-2 risk equations. The novel risk models showed reasonable calibration (online supplemental figure S11).

### Public health modelling

**Figure 2:**
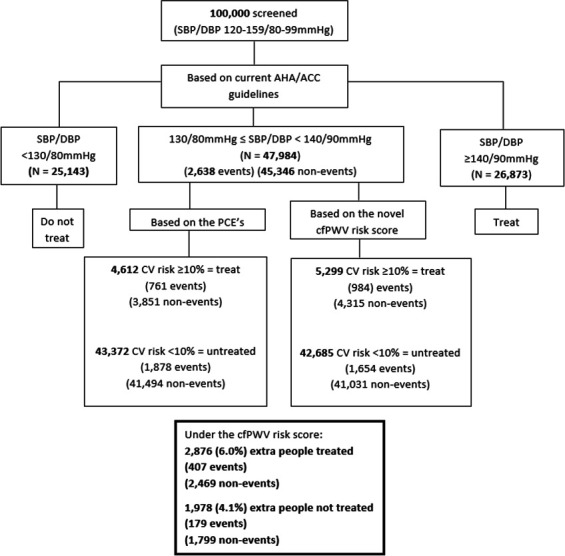
Flowchart showing the screening and treatment pathway for a hypothetical population of 100 000 individuals aged 40–79 years based on the PCEs and the novel cfPWV risk model for ASCVD, the primary outcome. ACC, American College of Cardiology; AHA, American Heart Association; ASCVD, atherosclerotic cardiovascular disease; cfPWV, carotid-femoral pulse wave velocity; CV, cardiovascular; DBP, diastolic blood pressure; PCE, pooled cohort equations; SBP, systolic blood pressure.

Under current US guidelines, individuals with BP<130/80 mm Hg are ineligible for antihypertensive treatment, while those with BP≥140/90 mm Hg qualify. Those in between require treatment only if their 10-year ASCVD risk is ≥10%. In our hypothetical population, 47 984 individuals fell into this middle category and underwent full risk screening (figure 2). Standard risk models (PCEs) classified 4612 for treatment, with 761 (16.5%) expected to develop ASCVD within 10 years, while 43 372 were deemed ineligible, with 1878 (4.3%) expected ASCVD cases. Adding cfPWV to risk models reclassified 2876 (6.0%) of the untreated group as eligible for treatment, of whom 407 (14.2%) were expected to develop ASCVD within 10 years.

Assuming a 20% reduction in CVD risk with treatment, using cfPWV to guide antihypertensive initiation could prevent 81 additional events over 10 years. Among those not eligible for antihypertensive treatment under current guidelines, 535 would need to be screened to prevent one additional event, with 32 (6.0%) receiving treatment, reducing the overall event rate by ~3.1% (table 4). For comparison, a public health analysis assessed the impact of treating the same number of additional individuals based solely on age. Treating the eldest instead would prevent 122 additional events but reduce the overall event rate by only ~0.8%. Repeating the clinical utility analyses using ESC guidelines (SCORE2 and SCORE2-OP) yielded similar results (online supplemental figures S12 and S13). cfPWV measurements could prevent 154 and 176 additional ASCVD events per 100 000 individuals, reducing event rates by ~3.2% and ~2.7%, using the SCORE2 and SCORE2-OP models, respectively (table 4). Online supplemental tables S20–S25 show the age-specific numbers in each hypothetical population, as well as the sex-specific proportions and numbers of individuals treated and untreated under each set of guidelines, with the currently used risk model and the novel cfPWV risk model.

**Table 4:** Summary of public health modelling measures if individuals ineligible for treatment under the established guidelines were rescreened with cfPWV measurements

		Model compared with cfPWV equation (age range (years))		
		PCE (40–79)*	SCORE2/ SCORE2-OP† (40–79)‡	SCORE2 (40–69)§
Number screened (per100 000)		47 984	94 159	92 812
Additional treated (per screened)	Total	2876 (6.0%)	9872 (10.5%)	11 039 (11.9%)
	Events	407 (14.2%)	879 (8.9%)	768 (7.0%)
NNS to prevent one additional event		535	375	480
NNT to prevent one additional event		32	39	57
Additional events prevented (cfPWV)		407 (3.1%)	879 (2.7%)	768 (3.2%)
Additional events prevented (age)		122 (0.8%)	270 (0.8%)	231 (1.0%)

Event incidence and subgroup proportions were based on data from the Whitehall II, DanMONICA, Health ABC and the Framingham cohorts (n=7019). Of the 92 812 individuals screened, the addition of cfPWV to risk models reclassified 11 039 (11.9%) as eligible for treatment, 768 (7.0%) of whom would be expected to have an ASCVD event within 10 years. Of those who were deemed ineligible for treatment under the current guidelines, 603 individuals would need to be screened to prevent one additional event, of whom 72 would be treated, and the overall event rate would be reduced by approximately 3.2%.

* Based on the AHA/ACC guidelines in the USA, individuals with an SBP/DBP<130/80 mm Hg were not eligible for antihypertensive treatment, and individuals with an SBP/DBP≥140/90 mm Hg were eligible for treatment. Individuals in the middle-risk group were eligible for treatment only if their predicted 10-year ASCVD risk was ≥10%. Of the 47 984 individuals screened, the addition of cfPWV to risk models reclassified 2876 (6.0%) as eligible for treatment, 407 (14.2%) of whom would be expected to have an ASCVD event within 10 years. Of those who were deemed ineligible for treatment under the current guidelines, 535 individuals would need to be screened to prevent one additional event, of whom 32 would be treated, and the overall event rate would be reduced by approximately 3.1%.

† Based on the ESC guidelines, individuals with normal/high-normal BP (SBP/DBP: 120–139 mm Hg/80–89 mm Hg) are screened if they are aged over 40 years and male, aged over 50 years and female or if they have any CV risk factors. Of the individuals who are screened, treatment initiation is guided by BP and age-specific thresholds of 10-year predicted risk. For those with grade 1 hypertension, treatment is initiated if their 10-year predicted risk is high or very high. For those with normal/high-normal BP, treatment is initiated if their predicted risk is very high. The SCORE2 model was used for individuals aged≥70 years to allow direct comparisons to the derived novel risk score. Of the 94,159 individuals screened, the addition of cfPWV to risk models reclassified 9872 (10.5%) as eligible for treatment, 879 (8.9%) of whom would be expected to have an ASCVD event within 10 years. Of those who were deemed ineligible for treatment under the current guidelines, 375 individuals would need to be screened to prevent one additional event, of whom 39 would be treated, and the overall event rate would be reduced by approximately 2.7%.

‡ As guided by the ESC, the SCORE2-OP model was used for individuals aged≥70 years and compared with the novel European ASCVD risk model.

§ The ESC recommends that the SCORE2 equation be used for individuals aged 40–69 years.

AHA/ACC, American Heart Association/American College of Cardiology; ASCVD, atherosclerotic cardiovascular disease; cfPWV, carotid-femoral pulse wave velocity; CV, cardiovascular; ESC, European Society of Cardiology; NNS, number needed to screen; NNT, number needed to treat; PCE, pooled cohort equations; SBP/DBP, systolic blood pressure/diastolic blood pressure; SCORE2, Systematic COronary Risk Evaluation; SCORE2-OP, Systematic COronary Risk Evaluation (Older Persons).

Additionally, using novel cfPWV models instead of established models lowered the number needed to screen to prevent one event by up to 72 individuals (online supplemental methods 4, table S26).

## Discussion

Our main finding was that cfPWV is an independent predictor of CV outcomes in individuals with BP 120–159 mm Hg/80–99 mm Hg, even after accounting for established CV risk factors. The addition of cfPWV to established risk factors improved risk prediction in this moderate BP group. In a hypothetical population of 100 000 individuals, assessing cfPWV in individuals not currently treated under established risk models but who are eligible for full risk screening could reduce the risk of CV outcomes by approximately 3%. While this reduction is modest, its value depends on further evidence regarding clinical acceptability, real-world implementation and cost-effectiveness.

As expected, adjusting for CV predictors, notably age, attenuated cfPWV HRs, whereas adjusting for key confounders of cfPWV (sex, mean arterial pressure and heart rate) had minimal impact. cfPWV’s association with ASCVD varied by smoking status, and its association with CVD (ASCVD+non-fatal CHD+heart failure) varied by both smoking and BP category. Current smokers had a higher ASCVD and CVD risk, and individuals with BP 140–159 mm Hg/90–99 mm Hg faced greater CVD risk than those with BP 120–139 mm Hg/80–89 mm Hg with increasing cfPWV. Dichotomising BP and cfPWV, we showed that individuals in either BP group: 120–139 mm Hg/80–89 mm Hg or BP 140–159 mm Hg/90–99 mm Hg who had a cfPWV greater than 9 m/s faced a higher CV risk compared with those with a cfPWV of 9 m/s or lower. Conversely, within each dichotomy of cfPWV, there did not appear to be a difference in CV risk comparing categories of BP, suggesting that in this moderate range of BP, aortic stiffness has more impact on CV risk than the level of BP per se.

We also evaluated the potential benefit of adding cfPWV to established risk calculators currently used in clinical practice, and guidelines in the USA (PCEs) and Europe (SCORE2/SCORE2-OP).^22 23 29^ The validated novel risk models demonstrated reasonable discrimination and calibration and improved risk stratification for treatment decisions. Integrating cfPWV screening into current guidelines could lead to approximately 14% and 9% more individuals with moderate BP being treated in Europe and the USA, respectively. This could reduce the risk of ASCVD events by approximately 3% compared with current practice, potentially preventing over 176 ASCVD events in Europe and 81 events in the USA per 100 000 individuals.

Although the addition of cfPWV to traditional risk models significantly improved discrimination, the gains were modest and may have limited clinical relevance. We reported the NRI due to its clinical interpretability and widespread use in risk prediction research; however, it did not reach statistical significance. In contrast, the IDI indicated improved sensitivity without increasing false positives. Additionally, population-level modelling suggested a small but tangible improvement in clinical utility when cfPWV was used to guide treatment decisions. Taken together, these findings raise important questions about whether incorporating cfPWV into risk assessment could influence clinical decision thresholds, particularly in individuals with moderate CV risk, based on BP.

Integrating novel cfPWV risk scores into clinical practice through a two-stage screening programme offers advantages, such as maintaining treatment for all individuals and reducing the number of cfPWV measurements compared with replacing current risk models entirely. However, a cost-benefit analysis is crucial to evaluate feasibility. A limitation of two-stage screening is that it will always prevent more events, and this could be achieved via random selection or another screening method rather than with additional cfPWV measurements. We have assessed and confirmed that cfPWV specifically selects high-risk individuals better than purely treating the same additional number of individuals based on age. Alternatively, if treating more individuals isn’t feasible, replacing current risk scores with cfPWV risk scores could be considered, though it would involve screening everyone with cfPWV. Another option is to measure cfPWV only in individuals with predicted CV risk between 5% and 10%, potentially halving the number of required measurements.

Our findings have particular relevance for individuals with moderate BP (120–159 mm Hg/80–99 mm Hg), a group for whom treatment decisions often rely on additional risk stratification. Recent ESC guidelines for the management of elevated BP and hypertension have listed cfPWV as a secondary or optional marker, particularly in individuals where treatment remains uncertain.^11^ Notably, the ESC guidelines consider elevated cfPWV as evidence of hypertension-mediated organ damage, which may influence the decision to initiate pharmacological therapy. In our population modelling, the use of cfPWV to guide treatment in this group led to a modest reduction in ASCVD event rates and improved targeting of therapy. These findings suggest that cfPWV could enhance decision-making in clinical grey zones, helping to identify patients more likely to benefit from early intervention and potentially supporting shared decision-making by providing a clearer picture of individual risk.

Our findings align with previous meta-analyses, confirming that cfPWV independently predicts CV events beyond traditional risk factors in both general and clinical populations.^13 47 48^ Recent data from the Whitehall II cohort further support this association, particularly in older individuals.^46^ Our study includes several large cohorts not included in previous meta-analyses (Whitehall II, Framingham cohorts and National Survey of Health and Development) and focuses on the clinically relevant endpoint used in the current USA and European hypertension guidelines.^10 29^ Specifically, our focus on individuals with the highest potential for benefit demonstrates that cfPWV enhances risk prediction in this clinically relevant group of individuals with moderate BP, who typically span the threshold for antihypertensive treatment initiation. While the improvement is small, incorporating cfPWV refines CV risk prediction and could impact treatment decisions.

Our study does have some limitations. The observational design of this study limits these findings; we cannot claim any causal associations. However, we have incorporated large sample sizes with careful adjustment to reduce any bias from this as much as possible. While we adjusted for baseline antihypertensive use, some individuals may have started treatment during follow-up, potentially affecting arterial stiffness and underestimating HRs. Treatment changes or non-compliance could also influence results, though individuals were unlikely to discontinue medication. Xu *et al* found that incorporating statin effects had little impact on treatment decisions, likely similar for antihypertensives.^49^ Our focus on moderate BP limited event numbers, especially when dichotomising BP and cfPWV, warranted cautious interpretation. Although ASCVD was the primary outcome, other CV outcomes, including stroke and CHD, were considered as exploratory outcomes, and as no adjustment for multiplicity was accounted for, results involving exploratory outcomes and subgroup analyses should be interpreted correspondingly. Though statistically significant, C-index improvements were marginal, raising questions about clinical relevance. Follow-up durations varied, but we found no effect modification. Some variables (eg, history of myocardial infarction (MI), chronic kidney disease and erectile dysfunction) were unavailable, limiting comparisons with QRISK3 (a CV risk prediction tool recommended in the UK^50^); however, SCORE2/SCORE2-OP equations are also relevant for the UK. Additionally, most included studies involved individuals of white ethnicity who may over-represent Western populations, so generalising findings to other ethnic groups and low/middle-income populations requires caution. A final limitation is the lack of data on specific classes of antihypertensive medications. Although the Strategy for Preventing cardiovascular and renal events based on ARTErial stiffness (SPARTE) study showed no differential effects of drug classes on cfPWV,^51^ a meta-analysis looking at a broader medication range showed some evidence that certain drug classes may have differential effects on cfPWV beyond their BP-lowering properties;^52^ the majority of cfPWV reduction is thought to be driven by reductions in BP itself.^53^ Without class-specific treatment information, we were unable to account for potential variation in cfPWV response due to medication type.

In summary, large artery stiffness, as assessed by cfPWV, is an independent risk factor for ASCVD and other CV events. The current study showed that cfPWV is an independent risk factor beyond established CV risk factors in individuals with moderate BP. Indeed, the measurement is non-invasive, inexpensive and adds value to already established CV risk models. However, cost-effectiveness analyses and clinician willingness assessments are needed before widespread adoption into clinical practice. Nevertheless, inclusion of novel cfPWV risk models into clinical guidelines could improve risk stratification, act as an ‘early warning marker’ and may, ultimately, reduce CV events.

## Supplementary material

10.1136/bmjopen-2025-101368 online supplemental file 1

10.1136/bmjopen-2025-101368 online supplemental file 2

10.1136/bmjopen-2025-101368 online supplemental file 3

## Data Availability

Data are available upon reasonable request.
