# Supplementary material for: Association between carotid-femoral pulse wave velocity and cardiovascular disease in individuals with moderate blood pressure: a systematic review and individual participant meta-analysis
Source: BMJ Open. 2025 Dec 15;15(12):e101368. doi: 10.1136/bmjopen-2025-101368 (PMC12706244; doi:10.1136/bmjopen-2025-101368)
Supplement: online supplemental file 1 [file bmjopen-15-12-s001.pdf]

**Association between carotid-Femoral Pulse Wave Velocity and cardiovascular disease in individuals with moderate blood pressure: a systematic review and individual participant meta-analysis**

# SUPPLEMENT

## Additional Statistical methods

## Supplementary methods 1: Primary analyses

Anonymised individual-level data were requested for each study including cardiovascular risk factors and outcomes. Participants with BP 120-139/80-89mmHg or BP 140-159/90-99mmHg were included (referred to as "individuals with moderate BP" throughout these analyses). Individuals with a history of CVD were excluded from the analyses, as well as all individuals with BP <120/80mmHg and those with high BP or more severe ≥160/100mmHg.

Cardiovascular risk factors included: cfPWV, age, sex, SBP, body mass index (BMI), smoking status, diabetes status, antihypertensive medications, mean arterial pressure (MAP), heart rate, total cholesterol, high density lipoprotein (HDL) cholesterol and ethnicity.

No outcomes or follow up times were imputed since there were <0.001% missing. Any individuals with outcomes and follow-up times missing were censored at their examination date and hence will not contribute to any analyses. Studies with data on at least one endpoint will remain in the analyses and will contribute to their respective outcomes only. All individuals with event outcome missing were not included in the analyses, except where an individual has a censor date, it was assumed that they had no occurrence of a fatal event at the time they were censored.

The primary outcome was ASCVD and was defined in accordance with the ACC/AHA (which was equivalent to the CVD endpoint used by the ESC for SCORE2/SCORE2-OP), including CHD death, nonfatal MI and fatal or nonfatal stroke, where this level of outcome data was unavailable ASCVD endpoints included CHD death and fatal or nonfatal Stroke, so there may be a small underestimation of ASCVD events (1–3). Not all studies had each of the outcomes recorded, so not all studies contributed to analyses for every endpoint. ASCVD and composite CV events included all studies where either fatal CV or non-fatal CV or where both events were recorded. This assumes that there is no heterogeneity in the effect of cfPWV on fatal and non-fatal events and may underestimate the event rate. Sensitivity analyses were performed including only those studies with data for both fatal and non-fatal CV events.

For two studies where individual participant data was unable to leave the local sites, analyses were performed on the data at the local sites and summary statistics and relevant coefficients such as hazard ratios and standard errors were included in the meta-analyses.

Individuals with implausible values of covariates were excluded from the analyses, including upper limits of cfPWV > 25m/s, total cholesterol > 11mmol/L and HDL > 4mmol/L and lower limits: cfPWV < 3m/s and total cholesterol < 1mmol/L. In one study where diabetes at baseline was not pre-derived, diabetes status at baseline was derived from fasting serum glucose ≥ 7 mmol/L or > 2h post load serum glucose tolerance test level ≥ 11.11mmol/L. MAP was derived from SBP and diastolic blood pressure (DBP) where not already derived in the dataset (*MAP = DBP + 1/3(SBP-DBP)*).

Study-specific hazard ratios of cfPWV were pooled using a two-stage random effects meta-analysis to allow for heterogeneity between studies. The method of DerSimonian & Laird was used, with the estimate of heterogeneity being taken from the from the Mantel-Haenszel model. Forest plots show the individual study hazard ratios, 95% confidence intervals and the random-effects weightings of cfPWV on each outcome as well as the pooled hazard ratio.

For sensitivity analyses, missing IPD was imputed using the jomo package in R, implementing a random-effects imputation model to allow for study variation. The event outcome and Nelson-Aalen estimator of survival time were included as outcome variables. For the two studies where analyses were run at the local sites, study-specific, single level fixed-effects imputation was performed and the hazard ratios analysed using the imputed data,

estimating the covariance matrix from similar studies. Imputed study-specific hazard ratios were then pooled in a two-stage random effects meta-analysis. Subgroup analyses were performed within each study by fitting models separately within each pre-specified subgroup and the hazard ratios of cfPWV within each subgroup pooled using random-effects meta-analyses. Stratification by ethnicity was assessed by assessing changes in hazard ratios and proportional hazards assumptions. Ethnicity was categorised into white and non-white, it was not possible to stratify by further ethnic groups due to small numbers of events in stratum within some studies.

For each outcome, models adjusted for traditional CV risk factors with and without the addition of $\log_e$(cfPWV) were fitted to assess whether $\log_e$(cfPWV) added predictive value. To assess the prognostic ability of each risk prediction model, C-indices were calculated. Harrell's C-index, defined as the proportion of observations that the model can order correctly in terms of survival times, is a measure of the discrimination, the prognostic value of the risk model (4). Study specific C-index measure were calculated for models with and without $\log_e$(cfPWV) and pooled, weighted by the number of events within each study.

Reclassification measures including the prospective NRI (categorical) (5,6) and the integrated discrimination improvement (IDI) (7) were limited to studies that included individuals with more than 10 years follow-up and contributed to both fatal and non-fatal events. The IDI summarises the extent a new model increases risk in those who have events and decreases risk in those who do not have events and then averages risk across those with and without events. Further descriptions of these calculations are provided in supplementary methods 2. The movement of individuals between 10-year cardiovascular risk categories defined by established risk thresholds (low-risk ≤10%, moderate-risk: 10-20% and high risk: ≥20%) determined the categorical NRI reclassification thresholds).

## Supplementary methods 2: Derivation and evaluation of novel cfPWV risk scores

Two sex-specific risk scores were derived, with ASCVD as endpoints, allowing for direct comparisons to the recalibrated PCE's and the SCORE2/SCORE2-OP equations, respectively:

CfPWV risk model 1: PCE's (US)
CfPWV risk model 2: SCORE2/SCORE2-OP (Europe)

Individuals aged 40-79 without a history of CVD were included in the novel risk models and compared to re-calibrated established risk equations as described below. From the 11 studies used in the primary analyses, two studies did not provide IPD and two studies had a median follow-up time <10 years. Three of the remaining seven studies had 10-year survival probabilities very close to one: due to few events. Therefore, four studies remained in this analysis for the model derivation and validation: Whitehall II, DanMONICA, Health ABC and the Framingham study. Table S7 shows the summary statistics of these studies (shown underlined).

### Recalibrating established risk models

To accurately capture the event rates in the studies used in these analyses, established risk models were recalibrated to the data.

#### *Pooled Cohort Equations*

Sex-specific PCE's were re-calibrated to the individual participant data by updating the linear predictor with a correction factor (CF) calculated using the calibration in the large method (8). Recalibration by the calibration in the large method follows the following form:

$$S_0(t)\{\exp[\beta_1(x_1 - M_1) + \cdots + \beta_p(x_p - M_p) + CF]\},$$

where, the correction factor is calculated by:

$$CF = \ln\left\{\frac{\left[\frac{n}{N}\right]}{1-\left[\frac{n}{N}\right]} \bigg/ \frac{\frac{\frac{1}{N}\sum_1^N Risk_{PCE}}{100}}{1-\left(\frac{\frac{1}{N}\sum_1^N Risk_{PCE}}{100}\right)}\right\},$$

where, $N$ is the total number of individuals, $n$ is the total number of events from the population used for the model derivation and $Risk_{PCE}$ is the mean predicted risk when the original PCE's were fitted to the data (1).

All continuous variables were log-transformed and all interactions as in the original PCE's were included. The calibration in the large method for recalibration was used for the PCE's to allow the inclusion of the interaction terms in the risk model.

### *SCORE2/SCORE2-OP*

Sex-specific SCORE2/SCORE2-OP equations were fitted to our data and recalibrated to the 'low-risk' version of the model to predict 10-year ASCVD risk, under the assumption that the two cohorts from the US are comparable to low-risk cohorts from Europe.

## Evaluation

Performance measures including the C-statistic, brier score, calibration and categorical prospective net reclassification indices (NRI's) were calculated. Established thresholds were used to calculate the NRI's (low-risk ≤10%, moderate-risk: 10-20% and high risk: ≥20%) to compare the novel models to the thresholds used in the US (9). The age-specific risk categories as defined by the European Society of Cardiology (ESC) in the 2021 guidelines for CV risk were used when comparing the novel models to the SCORE2/SCORE2-OP models (10).

The prospective NRI was also calculated since this allows the inclusion of individuals who were censored prior to 10 years to remain in the calculation (5). The following shows the prospective form of the NRI when risk groups are categorised:

$$NRI_{prosp,cat} = \frac{P(event|up) - P(event)).P(up) + (P(event) - P(event|down)).P(down)}{P(event).(1 - P(event))}$$

This prospective NRI can be interpreted as a measure of event rate increase in individuals who are reclassified up and event rate decrease in those reclassified down.

The integrated discrimination index (IDI) is measure of the capacity of a new marker to predict an outcome, it is more sensitive than the NRI at identifying new risk factors and biomarkers for prognostic models (7). It can be estimated by:

$$IDI = (\bar{\hat{p}}_{new,events} - \bar{\hat{p}}_{old,events}) - (\bar{\hat{p}}_{new,nonevents} - \bar{\hat{p}}_{old,nonevents}),$$

where $\bar{\hat{p}}$ is an average of the estimated probabilities of all individuals who had an event (or who did not have an event) corresponding the novel (new) risk score and the original (old) risk score. A useful risk factor would lead to higher estimated risk of outcome for individuals who have an event, and lower estimated risk of outcome for those who do not have an event, subsequently corresponding to a larger IDI.

## Supplementary methods 3: Model validation

The regular bootstrap method of internal validation (Harrell's bias correction) was performed (11), taking 400 bootstrap samples to account for any optimism in the C-statistic, brier score, calibration and reclassification measures due to over-fitting when deriving the model (See Figure S14).

### Apparent estimates

The novel sex-specific cox proportional hazards cfPWV model was fitted on the original sample, stratified by study and the predicted 10-year risk calculated using the linear predictor and the baseline survival at 10-years from the original sample:

$$1 - (study\ specific\ 10y\ baseline\ survival)^{exp(linear\ predictor)}$$

The c-index and brier score were calculated, within each study, from the predicted risks.

To assess model calibration, deciles of predicted risk were calculated from the 10-year predicted risks, within each study, and compared to the deciles of observed risks. Observed risks were calculated by estimating the mean Kaplan-Meier survival estimate within each study, by decile of predicted risk. For each study and decile, calibration was calculated by: *mean(Predicted risk) – mean(Observed risk).*

### Bootstrap estimates

*B* bootstrap samples of size n, stratified by study, were taken, sampling the original sample with replacement, where n is the size of the original sample. The methods, as for the apparent estimate, were repeated to calculate the bootstrap estimates on each bootstrap sample, *b*, producing *B* bootstrap estimates. In this case, *B* = 400. In the case that there were no events within each study in a bootstrap sample, the performance measure for that bootstrap sample, *b*, equated to missing.

### Test estimates

The models fitted to each bootstrap sample, specifically the linear predictors, baseline survival function and means of the covariates, were used to predict the 10-year predicted risk in the apparent sample (test predicted risk), from which *b* estimates of each performance measure were computed. Calibration was calculated using the test predicted risks sample, and the observed risk calculated as in the apparent estimates for the original sample.

The Harrell's bias correction method of bootstrap-based correction was used to adjust the estimates for optimism ($\delta$):

$$\delta = \frac{1}{B}\sum_{b=1}^{B}\left(\hat{\theta}_{b,bs} - \hat{\theta}_{b,test}\right),$$

And the corrected performance measure ($\theta_{corr}$) was calculated by:

$$\hat{\theta}_{corr} \quad = \hat{\theta}_{app} - \delta$$

### Location-shift method to obtain confidence intervals

To save computational time, the location-shift method to obtain confidence intervals for the optimism corrected performance measures. The bootstrap samples were used to estimate 95% confidence estimates around each performance measure for the apparent estimate. The 95% confidence limits were shifted by the optimism for that particular performance measure (12). To allow confidence intervals to be computed for calibration

estimates, the difference between the observed and predicted 10-year risks were estimated for each decile of predicted risk within each bootstrap sample (13).

## Supplementary methods 4: Clinical utility analyses/Population health modelling

### Methods

Public health modelling was performed to assess the clinical implications of the novel cfPWV risk models for ASCVD, based on the current US and European guidelines. Reclassification was based on observed data from participants with complete information on conventional risk factors and cfPWV (7,019 individuals from four cohort studies (Whitehall II, DanMONICA, Health ABC and Framingham Heart Study).

Three primary clinical utility analyses were performed:

1) Comparing the novel cfPWV model using the covariates and interactions as in the pooled cohort equations (PCE's) to the recalibrated PCE's under the ACC/AHA guidelines in individuals aged 40-79 years,
2) Comparing the novel cfPWV model using the covariates and interactions as in the SCORE2 risk equations to the established SCORE2 risk model recalibrated to low risk countries, under the ESC guidelines in individuals aged 40-69 years,
3) Comparing the novel cfPWV models using the covariates and interactions as in the SCORE2 / SCORE2-OP risk equations to the established SCORE2 / SCORE2-OP models recalibrated to low risk countries, under the ESC guidelines in individuals aged 40-79 years.

For sensitivity analyses, for each of the above, a public health analysis was also performed, looking at situations where the established risk models were replaced with the novel cfPWV risk models, instead of used in addition to.

It was assumed that treatment with antihypertensive medications reduced ASCVD risk by 20% (14,15).

### 1) ACC/AHA Guidelines for hypertension treatment initiation - US

Based on the 2019 ACC/AHA hypertension guidelines, individuals with a BP >140/90mmHg are treated with antihypertensive medications. Individuals with a BP <130/80mmHg are not treated. The middle risk individuals with BP 130-140/80-90mmHg are treated if their 10-year ASCVD predicted risk >10% (based on the PCE's).

We modelled a hypothetical population of 100,000 middle-risk individuals based on the standard US population (2000). The standard US population was equivalent for males and females and categorised by 5-year age-groups. Since the focus is on individuals aged 40-79, the standard population (millions) for these age groups were factored up so the whole hypothetical population consisted of one million individuals aged 40-79 years. This was then scaled down by a factor of 10 to establish a hypothetical population of 100,000 individuals aged 40-79 years (Table S20). The incidence of events and proportions of cases and non-cases treated has been calculated separately in each sex (Table S21).

The number of events was estimated by calculating the sex-specific cumulative incidence of ASCVD events within each 10-year age-group, based on the exponential survival distribution. The cumulative incidence proportion was then multiplied by the hypothetical number of individuals in each age-group based on the US standard population. The non-events within each sex were then calculated by subtracting the number of events by the total number of males and females. It has been assumed that the incidence of ASCVD in the general US population was the same as the sex and age-specific cumulative incidence rates of ASCVD in the data.

$$\textbf{Cumulative incidence}_{ij} = 1 - \exp(-IR_{ij} * T), \quad [1]$$

$$\textbf{Incidence rate }(\textbf{IR})_{ij} = \frac{Total\ number\ of\ events\ within\ 10\ year_{ij}}{Total\ follow\ up\ time\ (years)_{ij}}, \quad [2]$$

$where\ IR = incidence\ rate$, *T = 10 years, i=age group, j=sex.*

10-year ASCVD risk was predicted using the PCE's and the novel cfPWV risk model. Risk was categorised into 'treat' or 'not treat' based on whether the risk was ≥10% or <10%. Proportions treated and untreated under each risk equation were calculated and multiplied by the hypothetical middle-risk population. To estimate the number of individuals who were treated if they had an event under each risk model, the proportion of individuals with events within each sex and age-category who are treated was calculated. The number who were untreated under each model was then estimated by subtracting the number of treated events from the total number of events within each category.

$$\textbf{Cumulative incidence}_{ijkl} = 1 - \exp(-IR_{ijkl} * T), \quad [3]$$

$$\textbf{Incidence rate }(\textbf{IR})_{ijkl} = \frac{Total\ number\ of\ events\ within\ 10\ year_{ijkl}}{Total\ follow\ up\ time\ (years)_{ijkl}}, \quad [4]$$

where IR = incidence rate, T = 10 years, i = age group, j = sex, k = BP category and l = treated/model combination.

### 2) ESC guidelines for hypertension treatment initiation – Europe – SCORE2

Based on the 2021 ESC cardiovascular guidelines, individuals with BP: 120-139/80-89mmHg, defined as normal or high-normal blood pressure are screened if they are male aged over 40, female aged over 50 or if they have any CV risk factors. All individuals with grade-1 hypertension, defined as BP: 140-159/90-99mmHg are screened.

Treatment initiation is guided by age-specific 10-year ASCVD risk predictions (10). Individuals with normal or high-normal BP who are screened are treated if their predicted 10-year ASCVD risk is very high, individuals with grade-1 hypertension are treated if their predicted 10-year ASCVD risk is high or very high. The SCORE2 risk equation is used for individuals aged 40-69.

A hypothetical population of 100,000 individuals was derived based on the standard European population (2013). The European standard population was pooled across both sexes and categorised by 5-year age groups. Since the focus is on individuals aged 40-69, the standard population for these age groups were factored up so the whole hypothetical population of 40-69 year olds consisted of 100,000 individuals (Table S22).

Since our analyses are looking only at individuals with BP 120-160/80-100mmHg, we have assumed that the standard population distribution for Europe is applicable within this subgroup. Comparing proportions of age categories in the 'middle-risk' population to the whole population show approximately comparable proportions, so this approximately holds.

As above, we used 10-year age groups and also assumed that the proportion of males and females with normal/high-normal and grade-1 hypertension were approximately equal.

We allowed the proportion of males and females treated under each risk model to vary by sex (Table S23).

The cumulative incidence was calculated for each 10-year age category as in equation [1] and multiplied by the hypothetical number of individuals in each age group based on the European standard population to compute the hypothetical number of events within each age category.

10-year ASCVD risk was predicted using the SCORE2 and the novel cfPWV risk equations. Risk was categorised into treat or not treat based on stage of hypertension, presence of CV risk factors and age-based risk thresholds. Proportions treated and untreated under each risk equation were derived and multiplied by the middle risk hypothetical population within normal/high-normal BP and grade-1 hypertension. To calculate the number of events under each model classification, the age and sex-specific cumulative incidence was calculated for each treatment/model combination within each BP (screening) category as in equation [4].

### 3) ESC guideline for hypertension treatment initiation – Europe – SCORE2/SCORE2-OP

The above analyses in 2) were repeated expanding the population to include those aged 40-79 years. As above, treatment initiation is guided by age-specific 10-year ASCVD risk predictions: Individuals with normal or high-normal BP who are screened are treated if their predicted 10-year ASCVD risk is very high, individuals with grade-1 hypertension are treated if their predicted 10-year ASCVD risk is high or very high. The SCORE2 risk equation is used for individuals aged 40-69 and the SCORE2-OP risk equation is used for individuals aged 70+.

Since the focus is on individuals aged 40-79, the standard population for these age groups were factored up so the whole hypothetical population of 40-79 year olds consisted of 100,000 individuals (Table S24).

As above, it was assumed that the standard population distribution for Europe (40-79 years) is applicable within the subgroup of individuals with BP 120-160/80-100mmHg.

Sex specific models were fitted to estimate the proportion of individuals treated or not treated under each risk model, by age-group (Table S25).

**Reclassification**

To calculate the reclassification statistics, the risk categories (treat or don't treat) for the established models and the novel cfPWV models were tabulated by event status (event or no event). A reclassification variable was generated categorising correct/incorrect movement: correct up, correct down, wrong up, wrong down and unchanged. The relevant proportions of individuals in each category within each age-group were calculated. The number of individuals in each age category from the hypothetical population were multiplied by the proportion reclassified to each reclassification category. Then the total number of correct, incorrect and unchanged individuals from the hypothetical population was calculated.

The total number reclassified as 'correct up' or 'wrong up' contributed to the additional number treated under the cfPWV risk model, with 'correct up' numbers of events and 'wrong up' numbers of non-events. The total number reclassified as 'correct down' or 'wrong down' contributed to the additional number of individuals who would not have been treated, where under established guidelines they would have been, with 'wrong down' events and 'correct down' numbers of non-events. The number 'unchanged' applies where individuals are classified to the same risk category under both models.

**Number needed to treat/screen (NNS/NNT) to prevent one event**

Since our analyses are looking only at individuals with BP 120-160/80-100mmHg, we have assumed that the relevant standard population distributions are applicable within this subgroup of the population.

To calculate the number needed to treat (NNT) to prevent one event in ten years under each set of guidelines and risk model, we assumed 20% treatment effectiveness (14,15). Assuming a 20% treatment effectiveness, *a (0.2 X number of treated events)* events could be prevented in 10 years. Over the course of 10 years, one event could be prevented for every b people screened (number screened / *a*), as a result of c people starting antihypertensive therapy (*number of treated individuals* X *b*).

To calculate the number needed to screen, after cfPWV measurement, to prevent one additional event in 10 years compared to the current guidelines, assuming those not treated under current guidelines were re-screened with additional cfPWV measurements, the number of treated events and proportion of treated individuals are replaced with the additional number of treated events and proportion of treated individuals after reclassification using additional screening with the cfPWV risk model, respectively.

**Comparison to treating the equivalent number of additional individuals based on age**

For each of the three public health modelling analyses, within the subset of individuals who, based on current guidelines and screening with the established risk models, would not be treated, the equivalent number of individuals who were additionally treated with extra cfPWV screening would be treated based on age (eldest). Within this subgroup, the proportion of individuals having an event if they were treated based on age was calculated, within each age group. This was then multiplied by the number in the hypothetical population in the respective age category within this subgroup.

## References


1. Goff DC, Lloyd-Jones DM, Bennett G, Coady S, D'Agostino RB, Gibbons R, et al. 2013 ACC/AHA Guideline on the Assessment of Cardiovascular Risk. Circulation [Internet]. 2014 Jun 24;129(25_suppl_2):S49–73. Available from: https://doi.org/10.1161/01.cir.0000437741.48606.98

2. SCORE2 risk prediction algorithms: new models to estimate 10-year risk of cardiovascular disease in Europe. Eur Heart J [Internet]. 2021 Jul 1;42(25):2439–54. Available from: https://doi.org/10.1093/eurheartj/ehab309

3. SCORE2-OP working group and ESC Cardiovascular risk collaboration. SCORE2-OP risk prediction algorithms: estimating incident cardiovascular event risk in older persons in four geographical risk regions. Eur Hear J. 2021;42(25):2455–67.

4. Harrell FE Jr, Califf RM, Pryor DB, Lee KL RR. Evaluating the yield of medical tests. JAMA. 1982;247(18):2543–6.

5. Pennells L, Kaptoge S, White IR, Thompson SG, Wood AM, Collaboration ERF. Assessing risk prediction models using individual participant data from multiple studies. Am J Epidemiol. 2013/12/22. 2014 Mar;179(5):621–32.

6. Pencina MJ, D'Agostino RB Sr SE. Extensions of net reclassification improvement calculations to measure usefulness of new biomarkers. Stat Med. 2011;30(1):11–21.

7. Pencina MJ, D'Agostino RB Sr, D'Agostino RB Jr VR. Evaluating the added predictive ability of a new marker: from area under the ROC curve to reclassification and beyond. Stat Med. 2008;27(2):157–72.

8. Janssen KJM, Moons KGM, Kalkman CJ, Grobbee DE, Vergouwe Y. Updating

methods improved the performance of a clinical prediction model in new patients. J Clin Epidemiol [Internet]. 2008;61(1):76–86. Available from: https://www.sciencedirect.com/science/article/pii/S0895435607002132

9. Arnett DK, Blumenthal RS, Albert MA, Buroker AB, Goldberger ZD, Hahn EJ, et al. 2019 ACC/AHA Guideline on the Primary Prevention of Cardiovascular Disease: A Report of the American College of Cardiology/American Heart Association Task Force on Clinical Practice Guidelines. Vol. 140, Circulation. 2019. 596–646 p.

10. Visseren FLJ, Mach F, Smulders YM, Carballo D, Koskinas KC, Bäck M, et al. 2021 ESC Guidelines on cardiovascular disease prevention in clinical practice. Eur Heart J [Internet]. 2021 Sep 7;42(34):3227–337. Available from: https://doi.org/10.1093/eurheartj/ehab484

11. Harrell FE Jr, Lee KL MD. Multivariable Prognostic Models: Issues in Developing Models, Evaluating Assumptions and Adequacy, and Measuring and Reducing Errors. Stat Med. 1996;15:361–87.

12. Noma H, Shinozaki T, Iba K, Teramukai S, Furukawa TA. Confidence intervals of prediction accuracy measures for multivariable prediction models based on the bootstrap-based optimism correction methods. arXiv. 2020;

13. Wongvibulsin S, Wu KC, Zeger SL. Clinical risk prediction with random forests for survival, longitudinal, and multivariate (RF-SLAM) data analysis. BMC Med Res Methodol. 2019 Dec;20(1):1.

14. Blood Pressure Lowering Treatment Trialists' Collaboration. Pharmacological blood pressure lowering for primary and secondary prevention of cardiovascular disease across different levels of blood pressure: an individual participant-level data meta-analysi. Lancet. 2021;397(10288):1884.

15. Wu J, Kraja AT, Oberman A, Lewis CE, Ellison RC, Arnett DK, et al. A Summary of the Effects of Antihypertensive Medications on Measured Blood Pressure. Am J Hypertens. 2005 Jul;18(7):935–42.

16. Mitchell GF, Hwang SJ, Vasan RS, Larson MG, Pencina MJ, Hamburg NM, et al. Arterial stiffness and cardiovascular events: The framingham heart study. Circulation [Internet]. 2010;121(4):505–11. Available from: http://ovidsp.ovid.com/ovidweb.cgi?T=JS&PAGE=reference&D=med6&NEWS=N&AN=20083680

17. Shokawa T, Imazu M, Yamamoto H, Toyofuku M, Tasaki N, Okimoto T, et al. Pulse wave velocity predicts cardiovascular mortality: Findings from the Hawaii-Los Angeles-Hiroshima study. Circ J [Internet]. 2005;69(3):259–64. Available from: http://ovidsp.ovid.com/ovidweb.cgi?T=JS&PAGE=reference&D=med5&NEWS=N&AN=15731528

18. Kuh D, Pierce M, Adams J, Deanfield J, Ekelund U, Friberg P, et al. Cohort profile: updating the cohort profile for the MRC National Survey of Health and Development: a new clinic-based data collection for ageing research. Int J Epidemiol. 2011 Feb;40(1):e1–9.

19. AlGhatrif M, Strait JB, Morrell CH, Canepa M, Wright J, Elango P, et al. Longitudinal trajectories of arterial stiffness and the role of blood pressure: The Baltimore longitudinal study of aging. Hypertension [Internet]. 2013;62(5):934–41. Available from: http://ovidsp.ovid.com/ovidweb.cgi?T=JS&PAGE=reference&D=emed15&NEWS=N&AN=370115850

20. Ikram MA, Brusselle GGO, Murad SD, van Duijn CM, Franco OH, Goedegebure A, et al. The Rotterdam Study: 2018 update on objectives, design and main results. Eur J Epidemiol. 2017;32(9):807–50.

21. Valencia-Hernández CA, Lindbohm J V, Shipley MJ, Wilkinson IB, McEniery CM, Ahmadi-Abhari S, et al. Aortic pulse wave velocity as adjunct risk marker for assessing cardiovascular disease risk: prospective study. Hypertens (Dallas, Tex 1979). 2022 Apr;79(4):836–43.

22. Sutton-Tyrrell K, Najjar SS, Boudreau RM, Venkitachalam L, Kupelian V, Simonsick EM, et al. Elevated aortic pulse wave velocity, a marker of arterial stiffness, predicts cardiovascular events in well-functioning older adults. Circulation [Internet]. 2005;111(25):3384–90. Available from: http://ovidsp.ovid.com/ovidweb.cgi?T=JS&PAGE=reference&D=med5&NEWS=N&AN=15967850

23. Willum-Hansen T, Staessen JA, Torp-Pedersen C, Rasmussen S, Thijs L, Ibsen H, et al. Prognostic value of aortic pulse wave velocity as index of arterial stiffness in the general population. Circulation [Internet]. 2006;113(5):664–70. Available from: http://ovidsp.ovid.com/ovidweb.cgi?T=JS&PAGE=reference&D=med5&NEWS=N&AN=16461839

24. Mattace-Raso FUS, Van Der Cammen TJM, Hofman A, Van Popele NM, Bos ML, Schalekamp MADH, et al. Arterial stiffness and risk of coronary heart disease and stroke: The Rotterdam Study. Circulation [Internet]. 2006;113(5):657–63. Available from: http://ovidsp.ovid.com/ovidweb.cgi?T=JS&PAGE=reference&D=med5&NEWS=N&AN=16461838

25. Maldonado J, Pereira T, Polonia J, Silva JA, Morais J, Marques M, et al. Arterial stiffness predicts cardiovascular outcome in a low-to-moderate cardiovascular risk population: the EDIVA (Estudo de DIstensibilidade VAscular) project. J Hypertens [Internet]. 2011;29(4):669–75. Available from: http://ovidsp.ovid.com/ovidweb.cgi?T=JS&PAGE=reference&D=med7&NEWS=N&AN=21252699

26. Wang KL, Cheng HM, Sung SH, Chuang SY, Li CH, Spurgeon HA, et al. Wave reflection and arterial stiffness in the prediction of 15-year all-cause and cardiovascular mortalities: a community-based study. Hypertens (Dallas, Tex 1979) [Internet]. 2010;55(3):799–805. Available from: http://ovidsp.ovid.com/ovidweb.cgi?T=JS&PAGE=reference&D=med6&NEWS=N&AN=20065155

27. Johansen NB, Vistisen D, Brunner EJ, Tabak AG, Shipley MJ, Wilkinson IB, et al. Determinants of aortic stiffness: 16-year follow-up of the Whitehall II study. PLoS One [Internet]. 2012;7(5):e37165. Available from: http://ovidsp.ovid.com/ovidweb.cgi?T=JS&PAGE=reference&D=med7&NEWS=N&AN=22629363

28. McEniery CM, Yasmin, Maki-Petaja KM, McDonnell BJ, Munnery M, Hickson SS, et al. The impact of cardiovascular risk factors on aortic stiffness and wave reflections depends on age: The Anglo-Cardiff Collaborative Trial (ACCT III). Hypertension [Internet]. 2010;56(4):591–7. Available from: http://ovidsp.ovid.com/ovidweb.cgi?T=JS&PAGE=reference&D=emed12&NEWS=N&AN=359672969

29. McEniery CM, Spratt M, Munnery M, Yarnell J, Lowe GD, Rumley A, et al. An analysis of prospective risk factors for aortic stiffness in men: 20-year follow-up from the

caerphilly prospective study. Hypertension [Internet]. 2010;56(1):36–43. Available from:
http://ovidsp.ovid.com/ovidweb.cgi?T=JS&PAGE=reference&D=emed12&NEWS=N&AN=50954103

**Table S1. Systematic search terms up to 2018 using the Embase database (1974 to 2018 Week 17)**

| | Search terms | Number of results |
|---|---|---|
| 1 | (elast* adj3 arter*).ti,ab. | 4,587 |
| 2 | (pulse wave adj (analysis or velocity)).ti,ab. or focus Pulse Wave/ | 14,902 |
| 3 | ((aortic or arter* or vascular) adj3 (stiff* or compliance or distensibility or characteristic impedance or Zc)).ti,ab. or focus arterial stiffness/ | 24,835 |
| 4 | blood flow velocity.ti,ab. or focus Blood Flow Velocity/ | 8,699 |
| 5 | pulsatile flow.ti,ab. or focus Pulsatile Flow/ | 3,551 |
| 6 | 1 or 2 or 3 or 4 or 5 | 44,010 |
| 7 | ((pre* or borderline or stage-1 or stage-one) adj hypertensi*).ti,ab. | 8,470 |
| 8 | blood pressure.ti,ab. or focus Blood Pressure/ | 371,335 |
| 9 | ((cardiovascular or heart or coronary or vascular) adj2 (disease* or disorder* or event* or risk*)).ti,ab. or focus cardiovascular disease/ or focus heart disease/ or focus vascular disease/ | 676,687 |
| 10 | (stroke or myocardial infarction or arteriosclerosis).ti,ab. or focus cerebrovascular accident/ or focus heart infarction/ or focus arteriosclerosis/ | 528,886 |
| 11 | 7 or 8 or 9 or 10 | 1,361,617 |
| 12 | ((clinical or follow-up or cohort or prospective or retrospective or observational or longitudinal or predict*) adj stud*).ti,ab. or exp prospective study/ or exp clinical study/ or exp longitudinal study/ or exp observational study/ or exp follow up/ or exp retrospective study/ or exp systematic review/ or exp evidence based medicine/ or exp meta analysis/ or exp cohort analysis/ or exp disease association/ or ep.fs. | 10,137,389 |
| 13 | (surviv* or predict* or mortality or fatal* or risk* or associated).ti,ab. or focus all cause mortality/ or focus cardiovascular mortality/ or focus mortality/ or focus mortality rate/ or focus mortality risk/ or focus prediction/ or focus survival analysis/ or focus survival/ or focus survival rate/ or focus disease free survival/ or focus cardiovascular risk/ or focus risk factor/ or focus risk/ | 7,931,343 |
| 14 | 6 and 11 and 12 and 13 | 10,230 |
| 15 | limit 14 to (humans and english language) | 9,537 |

**Table S2. Database: Systematic search terms up to 2018 using the Ovid MEDLINE®, Epub Ahead of Print, In-Process & Other Non-Indexed Citations, Ovid MEDLINE® Daily and Ovid MEDLINE® database (1946 to Present)**

| | **Search terms** | **Number of results** |
|---|---|---|
| 1 | (elast* adj3 arter*).ti,ab. | 3,023 |
| 2 | (pulse wave adj (analysis or velocity)).ti,ab. or exp Pulse Wave Analysis/ | 8,628 |
| 3 | ((aortic or arter* or vascular) adj3 (stiff* or compliance or distensibility or characteristic impedance or Zc)).ti,ab. or exp Vascular stiffness/ | 15,550 |
| 4 | blood flow velocity.ti,ab. or exp Blood Flow Velocity/ | 57,672 |
| 5 | pulsatile flow.ti,ab. or exp Pulsatile Flow/ | 10,570 |
| 6 | 1 or 2 or 3 or 4 or 5 | 81,916 |
| 7 | ((pre* or borderline or stage-1 or stage-one) adj hypertensi*).ti,ab. | 5,751 |
| 8 | blood pressure.ti,ab. or exp Blood Pressure/ | 418,423 |
| 9 | ((cardiovascular or heart or coronary or vascular) adj2 (disease* or disorder* or event* or risk*)).ti,ab. or exp cardiovascular diseases/ or exp heart diseases/ or exp vascular diseases/ | 2,328,416 |
| 10 | (stroke or myocardial infarction or arteriosclerosis).ti,ab. or exp stroke/ or exp myocardial infarction/ or exp arteriosclerosis/ | 579,494 |
| 11 | 7 or 8 or 9 or 10 | 2,596,423 |
| 12 | ((clinical or follow-up or cohort or prospective or retrospective or observational or longitudinal or predict*) adj stud*).ti,ab. or exp prospective studies/ or exp clinical study/ or exp longitudinal studies/ or exp observational study/ or exp retrospective studies/ or exp evidence-based medicine/ or exp review/ or exp meta-analysis/ or exp follow-up studies/ or exp cohort studies/ or ep.fs. | 5,838,938 |
| 13 | (surviv* or predict* or mortality or fatal* or risk* or associate*).ti,ab. or exp risk factors/ or exp mortality/ or exp survival analysis/ or exp disease-free survival/ or exp survival rate/ | 6,329,767 |
| 14 | 6 and 11 and 12 and 13 | 9,428 |
| 15 | limit 14 to (humans and english language) | 8,667 |

**Table S3. Systematic search from 2018 to April 2023 using the Embase database (1974 to 2023 April 05)**

| | Search terms | Number of results |
|---|---|---|
| 1 | elast* adj3 arter*).ti,ab. | 5,982 |
| 2 | (pulse wave adj (analysis or velocity)).ti,ab. or focus Pulse Wave/ | 22,547 |
| 3 | ((aortic or arter* or vascular) adj3 (stiff* or compliance or distensibility or characteristic impedance or Zc)).ti,ab. or focus arterial stiffness/ | 35,663 |
| 4 | blood flow velocity.ti,ab. or focus Blood Flow Velocity/ | 10,478 |
| 5 | pulsatile flow.ti,ab. or focus Pulsatile Flow/ | 4,396 |
| 6 | 1 or 2 or 3 or 4 or 5 | 60,467 |
| 7 | ((pre* or borderline or stage-1 or stage-one) adj hypertensi*).ti,ab. | 11,909 |
| 8 | blood pressure.ti,ab. or focus Blood Pressure/ | 482,665 |
| 9 | ((cardiovascular or heart or coronary or vascular) adj2 (disease* or disorder* or event* or risk*)).ti,ab. or focus cardiovascular disease/ or focus heart disease/ or focus vascular disease/ | 953,729 |
| 10 | (stroke or myocardial infarction or arteriosclerosis).ti,ab. or focus cerebrovascular accident/ or focus heart infarction/ or focus arteriosclerosis/ | 748,174 |
| 11 | 7 or 8 or 9 or 10 | 1,879,845 |
| 12 | ((clinical or follow-up or cohort or prospective or retrospective or observational or longitudinal or predict*) adj stud*).ti,ab. or exp prospective study/ or exp clinical study/ or exp longitudinal study/ or exp observational study/ or exp follow up/ or exp retrospective study/ or exp systematic review/ or exp evidence based medicine/ or exp meta analysis/ or exp cohort analysis/ or exp disease association/ or ep.fs. | 14,215,036 |
| 13 | (surviv* or predict* or mortality or fatal* or risk* or associated).ti,ab. or focus all cause mortality/ or focus cardiovascular mortality/ or focus mortality/ or focus mortality rate/ or focus mortality risk/ or focus prediction/ or focus survival analysis/ or focus survival/ or focus survival rate/ or focus disease free survival/ or focus cardiovascular risk/ or focus risk factor/ or focus risk/ | 11,827,181 |
| 14 | 6 and 11 and 12 and 13 | 17,205 |
| 15 | limit 14 to (humans and english language) | 16,332 |
| 16 | limit 15 to yr="2018 -Current" | 7,035 |

**Table S4. Database: Systematic search terms from 2018 to April 2023 using the Ovid MEDLINE® and In-Process, In-Data-Review & Other Non-Indexed Citations (1946 to April 05, 2023)**

| | Search terms | Number of results |
|---|---|---|
| 1 | (elast* adj3 arter*).ti,ab. | 3,704 |
| 2 | (pulse wave adj (analysis or velocity)).ti,ab. or exp Pulse Wave Analysis/ | 13,001 |
| 3 | ((aortic or arter* or vascular) adj3 (stiff* or compliance or distensibility or characteristic impedance or Zc)).ti,ab. or exp Vascular stiffness/ | 21,546 |
| 4 | blood flow velocity.ti,ab. or exp Blood Flow Velocity/ | 62,970 |
| 5 | pulsatile flow.ti,ab. or exp Pulsatile Flow/ | 11,910 |
| 6 | 1 or 2 or 3 or 4 or 5 | 95,696 |
| 7 | ((pre* or borderline or stage-1 or stage-one) adj hypertensi*).ti,ab. | 7,547 |
| 8 | blood pressure.ti,ab. or exp Blood Pressure/ | 487,490 |
| 9 | ((cardiovascular or heart or coronary or vascular) adj2 (disease* or disorder* or event* or risk*)).ti,ab. or exp cardiovascular diseases/ or exp heart diseases/ or exp vascular diseases/ | 2,895,308 |
| 10 | (stroke or myocardial infarction or arteriosclerosis).ti,ab. or exp stroke/ or exp myocardial infarction/ or exp arteriosclerosis/ | 742,251 |
| 11 | ..nlp 7 or 8 or 9 or 10 {No Related Terms} | 16,956 |
| 12 | ((clinical or follow-up or cohort or prospective or retrospective or observational or longitudinal or predict*) adj stud*).ti,ab. or exp prospective studies/ or exp clinical study/ or exp longitudinal studies/ or exp observational study/ or exp retrospective studies/ or exp evidence-based medicine/ or exp review/ or exp meta-analysis/ or exp follow-up studies/ or exp cohort studies/ or ep.fs. | 7,919,987 |
| 13 | (surviv* or predict* or mortality or fatal* or risk* or associate*).ti,ab. or exp risk factors/ or exp mortality/ or exp survival analysis/ or exp disease-free survival/ or exp survival rate/ | 8,888,264 |
| 14 | ..nlp 6 and 11 and 12 and 13 {No Related Terms} | 10,087 |
| 15 | limit 14 to (humans and english language) | 6,771 |
| 16 | limit 15 to yr="2018 -Current" | 4,254 |

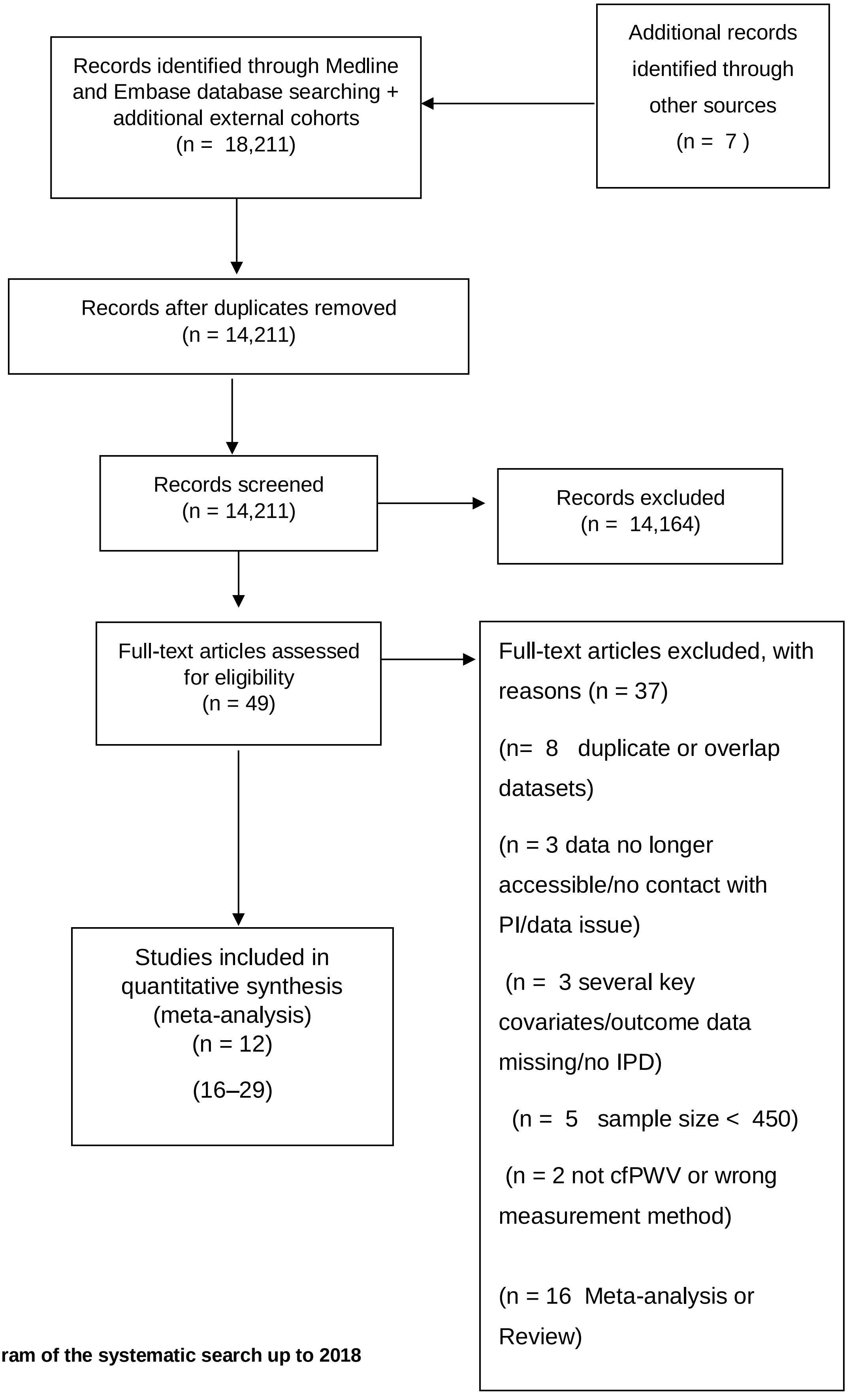


**Figure S1. PRISMA Diagram of the systematic search up to 2018**

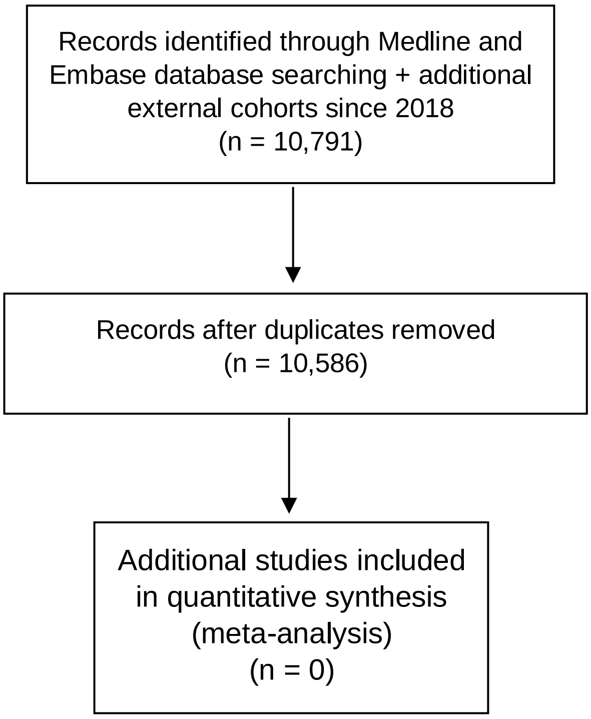


**Figure S2. PRISMA Diagram of the updated systematic search from 2018 to April 2023**

**Table S5. ICD-10 codes/definitions for endpoints used in these analyses**

| Endpoint | ICD code/Definition | Description |
|---|---|---|
| ASCVD | All stroke events, fatal CHD events and non-fatal MI | Fatal or non-fatal cerebrovascular disease, fatal ischemic heart disease (including myocardial infarction, chronic artery disease) and non fatal myocardial infarction. |
| Composite CV events (fatal & non-fatal) (CVD) | I20-I25 + I50 + I60-I64 | Fatal or non-fatal ischemic heart diseases (including myocardial infarction, angina and chronic artery disease), fatal or non-fatal heart failure, fatal or non-fatal cerebrovascular disease. |
| CHD events (fatal & non-fatal) | I20-I25 | Fatal or non-fatal ischemic heart diseases (including myocardial infarction, angina and chronic artery disease). |
| Stroke events (fatal & non-fatal) | I60-I64 | Fatal or non-fatal cerebrovascular disease. |

ICD = International Classification of Diseases, CV = cardiovascular, CHD = coronary heart disease, ASCVD = atherosclerotic cardiovascular disease, MI = myocardial infarction

**Table S6. Methods of CfPWV measurements by study[a]**

| Study | Method of cfPWV measurement | |
|---|---|---|
| | **Waveform** | **Distance** |
| ***Whitehall II*** | SphygmoCor (AtCor Medical, Australia). | Tape measure<br>(Subtracting the carotid-sternal notch distance from the femoral-sternal notch distance). |
| ***ACCT*** | SphymoCor (AtCor Medical, Australia). | Tape measure<br>(Surface distance between the suprasternal notch and femoral site minus the distance between the suprasternal notch and carotid site). |
| ***BLSA*** | CfPWV was calculated using three different devices, due to the long follow-up period:<br>1) transcutaneous Doppler probes (model 810A; 9-10-MHz probes; Parks Medical Electronics, Inc, Aloha, OR),<br>2) Complier SP device (Artech Medical, Paris, France), and<br>3) SphygmoCor system (AtCor Medical, Australia). | Tape measure<br>(to the nearest centimetre) |
| ***DanMONICA*** | Piezoelectric pressure transducers (Hellige GmbH) | Tape measure<br>(On the body surface) |
| ***Health ABC*** | Transcutaneous Doppler probes (model 810A; 9-10-MHz probes; Parks Medical Electronics, Inc, Aloha, OR) | Metal tape measure<br>(Above the surface of the body). |

| | | |
|---|---|---|
| ***Framingham*** | HIHem Data Acquisition and Analysis Workstation (Cardiovascular Engineering, Inc., Norwood, MA) | Tape measure<br>(Difference in body surface measurements from the suprasternal notch to the femoral and carotid sites). |
| ***Kinmen*** | Non-directional Doppler (Parks model 802, Parks Medical Electronics, Inc) | |
| ***EDIVA*** | Complior device (Colson, France) | Tape measure<br>(On the body surface). |
| ***CaPS*** | SphygmoCor device (AtCor Medical) from | Tape measure<br>(On the body surface). |
| ***NSHD*** | SphygmoCor device (AtCor Medical). | Tape measure<br>(On the body surface). |
| ***Rotterdam Study*** | Complier SP device (Complior Artech Medical, France) | Tape measure<br>(On the body surface). |
| ***Hawaii-Los Angeles-Hiroshima*** | NCG400 (Fukunda Denshi, Tokyo, Japan). | Tape measure<br>(On the body surface). |

[a] Details on cfPWV measurement techniques taken from publications referenced and therefore level of detail varies across studies.
CfPWV = carotid-femoral pulse wave velocity

**Table S7. Baseline characteristics of each study (N=16,320)**

| **Study** *(Geographical location)* | **N** | **Age (Years)** mean (SD) | **Male** (n (%)) | **BMI (kg/m²)** median (IQR) | **SBP (mmHg)** mean (SD) | **DBP (mmHg)** mean (SD) | **MAP (mmHg)** mean (SD) | **Pulse Pressure (mmHg)** mean (SD) | **Heart Rate (bpm)** mean (SD) | **cfPWV (m/s)** median (IQR) | **TC[a] (mmol/L)** median (IQR) | **HDL (mmol/L)** median (IQR) | **HRx[b]** (n (%)) | **Diabetes** (n (%)) | **Current Smoker** (n (%)) | **Follow Up (Years)** median (IQR) | **Date of baseline[c]** |
|---|---|---|---|---|---|---|---|---|---|---|---|---|---|---|---|---|---|
| **Whitehall II** *(UK)* | 2,321 | 65.6 (5.8) | 1,837 (79.1) | 26.4 (24.1,28.8) | 133 (9.2) | 75 (8.1) | 94 (8.9) | 57 (8.8) | 68 (11.8) | 8.5 (7.5,9.8) | 5.3 (4.6,6.0) | 1.5 (1.3,1.8) | 769 (33.1) | 185 (8.0) | 108 (4.7) | 9.0 (8.6,9.4) | 2007-2009 |
| **ACCT** *(UK)* | 1,998 | 62.5 (12.2) | 925 (46.3) | 26.4 (24.1,29.2) | 136 (11.2) | 82 (7.8) | 101 (8.0) | 55 (11.2) | 71 (11.4) | 8.4 (7.2,9.7) | 5.4 (4.6,6.1) | 1.5 (1.2,1.8) | 458 (22.9) | 54 (2.7) | 163 (8.2) | 10.1 (8.5,13.0) | 2000-2004 |
| **BLSA** *(North America)* | 437 | 58.9 (14.7) | 203 (46.5) | 26.3 (23.7,30.1) | 130 (11.3) | 78 (9.7) | 95 (9.6) | 55 (16.7) | 68 (11.1) | 6.7 (5.2,8.1) | 5.1 (4.6,5.8) | 1.3 (1.1,1.6) | 18 (4.1) | 19 (4.3) | 11 (2.5) | 10.3 (4.3,13.7) | 1988 |
| **DanMONICA** *(Denmark)* | 1,541 | 55.4 (10.4) | 808 (52.4) | 25.9 (23.6,28.7) | 132 (11.9) | 84 (7.2) | 100 (7.2) | 51 (11.1) | 67 (10.5) | 11.0 (9.6,12.6) | 6.2 (5.5,6.9) | 1.4 (1.1,1.7) | 137 (8.9) | 41 (2.7) | 652 (42.3) | 12.6 (12.2,13.0) | 2008 |
| **Health ABC** *(North America)* | 1,168 | 73.6 (2.9) | 521 (44.6) | 26.9 (24.2,30.0) | 137 (11.0) | 72 (10.0) | 92 (8.7) | 64 (12.0) | 65 (10.6) | 8.0 (6.4,10.5) | 5.2 (4.6,5.8) | 1.4 (1.1,1.7) | 562 (48.1) | 165 14.1) | 119 (10.2) | 13.0 (9.5,13.4) | 1997-1998 |
| **Framingham** *(North America)* | 2,682 | 51.1 (14.2) | 1,493 (55.7) | 27.6 (24.7,30.8) | 130 (10.7) | 80 (8.3) | 95 (9.8) | 56 (11.8) | 64 (10.3) | 8.2 (7.0,9.8) | 5.1 (4.6,5.7) | 1.3 (1.1,1.6) | 610 (22.7) | 166 (6.2) | 350 (13.0) | 11.7 (10.1,14.1) | 1991-2005[4] |
| **Kinmen** *(Taiwan)* | 572 | 51.6 (12.7) | 322 (56.3) | 24.3 (22.3,26.7) | 132 (11.1) | 85 | 99 | 46 | 74 (9.8) | 8.7 (7.5,10.2) | 4.9 | 1.3 (1.1,1.5) | 0 | 16 | 151 (26.4) | 19.9 (12.3,20.3) | 1992-1993 |

| | | | | | | | | | | | | | | | | | |
|---|---|---|---|---|---|---|---|---|---|---|---|---|---|---|---|---|---|
| | | | | | | (9.6) | (8.9) | (10.2) | | | (4.4,5.7) | | (0.0) | (2.8) | | | |
| **EDIVA** *(Portugal)* | 1,403 | 44.8 (13.7) | 891 (63.5) | 26.3 (24.0,29.4) | 136 (10.0) | 82 (8.6) | 100 (8.1) | 54 (9.6) | 70 (11.7) | 9.5 (8.5,10.9) | 4.7 (4.0,5.6) | 1.2 (1.1,1.4) | 629 (44.8) | 127 (9.1) | 269 (19.2) | 1.6 (1.0,3.0) | 2002-2004 |
| **CaPS** *(UK)* | 394 | 72.3 (3.9) | 394 (100.0) | 27.1 (25.5,29.2) | 139 (10.6) | 75 (9.1) | 97 (8.6) | 65 (12.0) | 69 (12.2) | 11.1 (9.6,12.7) | 4.9 (4.2,5.7) | 1.3 (1.1,1.5) | 172 (43.7) | 38 (9.6) | 62 (15.7) | 5.5 (5.1,6.0) | 2002-2004 |
| **NSHD** *(UK)* | 681 | 63.7 (0.6) | 322 (47.3) | 27.0 (24.6, 30.3) | 137 (10.6) | 79 (8.0) | 96 (7.8) | NA | 69 (10.7) | 8.1 (7.3, 9.1) | 5.7 (5.1, 6.6) | 1.6 (1.3, 1.9) | 114 (16.7) | 35 (5.1) | 45 (6.6) | 5.7 (5.3, 6.2) | 2006-2011 |
| **Rotterdam Study** *(Netherlands)* | 2,790 | 66.3 (9.4) | 1,141 (40.9) | 26.7 (24.4, 29.3) | 137 (11) | 78 (10) | 96 (8) | 59 (13) | 70 (11) | 11.2 (9.4, 3.4) | 5.8 (5.1, 6.4) | 1.4 (1.1, 1.6) | 846 (30.3) | 288 (10.3) | 588 (21.1) | 10.0 (7.5, 16.6) | 1997-1999 |
| **Hawaii-Los Angeles-Hiroshima**[e] *(North America)* | 333 | 64.0 (8.4) | 165 (49.5) | 23.9 (21.6,25.8) | 134 (11.5) | 80 (7.6) | 96 (7.2) | 54 (11.5) | N/A | 9.3 (8.6,10.5) | 5.8 (5.3,6.6) | 1.2 (1.0,1.4) | N/A | 49 (14.7) | N/A | 10.0 (10.0,10.0) | 1984 |

[a]*TC=total cholesterol.*

[b]*HRx = antihypertensive medications.*

[c]*First* examination where cfPWV was measured.

[d]*The Framingham study data consisted of four cohorts with different baseline examination dates where cfPWV was measured:* the 26th examination of the original cohort (1999-2001), the seventh examination of the offspring cohort (1998-2001), the 2nd examination of the OMNI 1 cohort (1998) and the 1st examination of the Gen3 cohort (2002-2005).

[e]*Only contributed to sensitivity analyses after missing data were multiply imputed.*

Studies underlined were included in the model derivation and validation. Health ABC was excluded for sensitivity analyses.

**Table S8. Numbers of events within each study (N=16,320)**

| Study | N | ASCVD (n(%)) | All-cause mortality (n(%)) | Fatal and non-fatal events (n(%)) | | | Fatal events (n(%)) | | | Non-fatal events (n(%)) | | |
|---|---|---|---|---|---|---|---|---|---|---|---|---|
| | | | | CVD | CHD | Stroke | CVD | CHD | Stroke | CVD | CHD | Stroke |
| **Whitehall II** | 2,321 | 98 (4.2) | 143 (6.2) | 243 (10.5) | 197 (8.5) | 40 (1.7) | 30 (1.3) | 14 (0.6) | 2 (0.1) | 222 (9.6) | 187 (8.1) | 39 (1.7) |
| **ACCT** | 1,998 | 21 (1.1) | 105 (5.3) | 21 (1.1) | 17 (0.9) | 4 (0.2) | 21 (1.1) | 17 (0.9) | 4 (0.2) | N/A | N/A | N/A |
| **BLSA** | 437 | 3 (0.7) | 18 (4.1) | 5 (1.1) | 2 (0.5) | 1 (0.2) | 5 (1.1) | 2 (0.5) | 1 (0.2) | N/A | N/A | N/A |
| **DanMONICA** | 1,541 | 179 (11.6) | 269 (17.5) | 256 (16.6) | 126 (8.2) | 119 (7.7) | 30 (1.9) | 22 (1.4) | 8 (0.5) | 249 (16.2) | 116 (7.5) | 119 (7.7) |
| **Health ABC** | 1,168 | 282 (24.1) | 695 (59.5) | 365 (31.3) | 269 (23.0) | 133 (11.4) | 130 (11.1) | 68 (5.8) | 62 (5.3) | 253 (21.7) | 201 (17.2) | 71 (6.1) |
| **Framingham** | 2,682 | 203 (7.6) | 303 (11.3) | 269 (10.0) | 122 (4.5) | 91 (3.4) | 49 (1.8) | 21 (0.8) | 10 (0.4) | 253 (9.4) | 107 (4.0) | 91 (3.4) |
| **Hawaii-Los Angeles-Hiroshima**[a] | 333 | 6 (1.8) | 29 (8.7) | 6 (1.8) | 3 (0.9) | 3 (0.9) | 10 (3.0) | 3 (0.9) | 3 (0.9) | N/A | N/A | N/A |
| **Kinmen** | 572 | 19 (3.3) | 126 (22.0) | 25 (4.4) | 6 (1.0) | 13 (2.3) | 25 (4.4) | 6 (1.0) | 13 (2.3) | N/A | N/A | N/A |
| **EDIVA** | 1,403 | 17 (1.2) | 0 (0.0) | 17 (1.2) | 7 (0.5) | 10 (0.7) | 0 (0.0) | 0 (0.0) | 0 (0.0) | 17 (1.2) | 7 (0.5) | 10 (0.7) |
| **CaPS** | 394 | 44 (11.2) | 51 (12.9) | 44 (11.2) | 23 (5.8) | 21 (5.3) | 7 (1.8) | 3 (0.8) | 4 (1.0) | 38 (9.6) | 20 (5.1) | 18 (4.6) |
| **NSHD** | 681 | 18 (2.6) | 18 (2.6) | 33 (4.8) | 20 (2.9) | 13 (1.9) | 1 (0.1) | 0 (0.0) | 1 (0.1) | 32 (4.7) | 20 (2.9) | 12 (1.8) |
| **Rotterdam Study** | 2,790 | 395 (14.2) | 1,139 (40.8) | 518 (18.6) | 304 (10.9) | 247 (8.9) | 106 (3.8) | 41 (1.5) | 65 (2.3) | 421 (15.1) | 263 (9.4) | 182 (6.5) |

[a]*Only contributed to sensitivity analyses after missing data were multiply imputed.*

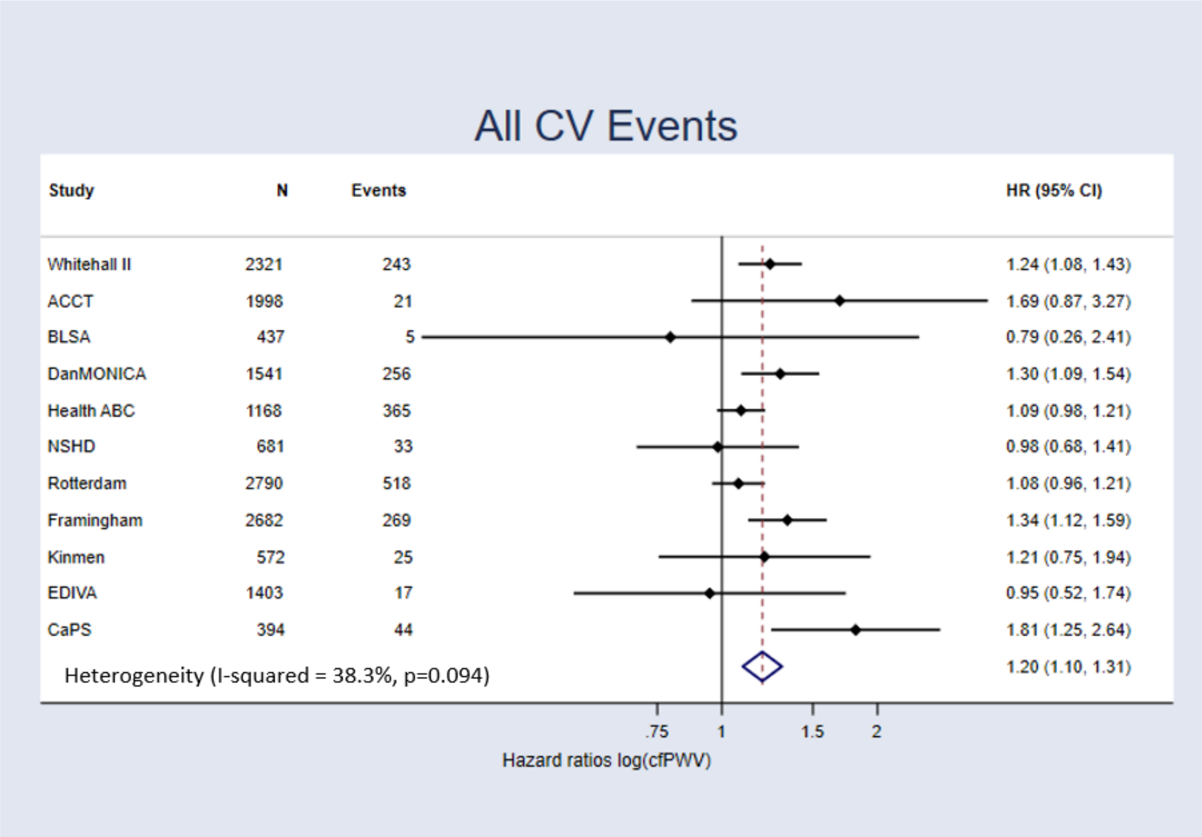


**Figure S3. Forest plot of the hazard ratios for the effect of a 1-SD higher log$_e$(cfPWV) on the risk of all cardiovascular events. Hazard ratios adjusted for age, sex, SBP, HDL-C, total cholesterol, smoking status, diabetes and antihypertensive medications. Pooled hazard ratio is estimated from a random effects meta-analysis, weighted by the number of events.**
**N=15,987**

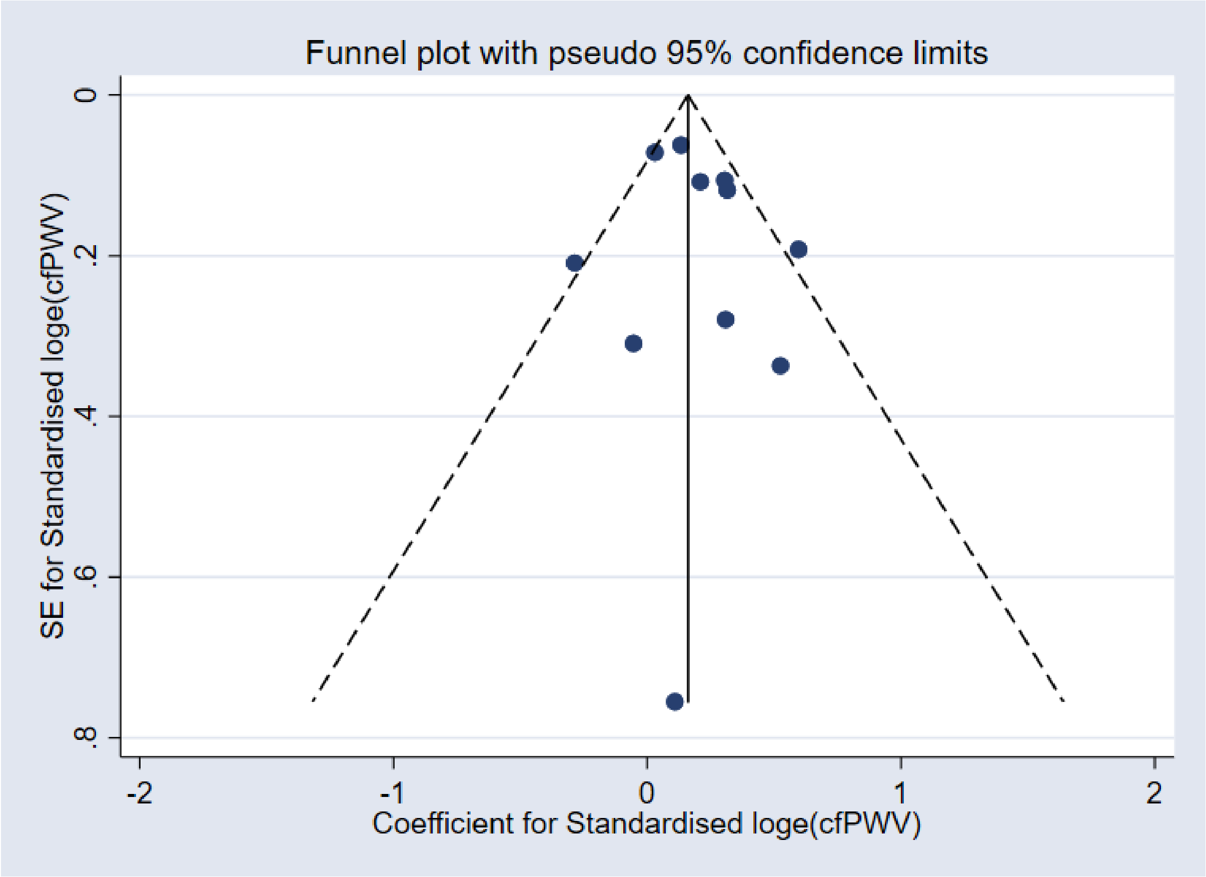


**Figure S4. Funnel plot for to show study bias of cfPWV in multivariate models. Studies where no individual participant data was available were not included.**

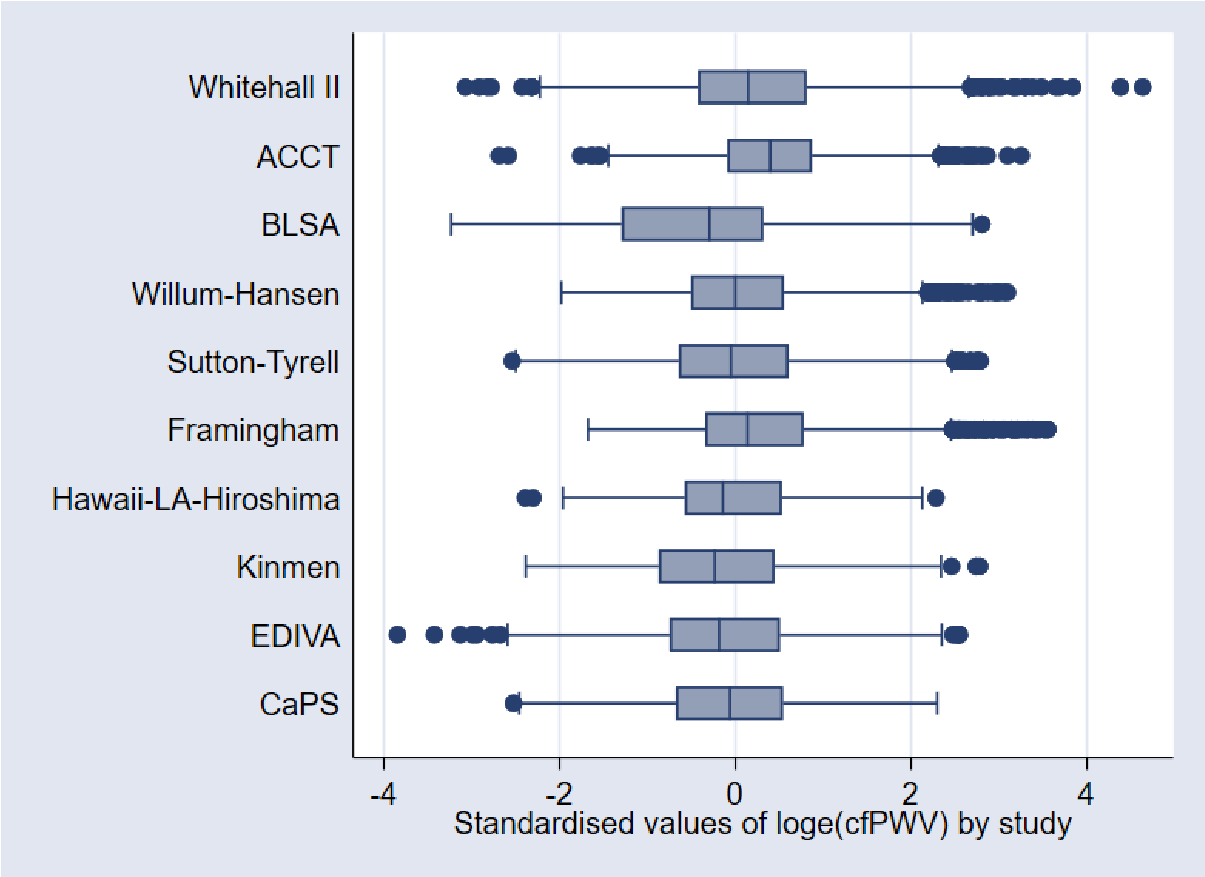


**Figure S5. Box plot by study showing the distributions of log transformed cfPWV. Studies where no individual participant data was available were not included.**

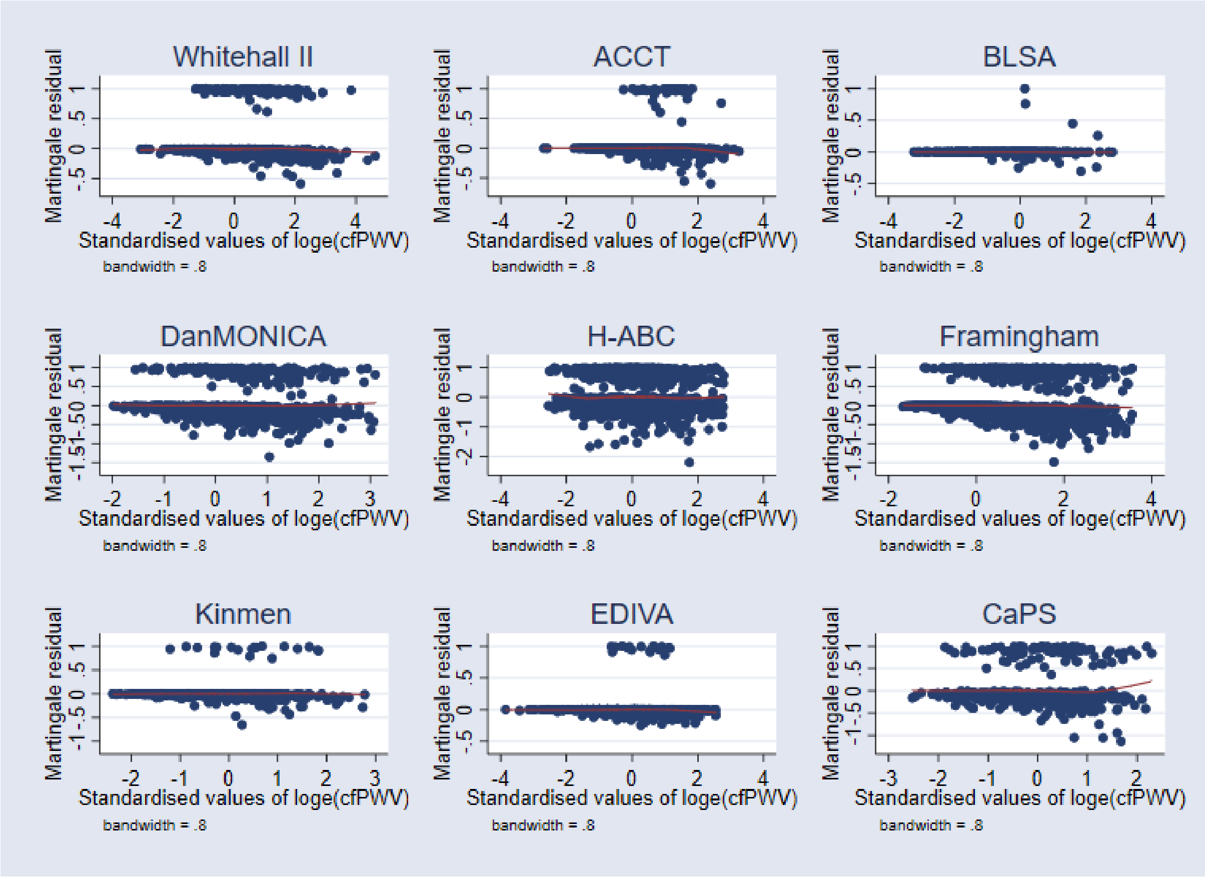


**Figure S6. Martingales residuals vs. standardised values of log$_e$(cfPWV) to assess linearity in the fully adjusted models for time to ASCVD. Studies where no individual participant data was available were not included.**

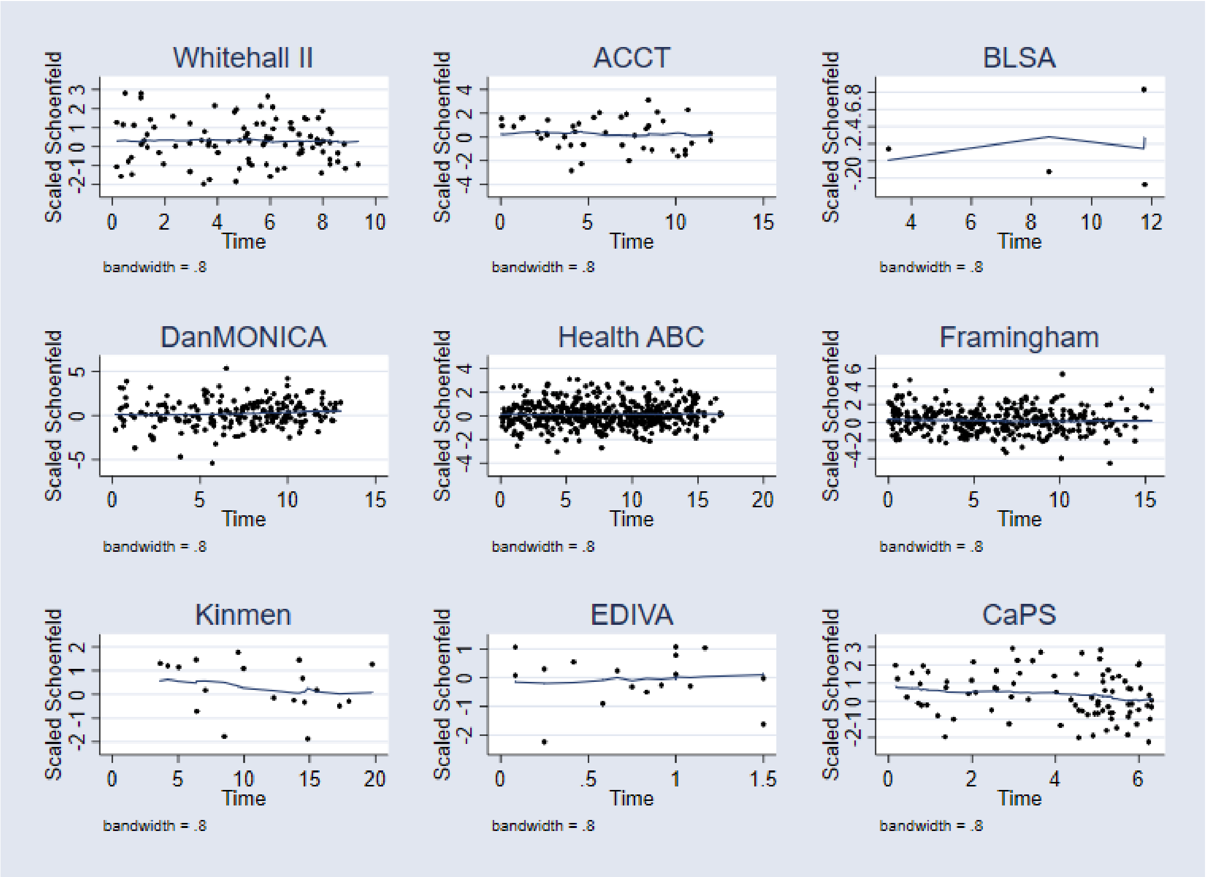


**Figure S7. Schoenfeld residuals vs. standardised values of log$_e$(cfPWV) to assess proportional hazards in the fully adjusted models for time to ASCVD. Studies where no individual participant data was available were not included.**

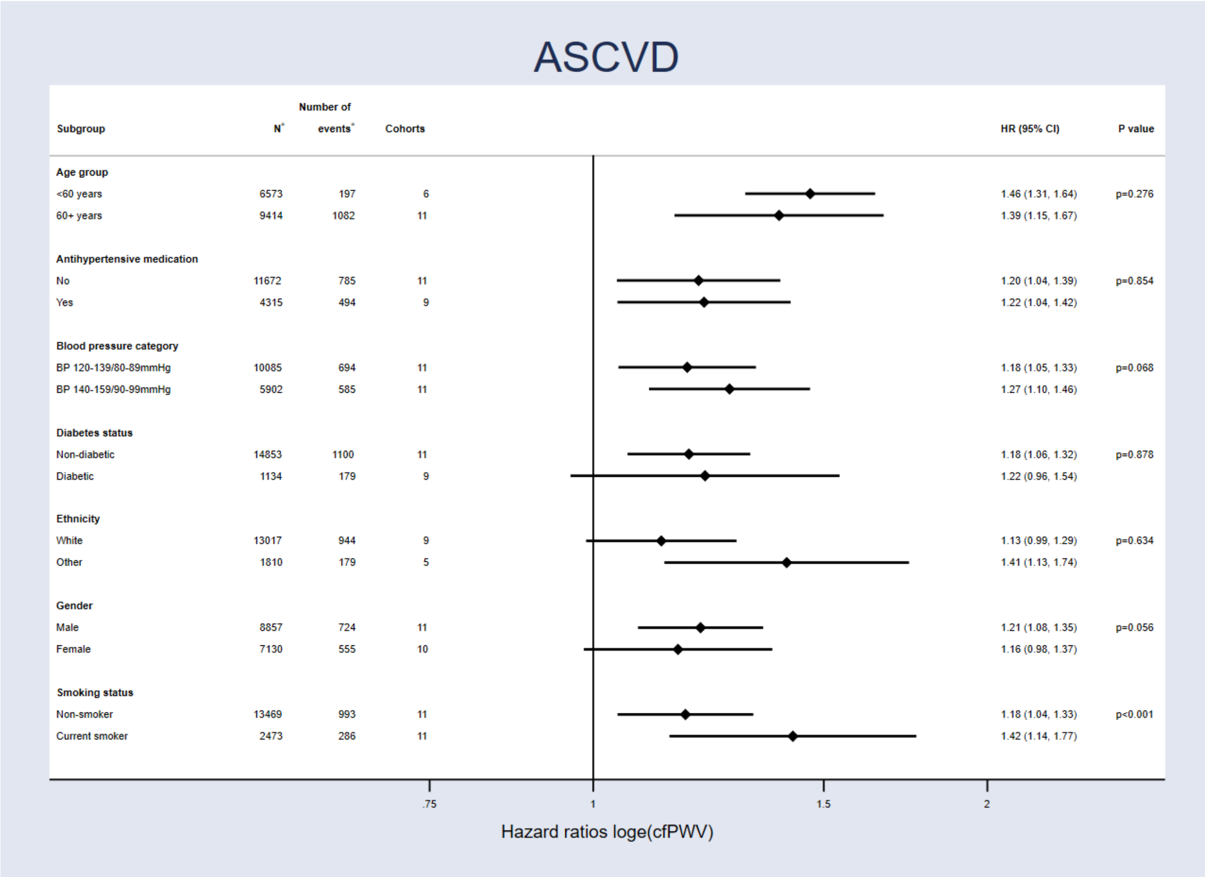


**Figure S8. Forest plot to assess the effect of cfPWV on the risk of ASCVD by pre-specified subgroups. Hazard ratios are for a 1-SD change in $\log_e$(cfPWV) on the risk of all CV events. Hazard ratios adjusted for age, sex, SBP, HDL, smoking status, diabetes and antihypertensive medications, where appropriate. Results are pooled across studies in a random effects meta-analysis.**

**N=15,987**

*Total N and number of events do not equate to total number of individuals and total number of ASCVD events for ethnicity due to some missing ethnicity data.
P-values calculated from studies where IPD was available.

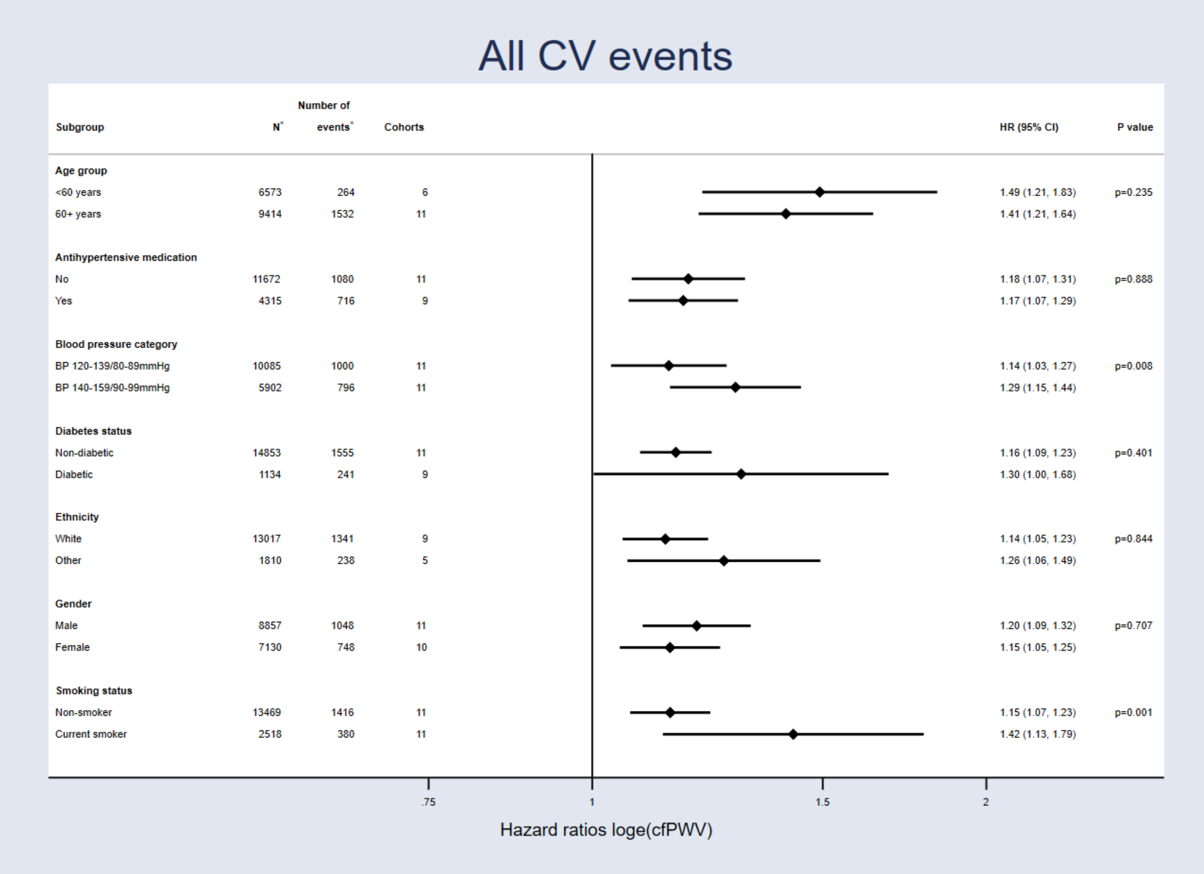


**Figure S9. Forest plot to assess the effect of cfPWV on the risk of CV events by pre-specified subgroups. Hazard ratios are for a 1-SD change in log$_e$(cfPWV) on the risk of all CV events. Hazard ratios adjusted for age, sex, SBP, HDL, smoking status, diabetes and antihypertensive medications, where appropriate. Results are pooled across studies in a random effects meta-analysis.**

**N=15,987**

*Total N and number of events do not equate to total number of individuals and total number of CV events for ethnicity due to some missing ethnicity data.
P-values calculated from studies where IPD was available.

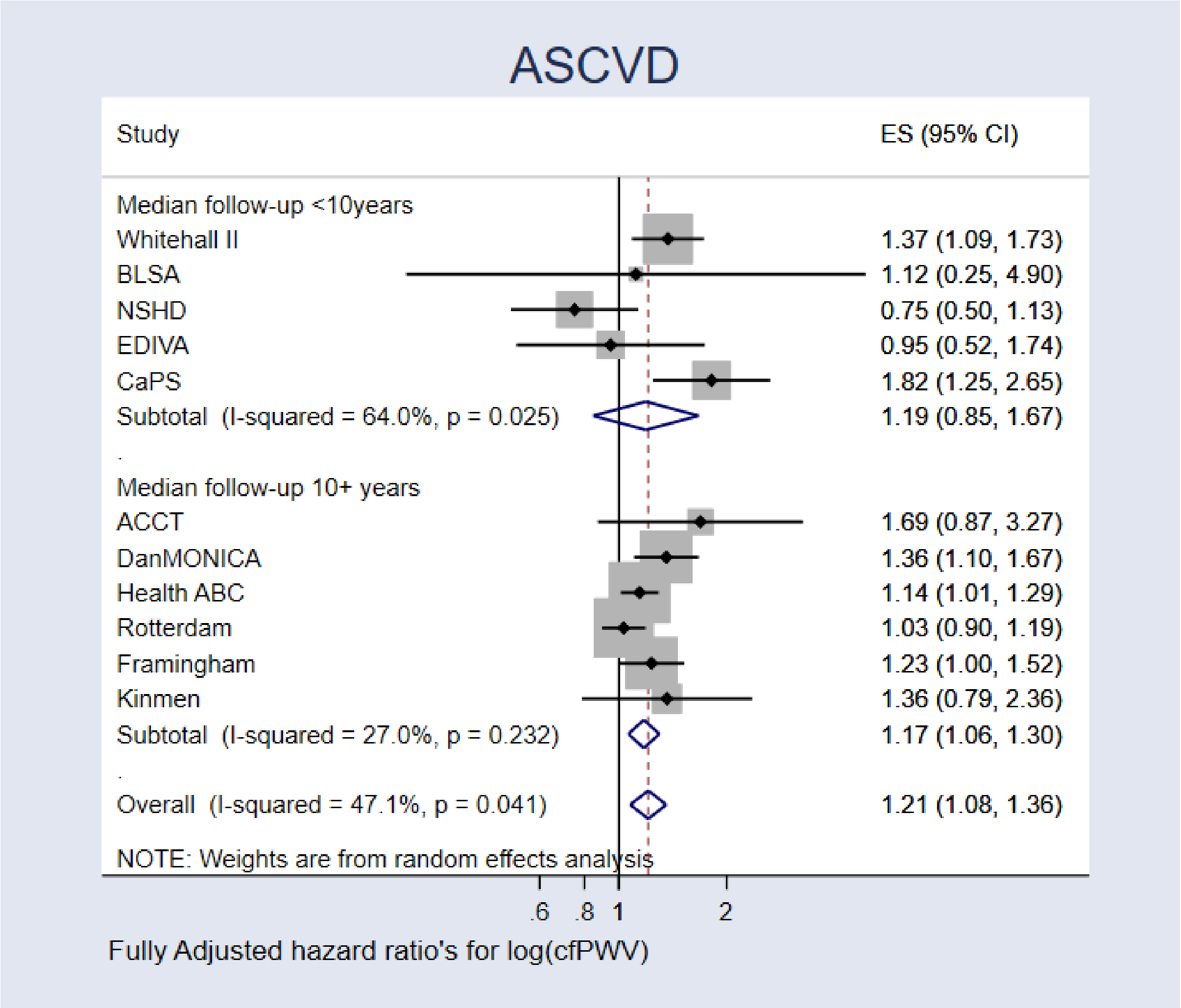


**Figure S10. Forest plot of the hazard ratios for log$_e$(cfPWV) on the risk of ASCVD events stratified by follow-up time <10 years and ≥10 years**

**N=15,987**

**Table S9. Hazard ratios (HR) for $log_e$(cfPWV) with missing data multiply imputed (N=19,867)**

| Outcome | HR (95% CI) | Events |
|---|---|---|
| ASCVD | 1.20 (1.09,1.33) | 1,607 |
| All-cause Mortality | 1.12 (1.07,1.17) | 3,597 |
| All CV Events | 1.21 (1.11,1.31) | 2,303 |
| Fatal CV Events | 1.23 (1.10,1.37) | 517 |
| Non-fatal CV Events | 1.17 (1.07,1.29) | 1,914 |
| All CHD Events | 1.17 (1.06,1.30) | 1,398 |
| Fatal CHD Events | 1.23 (1.04,1.46) | 194 |
| Non-fatal CHD Events | 1.17 (1.05,1.32) | 1,188 |
| All Stroke Events | 1.20 (1.10,1.30) | 868 |
| Fatal Stroke Events | 1.23 (1.02,1.49) | 138 |
| Non-fatal Stroke Events | 1.18 (1.07,1.31) | 685 |

**Table S10. Hazard ratios (HR) and 95% confidence intervals (CI) for $\log_e$(cfPWV) for each outcome, including only studies who contribute to all analyses (N = 10,896)**

| | **HR (95% CI) for $\log_e$(cfPWV)** | | | |
|---|---|---|---|---|
| | **Model 1** | **Model 2** | **Model 3** | **Model 4** |
| | **(Crude)** | **Model 1 with sex, MAP & heart rate** | **Model 2 with age** | **Model 1 with traditional risk factors[1]** |
| **ASCVD** | 1.70 (1.42,2.03) | 1.69 (1.39,2.05) | 1.28 (1.13,1.44) | 1.29 (1.14,1.46) |
| **All cause-mortality** | 1.74 (1.36,2.22) | 1.71 (1.31,2.22) | 1.12 (1.07,1.18) | 1.13 (1.08,1.18) |
| **All CVD events** | 1.70 (1.37,2.11) | 1.70 (1.36,2.12) | 1.25 (1.14,1.38) | 1.22 (1.11,1.34) |
| **Fatal CVD events** | 2.01 (1.42,2.86) | 1.99 (1.41,2.81) | 1.26 (1.04,1.52) | 1.23 (1.07,1.41) |
| **Non-Fatal CVD events** | 1.67 (1.34,2.09) | 1.67 (1.32,2.11) | 1.26 (1.12,1.41) | 1.22 (1.09,1.37) |
| **All CHD events** | 1.56 (1.29,1.89) | 1.54 (1.26,1.89) | 1.24 (1.08,1.41) | 1.20 (1.07,1.35) |
| **Fatal CHD events** | 2.04 (1.50,2.76) | 1.97 (1.44,2.69) | 1.33 (1.10,1.61) | 1.33 (1.10,1.61) |
| **Non-Fatal CHD events** | 1.50 (1.24,1.82) | 1.49 (1.21,1.83) | 1.22 (1.06,1.42) | 1.17 (1.04,1.31) |
| **All Stroke events** | 1.77 (1.51,2.07) | 1.78 (1.50,2.11) | 1.27 (1.15,1.40) | 1.21 (1.11,1.32) |
| **Fatal Stroke events** | 2.26 (1.57,3.25) | 2.23 (1.55,3.19) | 1.36 (1.08,1.71) | 1.27 (1.07,1.51) |
| **Non-Fatal Stroke events** | 1.75 (1.50,2.06) | 1.75 (1.47,2.08) | 1.24 (1.10,1.38) | 1.19 (1.08,1.31) |

[1]Age, sex, SBP, total cholesterol, smoking status, HDL-cholesterol, diabetes status and antihypertensive medications

Cohorts included: Whitehall II, DanMONICA, Health ABC, Rotterdam Study, Framingham, CaPS

**Table S11. Sensitivity analyses**

| | | ASCVD | All CV Events |
|---|---|---|---|
| | N | **Fully adjusted hazard ratio for $\log_e$(cfPWV)[1]** | **Fully adjusted hazard ratio for $\log_e$(cfPWV)[1]** |
| **Pre/Stage – 1 hypertension[2]** | 15,987 | 1.21 (1.08, 1.36) | 1.20 (1.10, 1.31) |
| Pre/Stage-1 hypertension with missing data multiply imputed | 19,867 | 1.20 (1.09,1.33) | 1.21 (1.11,1.31) |
| Middle risk defined by recalibrated Framingham CVD risk equation (10-20% predicted 10y risk) (Ages 40-70)[3] | 4,021 | 1.29 (1.07, 1.56) | 1.22 (1.06, 1.40) |
| Middle risk defined by original Framingham CVD risk equation (10-20% predicted 10y risk) (Ages 40-70)[3] | 4,985 | 1.25 (1.10, 1.42) | 1.25 (1.10, 1.41) |
| Middle risk defined by recalibrated PCE's (7.5%-20% predicted 10y risk) (Ages 40-70)[3] | 5,672 | 1.33 (1.15, 1.54) | 1.24 (1.10, 1.40) |
| Middle risk defined by original PCE's (7.5%-20% predicted 10y risk) (Ages 40-70)[3] | 4,970 | 1.27 (1.13, 1.44) | 1.22 (1.09, 1.36) |
| Middle risk defined by SCORE2 calibrated to low-risk countries (Ages 40-70)[3] | 5,602 | 1.52 (1.19, 1.94) | 1.34 (1.15, 1.56) |
| Studies contributing to all endpoints | 10,896 | 1.29 (1.14, 1.46) | 1.22(1.11 1.34) |
| Untransformed cfPWV | 15,987 | 1.19 (1.07, 1.32) | 1.18 (1.08, 1.27) |

[1]Hazard ratios for untransformed cfPWV where specified

[2]Primary analyses

[3]NSHD and Rotterdam Study cohorts not included as no individual participant data available

[4]Whitehall II, ACCT, NSHD and CaPS contribute to the UK analyses

**Table S12. Summary statistics for middle risk individuals without a history of CV by blood pressure category and cfPWV category (N=12,516)**

| | N | ASCVD Events | CVD Events | Male | Age (Years) | cfPWV (m/s) | SBP (mmHg) | DBP (mmHg) |
|---|---|---|---|---|---|---|---|---|
| | N (%) | n (%) | n (%) | n (%) | mean (SD) | median (IQR) | Mean (SD) | Mean (SD) |
| **Low cfPWV, pre-hypertension** | 4,996 (39.9) | 207 (4.1) | 309 (6.2) | 2,901 (58) | 54.5 (14.5) | 7.5 (6.7, 8.2) | 127 (7) | 77 (7) |
| **Low cfPWV, stage-1 hypertension** | 1,706 (13.6) | 87 (5.1) | 120 (7.0) | 950 (56) | 57.3 (14.5) | 7.8 (7.0, 8.4) | 143 (9) | 85 (9) |
| **High cfPWV, pre-hypertension** | 3,187 (25.5) | 302 (9.5) | 439 (13.8) | 1,965 (62) | 61.9 (12.6) | 10.5 (9.7, 11.9) | 129 (7) | 76 (8) |
| **High cfPWV, stage-1 hypertension** | 2,627 (21.0) | 270 (10.3) | 377 (14.4) | 1,578 (60) | 62.7 (12.5) | 11.1 (9.9, 12.8) | 146 (7) | 84 (9) |

*Shokawa, NSHD and Rotterdam Study cohorts not included.

Percentages calculated within each category.

**Table S13. Adjusted[a] hazard ratios[b] for stage of hypertension and dichotomous cfPWV for CV events (N=12,516[c])**

| | **(BP: 120-139/80-89mmHg)** | **(BP: 140-159/90-99mmHg)** |
|---|---|---|
| **Low cfPWV (≤9m/s)** | **Ref:** 1.00 (0.88, 1.14) | 1.01 (0.78, 1.29), 0.965 |
| **High cfPWV (>9m/s)** | 1.39 (1.14, 1.70), p=0.001 | 1.55 (1.39, 1.73), p<0.001 |

[a]not adjusting for continuous cfPWV or SBP, hazard ratios for categorical cfPWV and hypertensive status after adjusting for age, sex, smoking status, total cholesterol, HDL, anti-hypertensive medications and diabetes status.

[b]Floating hazard ratios with pre-hypertension and low cfPWV as the reference group.

[c]NSHD and Rotterdam Study not included as no IPD available.

CfPWV = carotid-femoral pulse wave velocity, CV = cardiovascular, BP = blood pressure

**Table S14. Measures of the prediction improvement after the addition of $\log_e$(cfPWV) to traditional CV risk factors when predicting CV events (N=5,391).**

| | Categorical Prospective NRI (95% CI), p-value | | |
|---|---|---|---|
| | **Total** | **Event** | **Non-event** |
| **All CV Events** | 0.0055 (-0.0187, 0.0296), p=0.656 | -0.0079 (-0.0413, 0.0256), p=0.644 | 0.0123 (-0.0021, 0.0266), p=0.093 |
| **ASCVD** | 0.0088 (-0.0495, 0.0671), p=0.768 | -0.0023 (-0.0518, 0.0472), p=0.928 | 0.0070 (-0.0061, 0.0201), p=0.298 |
| | | | |
| | **Integrated discrimination improvement (IDI) (95% CI), p-value** | | |
| | **Total** | **Cases** | **Controls** |
| **All CV Events** | 0.0047 (0.0018, 0.0077), p=0.001 | 0.0039 (0.0011, 0.0068), p=0.007 | 0.0008 (0.0002, 0.0015), p=0.015 |
| **ASCVD** | 0.0047 (0.0016, 0.0078), p=0.003 | 0.0040 (0.0010, 0.0071), p=0.009 | 0.0007 (0.0001, 0.0013), p=0.034 |

Only studies where IPD was available and who had median follow-up ≥10 years were included: DanMONICA, Health ABC, Framingham Heart Study, CaPS.

CfPWV = carotid-femoral pulse wave velocity, CV = cardiovascular, NRI = net reclassification index, CI = confidence interval, ASCVD = atherosclerotic cardiovascular disease

**Table S15. Numbers of events by sex for the four studies included in the model derivations and validations (Ages 40-79)**

| Study | Males | | | | | Females | | | | |
|---|---|---|---|---|---|---|---|---|---|---|
| | **ASCVD** | **All CV events** | **Fatal CV events** | **All CHD** | **All Stroke** | **ASCVD** | **All CV events** | **Fatal CV events** | **All CHD** | **All Stroke** |
| **Whitehall II** | 81 (4.4) | 197 (10.7) | 24 (1.3) | 161 (8.8) | 33 (1.8) | 17 (3.5) | 46 (9.5) | 6 (1.2) | 36 (7.4) | 7 (1.5) |
| **DanMONICA** | 95 (11.8) | 126 (15.6) | 19 (2.4) | 73 (9.0) | 53 (6.6) | 44 (6.0) | 63 (8.6) | 3 (0.4) | 25 (3.4) | 38 (5.2) |
| **Health ABC** | 88 (17.0) | 134 (25.9) | 42 (8.1) | 112 (21.6) | 33 (6.4) | 89 (13.8) | 125 (19.4) | 40 (6.2) | 87 (13.5) | 52 (8.1) |
| **Framingham** | 82 (7.7) | 108 (10.1) | 11 (1.0) | 57 (5.4) | 28 (2.6) | 45 (4.8) | 66 (7.1) | 7 (0.8) | 33 (3.6) | 12 (1.3) |

ASCVD = all Stroke events, fatal CHD events and non-fatal myocardial infarction
CVD = all Stroke events, all CHD events and heart failure
N=7,019

**Table S16. Bootstrap validated C-statistics and Brier score for the novel cfPWV risk model predicting 10-year ASCVD risk adjusting for risk factors included in the Pooled Cohort Equations (excluding interaction terms) + cfPWV (Ages 40-79).**

| | | | Whitehall II, DanMONICA, Health ABC, Framingham (N=7,019, male: 4,228, female: 2,791) (Ages 40-79) | | | Whitehall II, DanMONICA, Framingham (N=5,856, male: 3,710, female: 2,146) (Ages 40-79) | | |
|---|---|---|---|---|---|---|---|---|
| **Measure** | **Gender** | **Outcome** | **Apparent** | **Corrected** | **Events** | **Apparent** | **Corrected** | **Events** |
| **C-statistic** | **Female** | **ASCVD** | 0.6790 (0.6374,0.7206) | 0.6657 (0.6241,0.7073) | 195 | 0.7318 (0.6809,0.7827) | 0.7207 (0.6698,0.7716) | 106 |
| | **Male** | **ASCVD** | 0.6738 (0.6454,0.7021) | 0.6711 (0.6427,0.6995) | 346 | 0.7003 (0.6680,0.7327) | 0.6983 (0.6659,0.7306) | 258 |
| | | | | | | | | |
| **Brier score** | **Female** | **ASCVD** | 0.0781 (0.0690,0.0872) | 0.0795 (0.0704,0.0885) | 195 | 0.0454 (0.0378,0.0530) | 0.0462 (0.0386,0.0538) | 106 |
| | **Male** | **ASCVD** | 0.0888 (0.0814,0.0963) | 0.0892 (0.0817,0.0966) | 346 | 0.0711 (0.0636,0.0785) | 0.0713 (0.0638,0.0788) | 258 |

**Table S17. Bootstrap validated C-statistics and Brier score for the novel cfPWV risk model predicting 10-year ASCVD adjusting for covariates in SCORE2 + cfPWV (Ages 40-79)**

| | | | Whitehall II, DanMONICA, Health ABC, Framingham (N=7,019, male: 4,228, female: 2,791) (Ages 40-79) | | | Whitehall II, DanMONICA, Framingham (N=5,856, male: 3,710, female: 2,146) (Ages 40-79) | | |
|---|---|---|---|---|---|---|---|---|
| **Measure** | **Gender** | **Outcome** | **Apparent** | **Corrected** | **Events** | **Apparent** | **Corrected** | **Events** |
| **C-statistic** | **Female** | **ASCVD** | 0.6746 (0.6332,0.7159) | 0.6657 (0.6244,0.7071) | 195 | 0.7283 (0.6780,0.7786) | 0.7211 (0.6708,0.7714) | 106 |
| | **Male** | **ASCVD** | 0.6763 (0.6472,0.7053) | 0.6645 (0.6355,0.6936) | 346 | 0.7008 (0.6675,0.7341) | 0.6910 (0.6577,0.7243) | 258 |
| | | | | | | | | |
| **Brier score** | **Female** | **ASCVD** | 0.0781 (0.0690,0.0872) | 0.0795 (0.0704,0.0885) | 195 | 0.0454 (0.0378,0.0530) | 0.0462 (0.0386,0.0538) | 106 |
| | **Male** | **ASCVD** | 0.0885 (0.0810,0.0960) | 0.0895 (0.0820,0.0970) | 346 | 0.0710 (0.0635,0.0785) | 0.0717 (0.0642,0.0793) | 258 |

**Table S18. Bootstrap validated NRI for 10year ASCVD risk comparing the novel cfPWV risk score for ASCVD with the recalibrated Pooled Cohort Equations (Ages 40-79)**

| | | | **Prospective NRI** | | |
|---|---|---|---|---|---|
| **Studies included** | **Outcome** | **Events** | **N** | **NRI (Apparent)** | **NRI (Corrected)** |
| **Whitehall II, DanMONICA, Health ABC, Framingham** | **ASCVD** | 541 | 4,589 | 3.86 (-2.00,9.73) | 1.34 (-4.53,7.20) |
| | | | | | |
| **Whitehall II, DanMONICA, Framingham** | **ASCVD** | 364 | 3,645 | 0.70 (-5.70,7.10) | -1.88 (-8.28,4.53) |

**Table S19. Bootstrap validated NRI for 10year ASCVD risk comparing the novel cfPWV risk score for ASCVD with the SCORE2 equation calibrated to low-risk populations. Ages 40-79**

| | | | Prospective NRI | | |
|---|---|---|---|---|---|
| | **Outcome** | **Events** | **N** | **NRI (Apparent)** | **NRI (Corrected)** |
| **Whitehall II, DanMONICA, Health ABC, Framingham** | **ASCVD** | 541 | 4,589 | -2.46 (-8.47,3.55) | -5.92 (-11.93,0.09) |
| | | | | | |
| **Whitehall II, DanMONICA, Framingham** | **ASCVD** | 364 | 3,645 | -4.14 (-11.97,3.69) | -8.16 (-15.99,-0.33) |

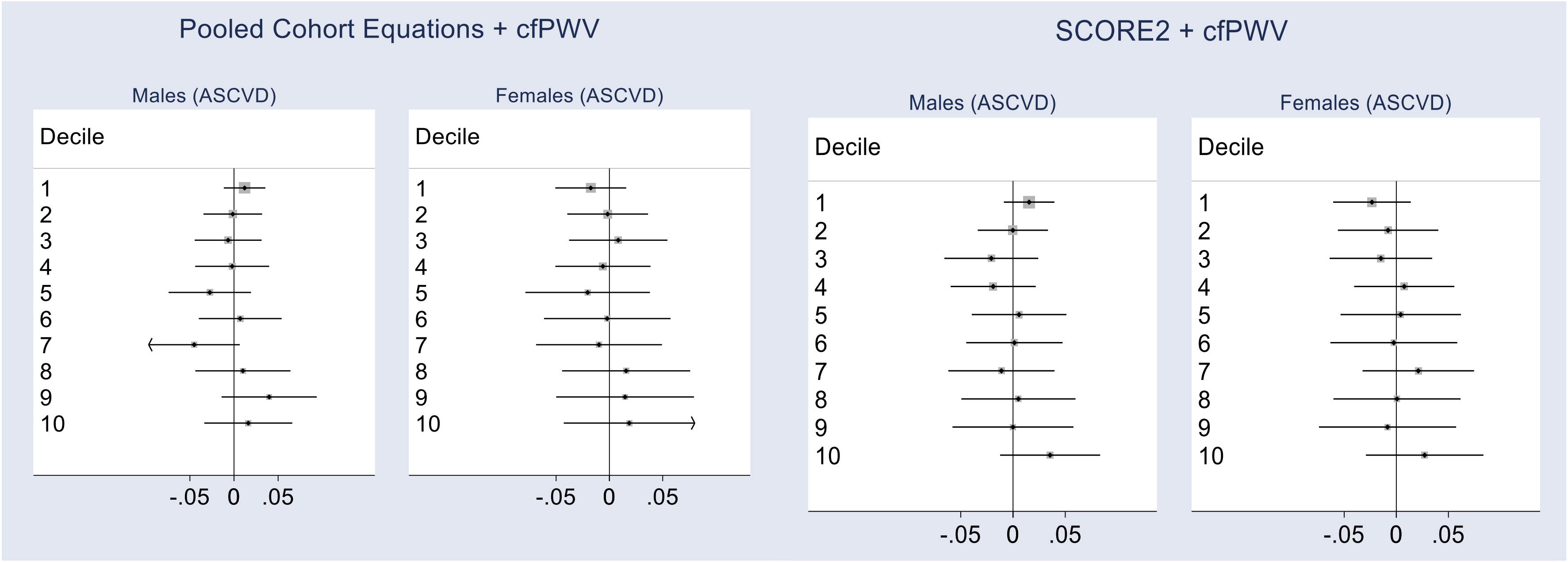


**Figure S11. Forest plot showing sex-specific pooled difference between predicted and observed risk of events at 10 years, study specific point estimates and confidence intervals for differences were calculated by taking 400 bootstrap samples and pooled in a 2-stage random effects meta-analysis.**

**Table S20. Hypothetical population of 100,000 middle-risk individuals based on the standard US population**

| Age category | US std pop (mil) | Scaled up to 1 million | Scaled to 100,000 |
|---|---|---|---|
| **40-49** | 153,969 | 387,862.498 | 38,786.250 |
| **50-59** | 111,170 | 280,047.762 | 28,004.776 |
| **60-69** | 73,057 | 184,037.504 | 18,403.75 |
| **70-79** | 58,772 | 148,052.236 | 14,805.22 |
| **Total:** | **396,968** | **1,000,000** | **100,000** |

**Table S21. Sex-specific proportions and numbers of individuals treated and untreated under the PCE's and the novel cfPWV model based on the US comparison.**

| | | Established PCE's | | Novel cfPWV model | |
|---|---|---|---|---|---|
| **Sex** | **Age category** | **Treated** | **Untreated** | **Treated** | **Untreated** |
| **Male** | **40-49** | 0.0108 (124.01) | 0.9892 (11,358.66) | 0.0108 (124.01) | 0.9892 (11,358.66) |
| | **50-59** | 0.0708 (468.32) | 0.9292 (6,146.41) | 0.1704 (1,127.15) | 0.8949 (5,487.58) |
| | **60-69** | 0.2414 (828.11) | 0.7586 (2,602.35) | 0.2471 (847.67) | 0.7529 (2,582.79) |
| | **70-79** | 0.5850 (1,441.63) | 0.4150 (1,022.70) | 0.6311 (1,555.24) | 0.3689 (909.09) |
| | **Totals:** | **0.1193 (2,862.08)** | **0.8807 (21,130.10)** | **0.1523 (3,654.07)** | **0.8477 (20,338.12)** |
| | | | | | |
| **Female** | **40-49** | 0.0183 (210.13) | 0.9817 (11,272.54) | 0.0000 (0.00) | 1.0000 (11,482.67) |
| | **50-59** | 0.0162 (107.16) | 0.9838 (6,507.57) | 0.0097 (64.16) | 0.9903 (6,550.57) |
| | **60-69** | 0.1394 (478.21) | 0.8606 (2,952.25) | 0.0876 (300.51) | 0.9124 (3,129.95) |
| | **70-79** | 0.3874 (954.68) | 0.6126 (1,509.65) | 0.5195 (1,280.22) | 0.4805 (1,184.11) |
| | **Totals:** | **0.0729 (1,750.18)** | **0.9271 (22,242.01)** | **0.0686 (1,644.89)** | **0.9314 (22,347.30)** |
| | | | | | |
| | **TOTAL:** | **0.0961 (4,612.26)** | **0.9039 (43,372.11)** | **0.1104 (5,298.96)** | **0.8896 (42,685.42)** |

**Table S22. Hypothetical population of 100,000 middle-risk individuals based on the standard European population for ages 40-69 years.**

| Age category | European standard population (100,000) | Scaled up to 100,000 |
|---|---|---|
| **40-49** | 14,000 | 35,898 |
| **50-59** | 13,500 | 34,615 |
| **60-69** | 11,500 | 29,487 |
| **Total:** | **48,000** | **1,000,000** |

**Table S23. Sex-specific proportions and numbers of individuals treated under SCORE2 and the novel cfPWV model based on the European comparison.**

| | | Established SCORE model | Novel cfPWV model |
|---|---|---|---|

| Sex | Age category | Treated | Untreated | Treated | Untreated |
|---|---|---|---|---|---|
| **Male** | **40-49** | 0.1549 (2,780) | 0.8451 (15,169) | 0.2794 (5,015) | 0.7206 (12,934) |
| | **50-59** | 0.2922 (5,057) | 0.7078 (12,251) | 0.3843 (6,651) | 0.6157 (10,657) |
| | **60-69** | 0.5110 (7,534) | 0.4890 (7,209) | 0.4365 (6,435) | 0.5635 (8,308) |
| | **Totals:** | **0.3074 (15,371)** | **0.6926 (34,629)** | **0.3620 (18,101)** | **0.6380 (31,899)** |
| | | | | | |
| **Female** | **40-49** | 0.0477 (857) | 0.9523 (17,092) | 0.0860 (1,544) | 0.9140 (16,405) |
| | **50-59** | 0.0333 (576) | 0.9667 (16,732) | 0.0997 (1,725) | 0.9003 (15,583) |
| | **60-69** | 0.1728 (2,548) | 0.8272 (12,195) | 0.2607 (3,844) | 0.7393 (10,899) |
| | **Totals:** | **0.0796 (3,981)** | **0.9204 (46,019)** | **0.1423 (7,113)** | **0.8577 (42,887)** |
| | | | | | |
| | **TOTAL:** | **0.1935 (19,352)** | **0.8065 (80,648)** | **0.2521 (25,214)** | **0.7479 (74,786)** |

**Table S24. Hypothetical population of 100,000 middle-risk individuals based on the standard European population for ages 40-79 years.**

| Age category | European standard population (100,000) | Scaled up to 1,000,000 |
|---|---|---|
| **40-49** | 14,000 | 29,167 |
| **50-59** | 13,500 | 28,125 |
| **60-69** | 11,500 | 23,958 |
| **70-79** | 9,000 | 18,750 |
| **Total:** | **48,000** | **1,000,000** |

**Table S25. Sex-specific proportions and numbers of individuals treated under SCORE2/SCORE2-OP and the novel cfPWV model based on the European comparison.**

| | | Established SCORE model | | Novel cfPWV model | |
|---|---|---|---|---|---|
| **Sex** | **Age category** | **Treated** | **Untreated** | **Treated** | **Untreated** |
| **Male** | **40-49** | 0.1549 (2,258) | 0.8451 (12,325) | 0.2794 (4,075) | 0.7206 (10,509) |
| | **50-59** | 0.2921 (4,108) | 0.7079 (9,954) | 0.3843 (5,404) | 0.6157 (8,658) |
| | **60-69** | 0.5111 (6,122) | 0.4889 (5,856) | 0.4365 (5,228) | 0.5635 (6,750) |
| | **70-79** | 0.9984 (9,360) | 0.0016 (15) | 0.9287 (8,707) | 0.0713 (668) |
| | **Totals:** | **0.4370 (21,848)** | **0.5630 (28,150)** | **0.4683 (23,413)** | **0.5317 (26,585)** |
| | | | | | |
| **Female** | **40-49** | 0.0797 (697) | 0.9203 (8,049) | 0.1539 (1,346) | 0.8461 (7,399) |
| | **50-59** | 0.0333 (468) | 0.9667 (13,595) | 0.1016 (1,429) | 0.8984 (12,633) |
| | **60-69** | 0.1729 (2,071) | 0.8271 (9,907) | 0.2557 (3,062) | 0.7443 (8,915) |
| | **70-79** | 0.3189 (2,990) | 0.6811 (6,385) | (0.0000) 0 | (1.0000) 9,375 |
| | **Totals:** | **0.1410 (6,225)** | **0.8590 (37,935)** | **0.1322 (5,838)** | **0.8678 (38,323)** |
| | | | | | |

| | | | | | |
|---|---|---|---|---|---|
| | **TOTAL:** | **0.2982 (28,074)** | **0.7018 (66,086)** | **0.3120 (29,374)** | **0.6880 (64,785)** |

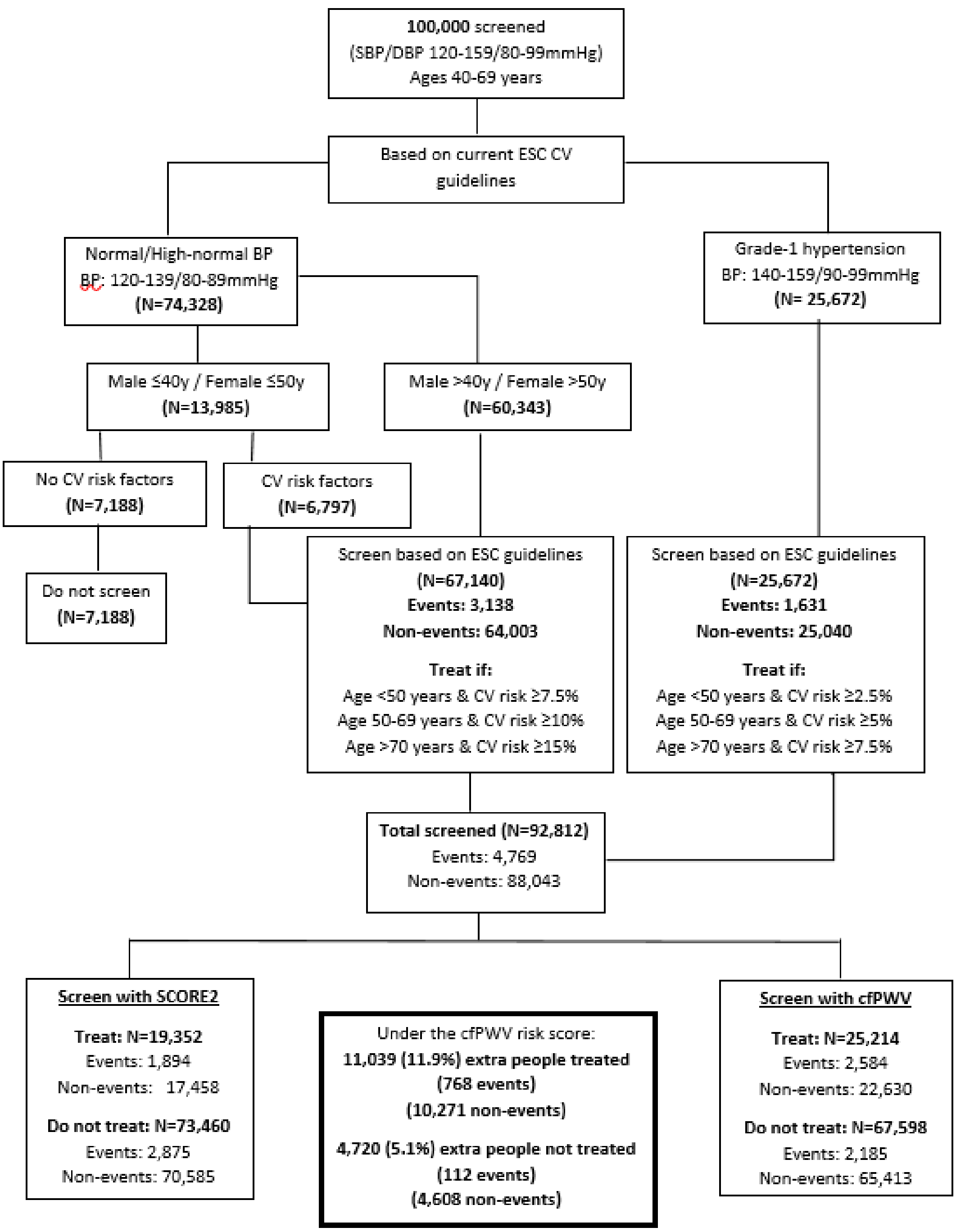


**Figure S12. Flow chart showing the screening and treatment pathway for a hypothetical population of 100,000 individuals aged 40-69 years based on the SCORE2 risk model and the novel cfPWV risk model.**

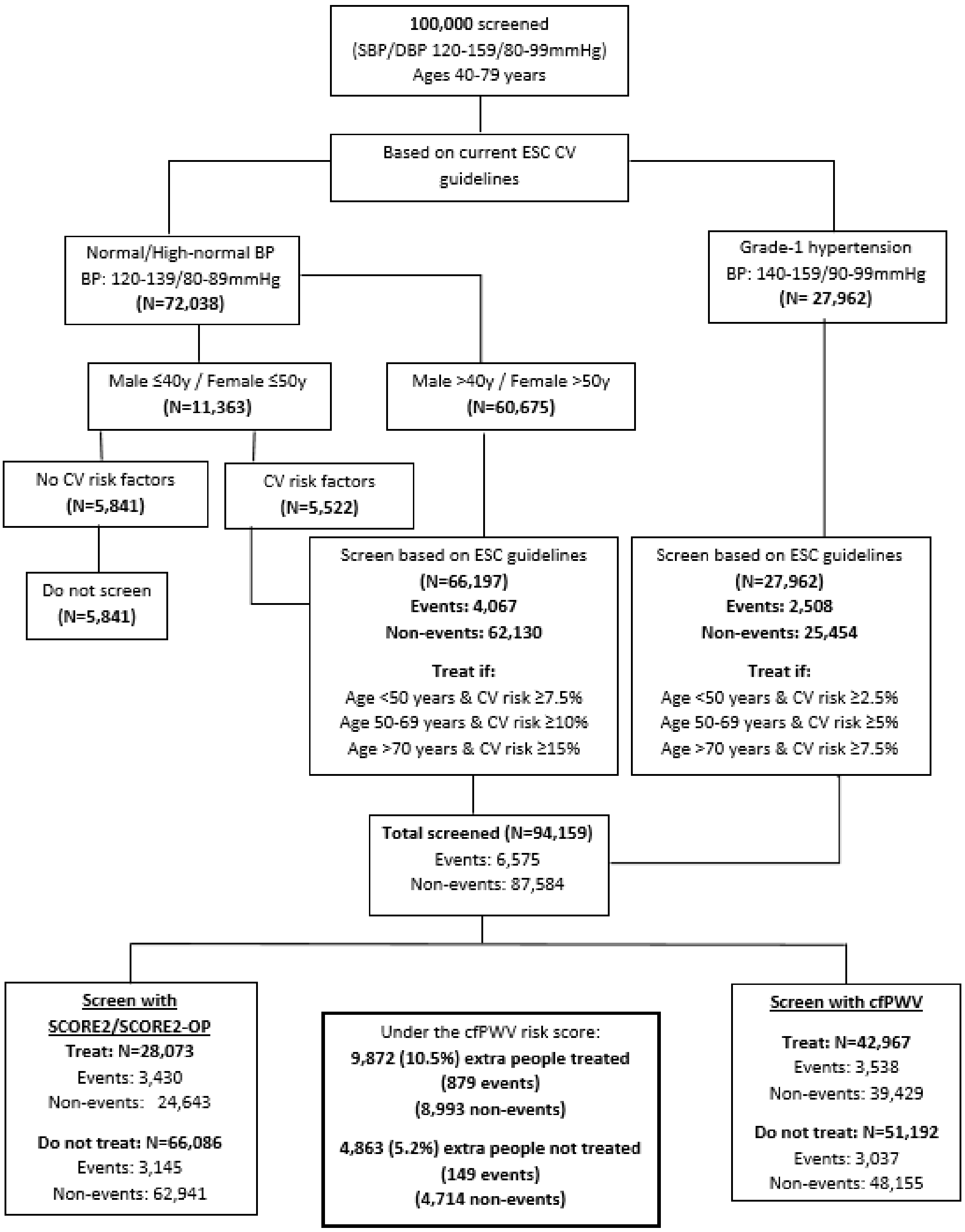


**Figure S13. Flow chart showing the screening and treatment pathway for a hypothetical population of 100,000 individuals aged 40-79 years based on the SCORE2/SCORE2-OP risk model and the novel cfPWV risk model.**

**Table S26. Summary of public health modelling measures if the novel cfPWV risk models were used instead of the established risk models.**

| | | Model compared to cfPWV equation (age range (years)) | | |
|---|---|---|---|---|
| | | **PCE (40-79)[1]** | **SCORE2/ SCORE2-OP[3] (40-79)** | **SCORE2 (40-69)[4]** |
| **Number screened (/100,000)** | | 47,984 | 94,159 | 92,812 |
| | | | | |
| **Additional treated (/screened)** | **Total** | 2,876 (6.0%) | 9,872 (10.5%) | 11,039 (11.9%) |
| | **Events** | 407 (14.2%) | 879 (8.9%) | 768 (7.0%) |
| | | | | |
| **Additional untreated (/screened)** | **Total** | 1,978 (4.1%) | 4,863 (5.2%) | 4,720 (5.1%) |
| | **Events** | 179 (9.0%) | 149 (3.1%) | 112 (2.4%) |
| | | | | |
| **Change in NNS ($NNS_{cfPWV\ model}$ – $NNS_{established\ model}$)[5]** | | -72 (244-316) | -4 (133-137) | -65 (180-245) |
| **Change in NNT ($NNT_{cfPWV\ model}$ – $NNT_{established\ model}$)[6]** | | -4 (27-31) | +20 (61-41) | -2 (49-51) |
| | | | | |
| **Risk reduction (%)** | | 1.7 | 0.34 | 2.9 |

[1]Based on the AHA/ACC guidelines in the US, individuals with a SBP/DBP <130/80mmHg were not eligible for antihypertensive treatment and individuals with a SBP/DBP ≥140/90mmHg were eligible for treatment. Individuals in the middle risk group were eligible for treatment only if their predicted 10-year ASCVD risk ≥10%. Of the 47,984 individuals screened, the addition of cfPWV to risk models reclassified 2,876 (6.0%) as eligible for treatment, 407 (14.2%) of whom would be expected to have an ASCVD event within 10 years. Compared to the current guidelines, if the novel cfPWV risk model was used, 72 less people would need to be screened to prevent one additional event, of whom 4 less people would be treated, and the overall event rate reduced by approximately 1.7%.

[2]Based on the ESC guidelines Individuals with normal/high-normal BP (SBP/DBP: 120-139/80-89mmHg) are screened if they are over 40 years and male, over 50 years and female or if they have any CV risk factors. Of the individuals who are screened, treatment initiation is guided by BP and age-specific thresholds of 10-year predicted risk. For those with grade-1 hypertension, treatment is initiated if their 10-year predicted risk is high or very high. For those with normal/high-normal BP, treatment is initiated if their predicted risk is very high. The SCORE2 model was used for individuals aged ≥70 years to allow direct comparisons to the derived novel risk score. Of the 94,159 individuals screened, the addition of cfPWV to risk models reclassified 9,872 (10.5%) as eligible for treatment, 879 (8.9%) of whom would be expected to have an ASCVD event within 10 years. Compared to the current guidelines, if the novel cfPWV risk model was used, 4 less people would need to be screened to prevent one additional event, but 20 more people would be treated, and the overall event rate reduced by approximately 0.34%.

[3]As guided by the ESC, the SCORE2-OP model was used for individuals aged ≥70 years and compared this to the novel European ASCVD risk model. Of the 92,812 individuals screened, the addition of cfPWV to risk models reclassified 11,039 (11.9%) as eligible for treatment, 768 (7.0%) of whom would be expected to have an ASCVD event within 10 years. Compared to the current guidelines, if the novel cfPWV risk model was used, 65 less people would need to be screened to prevent one additional event, and 2 less people would be treated, and the overall event rate reduced by approximately 2.9%.

[4]The ESC recommends that the SCORE2 equation is used for individuals aged 40-69 years.

[5]Change in the number needed to screen to prevent one event from the established risk model

[6]Change in the number needed to treat to prevent one event from the established risk model

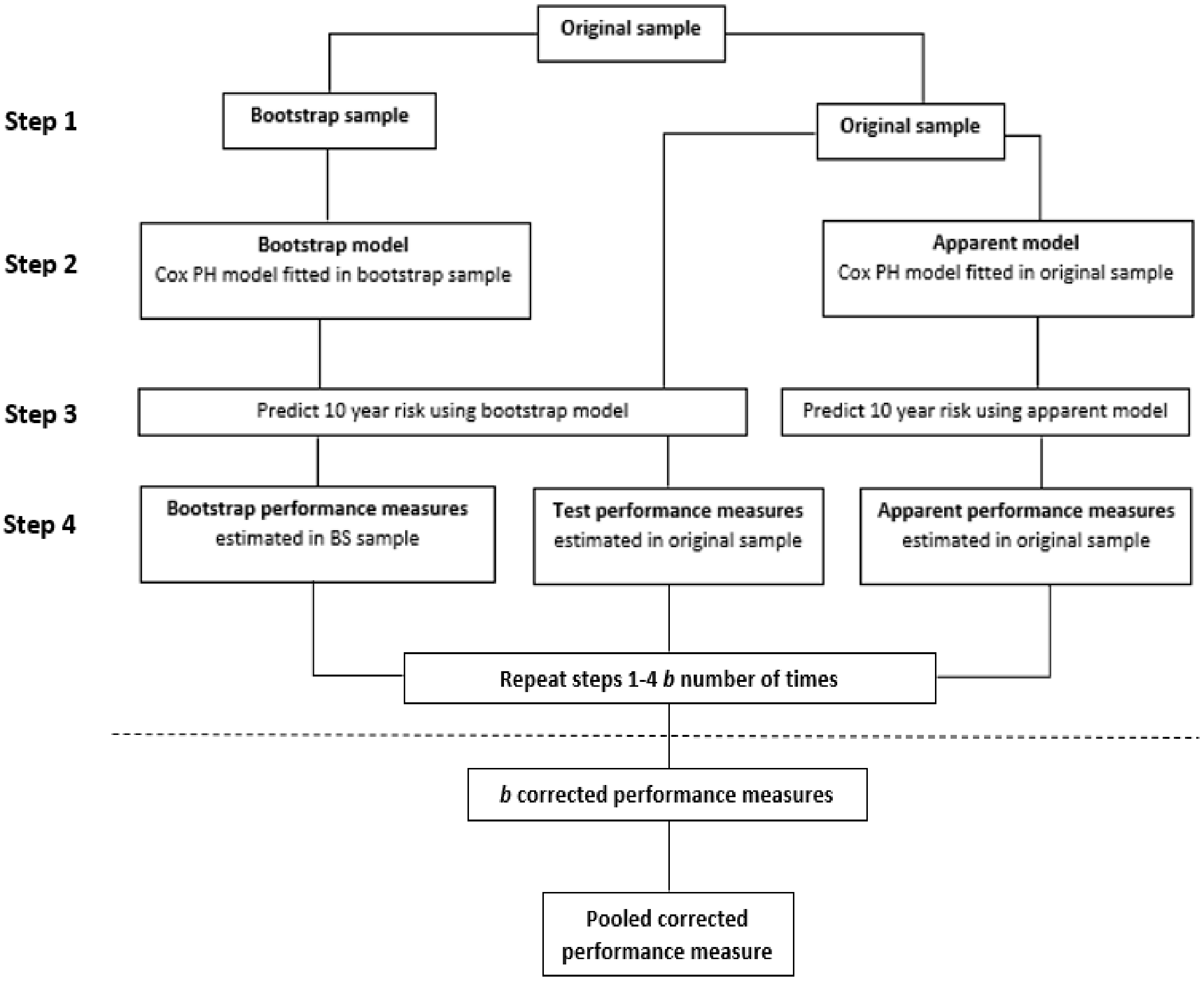


**Figure S14 – Flow chart of Harrell's bias correction method of bootstrap-based correction**
