# Supplementary material for: Association between carotid-femoral pulse wave velocity and cardiovascular disease in individuals with moderate blood pressure: a systematic review and individual participant meta-analysis
Source: BMJ Open. 2025 Dec 15;15(12):e101368. doi: 10.1136/bmjopen-2025-101368 (PMC12706244; doi:10.1136/bmjopen-2025-101368)
Supplement: online supplemental file 3 [file bmjopen-15-12-s003.pptx]

## Slide 1
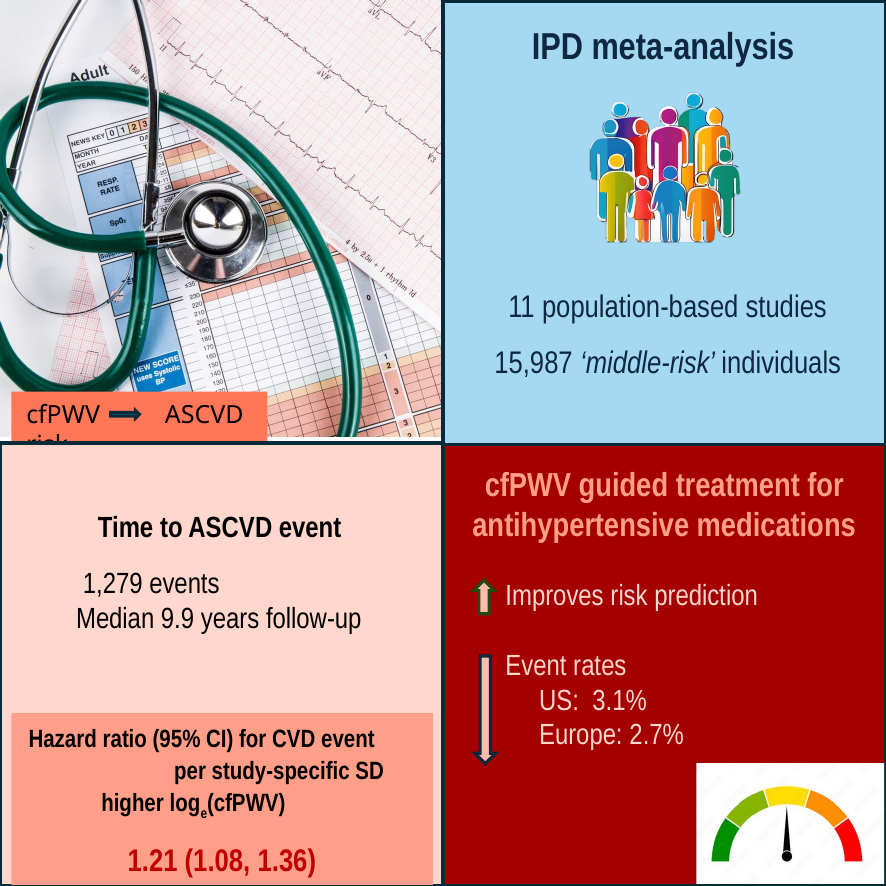

IPD meta-analysis
11 population-based studies 15,987 ‘middle-risk’ individuals
cfPWV ASCVD risk
cfPWV guided treatment for antihypertensive medications
Time to ASCVD event
1,279 events Median 9.9 years follow-up
Improves risk prediction
Event rates
 US: 3.1%
 Europe: 2.7%
Hazard ratio (95% CI) for CVD event per study-specific SD higher loge(cfPWV)
1.21 (1.08, 1.36)
