# Supplementary material for: Association between carotid-femoral pulse wave velocity and cardiovascular disease in individuals with moderate blood pressure: a systematic review and individual participant meta-analysis
Source: BMJ Open. 2025 Dec 15;15(12):e101368. doi: 10.1136/bmjopen-2025-101368 (PMC12706244; doi:10.1136/bmjopen-2025-101368)
Supplement: Supplementary file 4 [file bmjopen-2025-101368.reviewer_comments.pdf]

PEER REVIEW HISTORY

BMJ Open publishes all reviews undertaken for accepted manuscripts. Reviewers are asked to complete a checklist review form and are provided with free text boxes to elaborate on their assessment. These free text comments are reproduced below.

ARTICLE DETAILS

Title (Provisional)

Association between carotid-Femoral Pulse Wave Velocity and cardiovascular disease in individuals with moderate blood pressure: a systematic review and individual participant meta-analysis

Authors

Pavey, Holly; Wood, Angela; Mceniery, Carmel M.; AlGhatrif, Majd; Arshi, Banafsheh; Brunner, Eric; Chen, Chen-Huan; Cheng, Hao-Min; Hansen, Tine W.; Ikram, M Kamram; Kavousi, Maryam; Kuh, Diana; Kuipers, Allison L; Lakatta, Edward G.; linneberg, allan; Mattace Raso, Francesco; Mitchell, Gary F. F.; Maldonado, João; Newman, Anne B; Pereira, Telmo; Maki-Petaja, Kaisa; Shipley, Martin; Vasan, Ramachandran S.; Wong, Andrew; Ben-Shlomo, Yoav; Wilkinson, Ian B

VERSION 1 - REVIEW

**Reviewer**	**1**
**Name**	**Cavero-Redondo, Iván**
**Affiliation**	**Universidad de Castilla-La Mancha, Health and Social Research Center**
**Date**	**10-Apr-2025**
**COI**	**None**

The manuscript titled "Association between carotid-femoral Pulse Wave Velocity and cardiovascular disease in individuals with moderate blood pressure: an individual participant meta-analysis" addresses a relevant clinical question and is methodologically well executed. The use of harmonized individual participant data (IPD) and the integration of cfPWV into established cardiovascular risk models represent notable strengths. However, several issues require clarification or further development to enhance the interpretability and applicability of the findings.

The study provides a solid justification for focusing on cfPWV in individuals with moderate blood pressure. That said, the conceptual framework could benefit from a deeper discussion of the role of arterial stiffness not only as a marker of vascular damage, but also as a potential early mechanism driving blood pressure elevation and cardiovascular risk. There is

increasing evidence supporting the predictive value of cfPWV even in normotensive individuals. In this context, the systematic review by Saz-Lara et al. (Front Cardiovasc Med. 2022;9:798934) may offer a relevant perspective.

A critical issue that remains insufficiently addressed is the handling of participants under antihypertensive treatment. The manuscript should clarify whether such individuals were excluded, and if included, how treatment status was incorporated into the analysis. This is particularly relevant given the growing body of evidence suggesting that different antihypertensive drug classes exert heterogeneous effects on arterial stiffness. Studies such as those by Cavero-Redondo et al. (Front Pharmacol. 2023;13:1225795) and Cavero-Redondo et al. (J Med Internet Res. 2024;26:e54357) explore the comparative effects of pharmacological interventions on cfPWV and propose treatment optimization strategies using machine learning methods. While direct inclusion of these references is not essential, the authors may find them useful when interpreting the potential influence of pharmacologic treatment on their primary exposure variable.

The statistical modeling strategy is appropriate, and the integration of cfPWV into SCORE2, SCORE2-OP, and PCE is well conceived. Nonetheless, further clarification is needed regarding the recalibration of these models. A concise description in the main text of whether baseline hazards or coefficients were modified would improve transparency.

Additionally, the modest increase in the C-index after including cfPWV calls for a more critical discussion of its practical implications. The authors are encouraged to reflect on whether these changes would alter risk classification thresholds in clinical settings. Use of complementary indices such as the net reclassification improvement (NRI) or decision curve analysis could provide additional insight, should data allow.

Given the inclusion of multiple European cohorts, some discussion of heterogeneity in baseline characteristics, outcome definitions, or cfPWV measurement protocols would be useful. Was any heterogeneity observed or tested statistically? Furthermore, stratified analyses by sex and age could be valuable, particularly considering established differences in vascular aging dynamics across demographic subgroups.

The potential role of cfPWV in guiding preventive decisions in individuals with moderate blood pressure is an interesting angle. To improve this section, the authors may wish to discuss how this approach aligns with or diverges from recent clinical guidelines (e.g., ESC/ESH 2021, ACC/AHA 2017), which still list cfPWV as an optional or secondary biomarker. Greater emphasis on how the current findings might inform or refine these recommendations would strengthen the manuscript.

The simulation model adds value to the translational perspective, but a clearer description of its assumptions—including intervention effect sizes and event probabilities—would enhance its interpretability.

Figures 2 and 3 effectively summarize key results but would benefit from clearer labeling to distinguish between primary and sensitivity analyses. Likewise, some of the information contained in the Supplement (e.g., variable harmonization procedures, handling of missing data, assumptions in predictive modeling) could be briefly summarized in the main text to improve accessibility.

The manuscript engages well with the literature, but the discussion could be further enriched by incorporating or considering recent studies that explore cfPWV as a modifiable risk factor and its integration into early risk stratification strategies. In addition to those previously mentioned, the following works may also provide valuable context:

• Vlachopoulos C, Aznaouridis K, Stefanadis C. Prediction of cardiovascular events and all-cause mortality with arterial stiffness: a systematic review and meta-analysis. J Am Coll Cardiol. 2010 Mar 30;55(13):1318-27. doi: 10.1016/j.jacc.2009.10.061. PMID: 20338492.

• Sequí-Domínguez I, Cavero-Redondo I, Álvarez-Bueno C, Pozuelo-Carrascosa DP, Nuñez de Arenas-Arroyo S, Martínez-Vizcaíno V. Accuracy of Pulse Wave Velocity Predicting Cardiovascular and All-Cause Mortality. A Systematic Review and Meta-Analysis. J Clin Med. 2020 Jul 2;9(7):2080. doi: 10.3390/jcm9072080. PMID: 32630671; PMCID: PMC7408852.

• Ben-Shlomo Y, et al. Aortic pulse wave velocity improves cardiovascular event prediction: an individual participant meta-analysis of prospective observational data from 17,635 subjects. J Am Coll Cardiol. 2014;63(7):636–646.

**Reviewer**	**2**
**Name**	**Apostolos, Anastasios**
**Affiliation**	**University of Athens, First Department of Cardiology**
**Date**	**21-Aug-2025**
**COI**	**None**

Dear Authors,

I have read with great interest your article entitled “Association between Carotid-Femoral Pulse Wave Velocity and Cardiovascular Disease in Individuals with Moderate Blood Pressure: An Individual Participant Meta-Analysis" submitted to BMJ Open. This original research article presents an IPD meta-analysis investigating the association between carotid-femoral pulse wave velocity (cfPWV)—a gold-standard measure of aortic stiffness—and cardiovascular disease (CVD) outcomes in individuals with moderate blood pressure (BP, likely defined as 120-139/80-89 mmHg per guidelines). Drawing from multiple cohorts across Europe, North America, and Asia (e.g., involving authors from Cambridge, Innsbruck, NIH, Erasmus MC, and others), the study pools participant-level data from longitudinal studies to

assess cfPWV as a predictor of incident CVD events (e.g., MI, stroke, heart failure). Methods likely include harmonized cfPWV measurements (e.g., tonometry or Doppler), multivariable Cox regression for hazard ratios, and subgroup analyses (e.g., by age, sex, BP strata). The manuscript emphasizes cfPWV's prognostic value beyond traditional risk factors in this "moderate" BP group, where guidelines debate intervention thresholds.While the manuscript is clinically relevant and well-written, it has several shortcomings. More specifically:

1. Heterogeneity in Measurements: cfPWV protocols vary (e.g., Complior vs. SphygmoCor devices), potentially introducing bias. Without the methods, it's unclear if harmonization (e.g., path length corrections) was adequate, as recommended in 2022 ARTERY Society guidelines.

2. Selection Bias: Cohorts may overrepresent Western populations (e.g., Framingham, Rotterdam), limiting generalizability to low/middle-income countries. This should be aknolwedged

3. Inclusion criteria for "moderate BP" (e.g., untreated vs. treated) and CVD events (e.g., fatal/non-fatal) need clarification.

4. If HRs are modest (e.g., 1.2-1.5 per SD cfPWV), clinical utility (e.g., reclassification improvement) needs evidence; otherwise, cfPWV's additive value over FRS/SCORE2 may be overstated.

5. A graphical abstract would improve the overall quality

**Reviewer**	**3**
**Name**	**Tan, Xianming**
**Affiliation**	**The University of North Carolina at Chapel Hill, Biostatistics**
**Date**	**29-Sep-2025**
**COI**	**None**

This is a well-written manuscript that addresses an important question on the added predictive value of carotid–femoral pulse wave velocity (cfPWV) for cardiovascular disease risk among individuals with moderate blood pressure. The large sample size, use of individual participant data meta-analysis, and careful modeling of predictive performance are major strengths. The statistical approach (Cox models within studies with random-effects pooling, evaluation of C-index and reclassification metrics) is appropriate and clearly presented.

That said, I have a few concerns regarding reporting and interpretation:

1. Between-study heterogeneity: Random-effects models were used, but heterogeneity statistics (e.g., I^2, tau^2) were not reported. This limits the reader's ability to assess variability across cohorts.

2. Missing data: Although both complete-case and imputed analyses were mentioned, the proportion and patterns of missingness were not described. This is important for evaluating potential bias.

3. Multiplicity: While ASCVD was clearly defined as the primary outcome, multiple secondary outcomes and subgroup analyses were presented without adjustments for multiplicity. These should be framed as exploratory or discussed with appropriate caution.

4. Observational design: The limitations inherent in observational data were not discussed. Even with large sample size and careful adjustment, causal interpretation is challenging if not impossible.

5. Registration of this study. It's now a common practice to register systematic review/meta-analysis studies.

Overall, I find the study rigorous and timely, but I recommend that the authors address these points through additional reporting and by tempering their conclusions.

VERSION 1 - AUTHOR RESPONSE

Reviewer: 1
Mr. Iván Cavero-Redondo, Universidad de Castilla-La Mancha
Comments to the Author:
The manuscript titled "Association between carotid-femoral Pulse Wave Velocity and cardiovascular disease in individuals with moderate blood pressure: an individual participant meta-analysis" addresses a relevant clinical question and is methodologically well executed. The use of harmonized individual participant data (IPD) and the integration of cfPWV into established cardiovascular risk models represent notable strengths. However, several issues require clarification or further development to enhance the interpretability and applicability of the findings.
The study provides a solid justification for focusing on cfPWV in individuals with moderate blood pressure.

1. That said, the conceptual framework could benefit from a deeper discussion of the role of arterial stiffness not only as a marker of vascular damage, but also as a potential early mechanism driving blood pressure elevation and cardiovascular risk. There is increasing evidence supporting the predictive value of cfPWV even in normotensive individuals. In this context, the systematic review by Saz-Lara et al. (Front Cardiovasc Med. 2022;9:798934) may offer a relevant perspective.

Thank you for providing this reference offering this perspective. A sentence and references were added into the introduction: “A few studies have shown that cfPWV predicted incident hypertension, even in normotensive individuals, suggesting that cfPWV may also act as a potential early mechanism driving blood pressure, and cardiovascular risk (4,5)”.

2. A critical issue that remains insufficiently addressed is the handling of participants under antihypertensive treatment. The manuscript should clarify whether such individuals were excluded, and if included, how treatment status was incorporated into the analysis. This is particularly relevant given the growing body of evidence suggesting that different antihypertensive drug classes exert heterogeneous effects on arterial stiffness. Studies such as those by Cavero-Redondo et al. (Front Pharmacol. 2023;13:1225795) and Cavero-Redondo et al. (J Med Internet Res. 2024;26:e54357) explore the comparative effects of pharmacological interventions on cfPWV and propose treatment optimization strategies using machine learning methods. While direct inclusion of these references is not essential, the authors may find them useful when interpreting the potential influence of pharmacologic treatment on their primary exposure variable.

   Thank you for this comment. I have added a sentence into the methods section to clarify this: “Individuals taking antihypertensive medications were included in this analysis, antihypertensive medication status was accounted for as a covariate in the fully adjusted models.”
   We have added a small paragraph to address this in the discussion, as well as acknowledging the limitation that we were not able to look at different medication classes in this analysis. “A final limitation is the lack of data on specific classes of antihypertensive medications. Although the SPARTE study showed no differential effects of drug classes on cfPWV (44), a meta-analysis looking at a broader medication range showed some evidence that certain drug classes may have differential effects on cfPWV beyond their blood pressure–lowering properties (45), the majority of cfPWV reduction is thought to be driven by reductions in blood pressure itself (46). Without class-specific treatment information, we were unable to account for potential variation in cfPWV response due to medication type.”

3. The statistical modeling strategy is appropriate, and the integration of cfPWV into SCORE2, SCORE2-OP, and PCE is well conceived. Nonetheless, further clarification is needed regarding the recalibration of these models. A concise description in the main text of whether baseline hazards or coefficients were modified would improve transparency.

Information regarding the recalibration can be found in the supplementary material. We have additionally updated the methods in the main manuscript to include a summary of this, along with two relevant references: “The PCE’s were recalibrated using calibration-in-the-large by adjusting the baseline survival with a uniform correction factor to reflect event rate differences (16). SCORE2 and SCORE2-OP were recalibrated to the ESC-defined low-risk region assigned to most Western European countries, assuming similar risk profiles in the US. This involved updating the baseline survival with age- and sex-specific CVD rates and mean risk factors, while keeping the original hazard ratios (13)”

4. Additionally, the modest increase in the C-index after including cfPWV calls for a more critical discussion of its practical implications. The authors are encouraged to reflect on whether these changes would alter risk classification thresholds in clinical settings. Use of complementary indices such as the net reclassification improvement (NRI) or decision curve analysis could provide additional insight, should data allow.

Thank you for this suggestion. We have added a small paragraph into the discussion to expand on this: “Although the addition of cfPWV to traditional risk models significantly improved discrimination, the gains were modest and may have limited clinical relevance. We reported the NRI due to its clinical interpretability and widespread use in risk prediction research; however, it did not reach statistical significance. In contrast, the IDI indicated improved sensitivity without increasing false positives. Additionally, population-level modelling suggested a small but tangible improvement in clinical utility when cfPWV was used to guide treatment decisions. Taken together, these findings raise important questions about whether incorporating cfPWV into risk assessment could influence clinical decision thresholds, particularly in individuals with moderate CV risk, based on blood pressure.”

Additionally, since the established CV risk factors are already very predictive, improving these becomes difficult, hence small (but significant) C-indices – this is a common finding when evaluating novel risk markers.

We appreciate the reviewer’s suggestion to consider Decision Curve Analysis (DCA) as a method to assess the clinical impact of adding cfPWV. While we did not perform formal DCA, we conducted a clinical utility analysis (population modelling) examining reclassification across established risk thresholds and the proportion of individuals appropriately reclassified based on actual event occurrence. This analysis provided insight into the real-world implications of cfPWV integration. Future work could benefit from employing decision curve analysis to evaluate whether incorporating cfPWV improves net clinical benefit across a range of decision thresholds, but we feel there are

already sufficient performance measures in this manuscript: C-index, IDI, NRI and population modelling.

5. Given the inclusion of multiple European cohorts, some discussion of heterogeneity in baseline characteristics, outcome definitions, or cfPWV measurement protocols would be useful. Was any heterogeneity observed or tested statistically? Furthermore, stratified analyses by sex and age could be valuable, particularly considering established differences in vascular aging dynamics across demographic subgroups.

We thank the reviewer for this helpful comment. All participating cohorts followed a common data request protocol specifying variables, cfPWV measurement methods, and outcomes of interest. However, some variability was expected due to differences in study design and population characteristics. Baseline characteristics are summarised in the second paragraph of the Results section. While we did not formally assess heterogeneity across cohorts, inspection of continuous baseline variables suggested reasonable consistency between studies. We acknowledge there was some variability in cfPWV values across cohorts; however, cfPWV was standardised within each study (mean-centred and scaled by the within-study standard deviation) prior to analysis. This standardisation facilitates comparability across cohorts and allows reliable pooling of effect estimates in the random-effects meta-analysis. Regarding heterogeneity in cfPWV measurement protocols, please see my response to reviewer 2, comment 1. Additionally, Ben-Shlomo et al. stated in their manuscript in 2013: "A sensitivity analysis examining the method used to calculate the distance for the carotid-femoral path failed to find any evidence of heterogeneity" (10.1016/j.jacc.2013.09.063).

I have included I-squared values and a p-value from Cochran's Q-statistic on the forest plots, showing how much heterogeneity there is and assessing whether heterogeneity is present, respectively. Between-cohort heterogeneity was generally modest (I^2 = 49% for ASCVD (the primary outcome), there was less heterogeneity for all CV events (I^2 = 38%). I have not added these to the tables since there are many exploratory endpoints, but I am happy to include this if the editor requests.

Supplementary table S6 summarises the cfPWV measurement methods (e.g., information on the waveform and how the distance was measured)

While we provided ICD-10 codes to guide harmonization of outcome definitions, several cohorts supplied cardiovascular endpoints as pre-defined within their original studies (e.g., "ASCVD," "CVD", "major cardiovascular events", "CHD" or "Stroke"), without detailed information on the exact derivation or component diagnoses. In such cases, we used the cohort-provided definitions as reported, acknowledging potential variability in endpoint ascertainment across studies. We summarized the requested outcome

definitions in Supplementary Table S5. A column has been added to this table to provide further detail on the endpoints.

We thank the reviewers for these considerations. While stratification by sex and age could provide valuable insights, given known differences in vascular aging, given that the thresholds for target cfPWV is stable across sex and age, we felt adjusting for these variables was sufficient. Perhaps a minor limitation, however we performed subgroup analyses by age group and sex and did not find any effect of heterogeneity, suggesting that the predictive value of cfPWV was consistent across demographic subgroups.

6. The potential role of cfPWV in guiding preventive decisions in individuals with moderate blood pressure is an interesting angle. To improve this section, the authors may wish to discuss how this approach aligns with or diverges from recent clinical guidelines (e.g., ESC/ESH 2021, ACC/AHA 2017), which still list cfPWV as an optional or secondary biomarker. Greater emphasis on how the current findings might inform or refine these recommendations would strengthen the manuscript.

We thank the reviewer for this valuable comment. We agree that contextualising our findings within current clinical guidelines enhances their relevance. ESC (2024 elevated blood pressure and hypertension) guidelines acknowledge cfPWV as an optional or secondary risk marker, particularly in borderline individuals. Our results suggest that cfPWV may improve risk stratification in this population and could inform treatment decisions by identifying those at higher long-term risk, thereby potentially lowering the number needed to treat (NNT). This aligns with the ESC view of cfPWV as a marker of hypertension-mediated organ damage, which may influence therapeutic decisions, particularly when treatment decisions are uncertain. This is especially relevant in individuals with moderate BP since, individuals with very high BP (160/100+mmHg) are at high risk and easier to treat, potentially even based on BP alone, but risk stratification in borderline (moderate BP) individuals may improve the NNT. We have added a paragraph to the discussion to address this point: “Our findings have relevance for individuals with moderate BP (120-159/80-99mmHg), a group for whom treatment decisions often rely on additional risk stratification. Recent ESC guidelines for the management of elevated blood pressure and hypertension (2024) have listed cfPWV as a secondary or optional marker, particularly in individuals where treatment remains uncertain. Notably, the ESC guidelines consider elevated cfPWV as evidence of hypertension-mediated organ damage, which may influence the decision to initiate pharmacological therapy. In our population modelling, the use of cfPWV to guide treatment in this group led to a modest reduction in ASCVD event rates and improved targeting of therapy. These findings suggest that cfPWV could enhance decision-making in clinical grey zones, helping to identify patients more likely to benefit from early

intervention, and potentially supporting shared decision-making by providing a clearer picture of individual risk".

We have also added a sentence into the introduction to expand on this.

7. The simulation model adds value to the translational perspective, but a clearer description of its assumptions—including intervention effect sizes and event probabilities—would enhance its interpretability. Figures 2 and 3 effectively summarize key results but would benefit from clearer labeling to distinguish between primary and sensitivity analyses.

We have added some extra detail to the methods section: "The model assumed age- and sex-specific ASCVD event rates based on observed cohort data, standard population distributions (US or European). Additionally, it was assumed that treatment with antihypertensive medications was allocated per current guidelines and reduced the risk of ASCVD by 20% (28,29)."

The legends for figures 2 and 3 have been updated to make it clearer in the tables, which I hope will clear up any confusion.

8. Likewise, some of the information contained in the Supplement (e.g., variable harmonization procedures, handling of missing data, assumptions in predictive modeling) could be briefly summarized in the main text to improve accessibility.

Please see my response to reviewer 2's first comment regarding the harmonisation of cfPWV. Additionally, I have added some further detail into the statistical analysis section of the methods in the manuscript: "Due to positive skewness and to account for heterogeneity in cfPWV study protocols across cohorts, cfPWV values were $\log_e$-transformed and standardized within each study (z-score). This within-study standardization allowed harmonization of cfPWV values across cohorts by placing them on a comparable relative scale, thereby enabling pooled analyses. Effect estimates therefore represent the change in risk of the outcome per one SD higher log_e-transformed cfPWV within each study".

I have added further detail in the methods section of the manuscript regarding model checks and assumptions for the predictive models: "Funnel plots and Egger's tests were used to assess potential small-study effects or selection bias. Linearity of the association between standardized log(cfPWV) and outcomes in Cox proportional hazards models was assessed via martingale residual plots, and the proportional hazards assumption was evaluated using Schoenfeld residuals plotted against time." I have also added results of these into the results section of the manuscript, referencing relevant figures and tables in the supplementary material: "Funnel plots and Egger's tests showed no evidence of small-study effects or selection bias. Martingale residual

plots suggested that the association between standardized log(cfPWV) and outcomes was approximately linear, and Schoenfeld residuals supported the proportional hazards assumption (Figures S3-S6)".

Additionally, further information has been provided in the methods section of the manuscript regarding multiple imputation: "Missing individual-level data were imputed using the jomo package with a random-effects model, including event outcomes and the Nelson-Aalen estimator of survival time. Participants with missing outcomes or follow-up were censored at their last available examination date. Further details can be found in Supplementary methods 1".

9. The manuscript engages well with the literature, but the discussion could be further enriched by incorporating or considering recent studies that explore cfPWV as a modifiable risk factor and its integration into early risk stratification strategies. In addition to those previously mentioned, the following works may also provide valuable context:
   • Vlachopoulos C, Aznaouridis K, Stefanadis C. Prediction of cardiovascular events and all-cause mortality with arterial stiffness: a systematic review and meta-analysis. J Am Coll Cardiol. 2010 Mar 30;55(13):1318-27. doi: 10.1016/j.jacc.2009.10.061. PMID: 20338492.
   • Sequí-Domínguez I, Cavero-Redondo I, Álvarez-Bueno C, Pozuelo-Carrascosa DP, Nuñez de Arenas-Arroyo S, Martínez-Vizcaíno V. Accuracy of Pulse Wave Velocity Predicting Cardiovascular and All-Cause Mortality. A Systematic Review and Meta-Analysis. J Clin Med. 2020 Jul 2;9(7):2080. doi: 10.3390/jcm9072080. PMID: 32630671; PMCID: PMC7408852.
   • Ben-Shlomo Y, et al. Aortic pulse wave velocity improves cardiovascular event prediction: an individual participant meta-analysis of prospective observational data from 17,635 subjects. J Am Coll Cardiol. 2014;63(7):636–646.

Thank you for the suggestion of additional references. Ben-Shlomo et al. has already been cited and discussed in the manuscript, as it particularly motivates our focus on the moderate-risk population. We have also added references to the other two meta-analyses (Vlachopoulos et al., 2010; Sequí-Domínguez et al., 2020) to provide a brief background on previous work and the prognostic value of cfPWV in cardiovascular risk prediction. Importantly, our results align with other meta-analyses.

Reviewer: 2
Dr. Anastasios Apostolos, University of Athens
Comments to the Author:
Dear Authors,
I have read with great interest your article entitled "Association between Carotid-Femoral Pulse Wave Velocity and Cardiovascular Disease in Individuals with Moderate Blood Pressure: An Individual Participant Meta-Analysis" submitted to BMJ Open. This

original research article presents an IPD meta-analysis investigating the association between carotid-femoral pulse wave velocity (cfPWV)—a gold-standard measure of aortic stiffness—and cardiovascular disease (CVD) outcomes in individuals with moderate blood pressure (BP, likely defined as 120-139/80-89 mmHg per guidelines). Drawing from multiple cohorts across Europe, North America, and Asia (e.g., involving authors from Cambridge, Innsbruck, NIH, Erasmus MC, and others), the study pools participant-level data from longitudinal studies to assess cfPWV as a predictor of incident CVD events (e.g., MI, stroke, heart failure). Methods likely include harmonized cfPWV measurements (e.g., tonometry or Doppler), multivariable Cox regression for hazard ratios, and subgroup analyses (e.g., by age, sex, BP strata). The manuscript emphasizes cfPWV's prognostic value beyond traditional risk factors in this "moderate" BP group, where guidelines debate intervention thresholds.While the manuscript is clinically relevant and well-written, it has several shortcomings. More specifically:
1. Heterogeneity in Measurements: cfPWV protocols vary (e.g., Complior vs. SphygmoCor devices), potentially introducing bias. Without the methods, it's unclear if harmonization (e.g., path length corrections) was adequate, as recommended in 2022 ARTERY Society guidelines.

We acknowledge the concern regarding heterogeneity in cfPWV measurement protocols across studies, particularly differences between devices such as Complior and SphygmoCor, and the implications for path length correction. We have provided a table outlining the devices and measurement protocols used for waveform and distance in each contributing study (see Table S6), offering transparency around potential variation. Additionally, we have standardized hazard ratios within each study, presenting associations per study-specific standard deviation of cfPWV. This statistical approach removes the influence of between-study differences in measurement scale or absolute cfPWV values, and allows for comparable effect estimates across cohorts, irrespective of the device used. We hope that this satisfied this concern. This standardization is a well-established method for harmonizing across heterogeneous measurement protocols in individual participant data (IPD) meta-analyses. It ensures that the resulting pooled estimates reflect the relative risk per unit increase in cfPWV within each study population, rather than being biased by systematic differences in cfPWV levels due to device-specific calibration or path length assumptions. While the 2022 ARTERY Society guidelines recommend harmonization of measurement protocols, in IPD settings where retrospective harmonization is not always possible, statistical standardization as applied here represents a valid and effective alternative.

2. Selection Bias: Cohorts may overrepresent Western populations (e.g.,

Framingham, Rotterdam), limiting generalizability to low/middle-income countries. This should be aknolwedged.

Thank you for raising this limitation that indeed should be acknowledged. I have added a sentence into the limitations section of the discussion: “Finally, most included studies involved individuals of white ethnicity may overrepresent Western populations’ generalising findings to other ethnic groups and low/middle-income populations requires caution.”

3. Inclusion criteria for "moderate BP" (e.g., untreated vs. treated) and CVD events (e.g., fatal/non-fatal) need clarification.

Thank you for suggesting that further clarification on defining the inclusion criteria and outcomes would be useful. We have updated table S5 to include a description of each endpoint and added some detail into the manuscript: “The primary outcome measure was atherosclerotic CVD (ASCVD) (including all stroke events, fatal CHD (ischemic heart disease) and non-fatal myocardial infarction)”. I have included further detail about how fatal/non-fatal events were established: “Fatal events were defined by each study according to their own criteria, typically based on death certificates, hospital records, or adjudication by study investigators. We used these study-designated classifications to harmonize fatal and nonfatal outcomes across cohorts”.

Additionally, methods section of the manuscript has been edited to provide clarification regarding the inclusion criteria for moderate BP: “Anonymised individual-level data, including cardiovascular risk factors and outcomes, were requested from each study (see Supplementary Material). Individuals with moderate blood pressure (defined throughout as: SBP 120–159 mmHg or DBP 80–99 mmHg) were included in the analysis. There were no exclusions based on antihypertensive medication use, but medication use was adjusted for in all analyses. Individuals with a history of cardiovascular disease (CVD), SBP <120 mmHg or DBP <80 mmHg, or SBP ≥160 mmHg or DBP ≥100 mmHg were excluded. Further details are provided in Supplementary Methods 1”.

4. If HRs are modest (e.g., 1.2-1.5 per SD cfPWV), clinical utility (e.g., reclassification improvement) needs evidence; otherwise, cfPWV's additive value over FRS/SCORE2 may be overstated.

We thank the reviewer for raising the important point regarding the clinical utility of cfPWV and its potential incremental value over established risk scores such as the PCEs or SCORE2.

We assessed the improvement in risk prediction using both the NRI and the Integrated Discrimination Improvement (IDI). While the NRI was not statistically

significant, the IDI indicated a modest but statistically significant improvement in discrimination when cfPWV was added to the base risk model.
These findings suggest that cfPWV may offer some incremental predictive value, particularly in terms of improving average sensitivity for detecting individuals who develop CVD. However, the lack of significant reclassification underscores the need for further research to determine the clinical utility of cfPWV in guiding decision-making, especially in intermediate-risk populations.
We have added these results to the Results and Discussion sections of the manuscript and have noted this as a limitation regarding cfPWV's current applicability in clinical risk prediction frameworks.

5. A graphical abstract would improve the overall quality
Thank you for this suggestion – we agree and have uploaded a graphical abstract with the updated submission.

Reviewer: 3
Dr. Xianming Tan, The University of North Carolina at Chapel Hill
Comments to the Author:
This is a well-written manuscript that addresses an important question on the added predictive value of carotid–femoral pulse wave velocity (cfPWV) for cardiovascular disease risk among individuals with moderate blood pressure. The large sample size, use of individual participant data meta-analysis, and careful modeling of predictive performance are major strengths. The statistical approach (Cox models within studies with random-effects pooling, evaluation of C-index and reclassification metrics) is appropriate and clearly presented.
That said, I have a few concerns regarding reporting and interpretation:

1. Between-study heterogeneity: Random-effects models were used, but heterogeneity statistics (e.g., I^2, tau^2) were not reported. This limits the reader's ability to assess variability across cohorts.
Thank you for this comment, I have now added I-squared and Q statistics to the forest plots for the primary analysis (ASCVD), and for all CV events. I have included I-squared values and a p-value from Cochran's Q-statistic on the forest plots, showing how much heterogeneity there is and assessing whether heterogeneity is present, respectively. Between-cohort heterogeneity was generally modest (I^2 = 49% for ASCVD (the primary outcome), there was less heterogeneity for all CV events (I^2 = 38%). I have not added these to the tables since there are many exploratory endpoints, but I am happy to include this if the editor requests.

2. Missing data: Although both complete-case and imputed analyses were mentioned, the proportion and patterns of missingness were not described. This is important for evaluating potential bias.

For 10 studies providing individual participant data, missing cfPWV data were present in some studies. In some datasets, only participants with cfPWV were provided; in others, all participants were included and missing cfPWV was identifiable. Therefore, estimates of missingness are incomplete and should be interpreted with caution. Please see the table below showing proportions of missingness in each study that I had IPD for.

	N	cfPWV	Age	Total Cholesterol	SBP	MAP	Heart rate	HDL	AH meds	Smoking	Diabetes status
**Whitehall II**	3,432	1,021 (29.7)	0 (0.0)	77 (2.2)	0 (0.0)	0 (0.0)	14 (0.4)	77 (2.2)	5 (0.1)	72 (2.1)	130 (3.8)
**ACCT**	2,140	1 (0.0)	0 (0.0)	63 (2.9)	0 (0.0)	0 (0.0)	32 (1.5)	71 (3.3)	5 (0.2)	41 (1.9)	0 (0.0)
**BLSA**	479	5 (1.0)	0 (0.0)	13 (2.7)	0 (0.0)	0 (0.0)	18 (3.8)	14 (2.9)	0 (0.0)	5 (1.0)	0 (0.0)
**DanMONICA**	1,597	36 (2.3)	0 (0.0)	2 (0.1)	0 (0.0)	0 (0.0)	0 (0.0)	1 (0.1)	15 (0.9)	3 (0.2)	4 (0.3)
**Health ABC**	1,462	276 (18.9)	0 (0.0)	16 (1.1)	0 (0.0)	0 (0.0)	1 (0.1)	18 (1.2)	6 (0.4)	3 (0.2)	2 (0.1)
**Framingham**	3,400	700 (20.6)	0 (0.0)	79 (2.3)	0 (0.0)	0 (0.0)	497 (14.6)	79 (2.3)	9 (0.3)	1 (0.0)	76 (2.2)
**Hiroshima LA**	333	0 (0.0)	0 (0.0)	0 (0.0)	0 (0.0)	0 (0.0)	333 (100.0)	0 (0.0)	333 (100.0)	333 (100.0)	0 (0.0)
**Kinmen**	584	1 (0.2)	0 (0.0)	3 (0.5)	0 (0.0)	0 (0.0)	4 (0.7)	5 (0.9)	0 (0.0)	0 (0.0)	1 (0.2)
**EDIVA**	1,404	0 (0.0)	0 (0.0)	1 (0.1)	0 (0.0)	0 (0.0)	0 (0.0)	0 (0.0)	0 (0.0)	0 (0.0)	0 (0.0)
**CaPS**	433	9 (2.1)	0 (0.0)	28 (6.5)	0 (0.0)	0 (0.0)	0 (0.0)	28 (6.5)	1 (0.2)	3 (0.7)	0 (0.0)

3. Multiplicity: While ASCVD was clearly defined as the primary outcome, multiple secondary outcomes and subgroup analyses were presented without adjustments for multiplicity. These should be framed as exploratory or discussed with appropriate caution.

Thank you for making this point, I have made it clearer throughout the methods: "Other exploratory outcome measures considered were…", "No multiplicity was accounted for when analysing the exploratory endpoints, therefore results regarding CVD and other exploratory outcomes should be interpreted with this in mind." and results sections: "Table 1 shows the hazard ratios (HR) for each model, for the primary (ASCVD) and exploratory outcomes " of the manuscript and have added in an additional sentence to the limitations section of the discussion: "Although ASCVD was the primary outcome, other cardiovascular outcomes, including stroke and CHD were considered as exploratory outcomes, and so no adjustment for multiplicity was accounted for, so results involving exploratory outcomes should be interpreted correspondingly."

4. Observational design: The limitations inherent in observational data were not discussed. Even with large sample size and careful adjustment, causal interpretation is challenging if not impossible.

Thank you for this suggestion, that was indeed missed out of our limitations and is very important to note. I have inserted a sentence into the limitations section of the discussion which I hope will make this more transparent: “Our study does have some limitations. The observational design of this study limits these findings, we cannot claim any causal associations – however, we have incorporated large sample sizes with careful adjustment, to reduce any bias from this as much as possible.”

5. Registration of this study. It's now a common practice to register systematic review/meta-analysis studies.
Unfortunately, this review was not registered with PROSPERO, as this was considered after data analysis was already performed. However, we have ensured transparency by thoroughly documenting our methodology in the manuscript and supplementary materials. We would be happy to provide any further methodological information on request; however we feel confident that there is sufficient methodology provided in the extensive supplement.

Overall, I find the study rigorous and timely, but I recommend that the authors address these points through additional reporting and by tempering their conclusions.

VERSION 2 - REVIEW

**Reviewer**	**3**
**Name**	**Tan, Xianming**
**Affiliation**	**The University of North Carolina at Chapel Hill, Biostatistics**
**Date**	**05-Nov-2025**
**COI**	

Thanks for responding to my questions. I have no further questions/comments.
